# Data to inform a social media component for professional development and practices: A design-based research study^☆^

**DOI:** 10.1016/j.dib.2016.12.039

**Published:** 2016-12-27

**Authors:** Jeanette Novakovich, Steven Shaw, Sophia Miah

**Affiliations:** a Cleveland State University, Cleveland, OH, USA; b Concordia University, Montreal, QC, Canada

**Keywords:** Curriculum, Professional development, Identity, Social media, Instructional design

## Abstract

This DIB article includes the course artefacts, instruments, survey data, and descriptive statistics, along with in-depth correlational analysis for the first iteration of a design-based research study on designing curriculum for developing online professional identity and social media practices for a multi-major advanced professional writing course. Raw data was entered into SPSS software. For interpretation and discussion, please see the original article entitled, “Designing curriculum to shape professional social media skills and identity in virtual communities of practice” (J. Novakovich, S. Miah, S. Shaw, 2017) [1].

**Specifications Table**

**Table:** 

Subject area	*Education*
More specific subject area	*Educational Technology*
Type of data	*Text files and SPSS file, instrument, intervention, survey data*
How data was acquired	*Survey, analytics, questionnaires, and observation*
Data format	*Raw, analyzed and summarized*
Experimental factors	*Pretreatment social media survey*
Experimental features	*Design-based research study*
Data source location	*Montreal, QC Canada*
Data accessibility	*With this article*

**Value of the data**

• Informs the early stages of the instructional design process.
• Provides raw data available for comparison to other survey data involving the social media practices of undergraduates.
• Expands on results reported in original study [1].

## 1 Data

The data included in this article is intended to illuminate the framework and construction for developing curriculum in a design-based research study. The article includes examples of the course artefacts implemented in the first iteration of the design, including the course syllabus and project examples. In addition, the article provides copies of the instruments that were used to gather the data and the subsequent raw data that was collected through a survey as well as additional descriptive statistics and extends the results reported in the original article [1].

## 2 Experimental design, materials and methods

The study was conducted using a design-based research (DBR) methodology, which involves the developing, testing, investigating, and refining of the learning environment, including the tools, curriculum, activities, software, and theoretical constructs for the design (Reeves [2], p. 58). This DIB article reports on the first iteration of the study and collected data from the following sources: an initial social media survey, open-ended questionnaires, an end-of-year social media memo, Google Analytics and observations to inform the curriculum for a social media component to shape professional identity and social media skills.

A design-based research methodology was selected as a framework for this research since it allows for multiple iterations of a design to examine the learning processes that are taking place, moving beyond the superficial testing of tools and specific interventions. This DIB article provides artefacts and data from the first iteration of the study.

### 2.1 Sample

A convenience sample was selected based on enrollment in a yearlong advanced professional writing course. The sample in this DIB was taken from the first iteration of three iterations of the course design and is fully described in the article [1].

### 2.2 Intervention 1st iteration

The following course redesign is the first prototype of the course that incorporated a social media and professionalism component; its elements are derived from the literature review and subsequent design principles presented in the previous chapter.

### 2.3 Course texts

The following texts were assigned to the first iteration of the course:

• *Portfolios for Technical and Professional communicators.* Herb J Smith & Kim Haimes-Korm. Upper Saddle River, NJ: Prentice Hall, 2007.
• *White space is not your enemy: a beginner׳s guide to communicating visually through graphic, web & multimedia design.* Kim Golombisky & Rebecca Hagen. Oxford: Focal Press, 2013, 2nd Edition.
• *Style: Lessons in Clarity and Grace*, 11th Edition. Joseph M. Williams & Joseph Bizup. Boston: Longman P, 2010.
• *Writing for Multimedia and the Web, 3*rd *Edition.* Timothy Garrand. Oxford: Focal Press, 2006.

### 2.4 Course projects

The following elements were incorporated in into the first iteration of the redesign exercise. New elements are labelled as such.

• NEW: Autobiography – Students tailor this piece for the About Page on their e-portfolios.
• NEW: Oral and visual presentation– Each student is assigned to present a chapter from one of the course textbooks. Oral presentations are a necessary competency for professional writers.
• NEW: Job application project, consisting of an updated resume and cover letter. The purpose of this project is to help students articulate competencies, understand the current job market, and develop customized learning objectives for the course. To complete an authentic project, students are encouraged to find jobs that they are qualified for and interested in and to apply.
• NEW: Visual resume – Multimedia alternative to the traditional paper resume.
• NEW: Editor part 1– Formal proposal letter to the instructor for an editor position on the course community website; students visualize and plan domain specific content for the course.
• Profile – This project involves intensive research and includes a library visit and lecture from a research specialist.
• Interview – Requires obtaining a consent form and following professional interview protocols.
• Instruction set – Multimedia project using online comic strip software to present a visual rendition of a traditional instruction set.
• Feature article.
• Review article.
• NEW: Interactive multimedia project.
• NEW: Weblog – in class prewriting exercises tailored to class projects.
• NEW: Editor Part II – publishing and managing a blog on the course community website.
• NEW: Social media project – promoting publications on community blog, developing social networks and professional identity.
• NEW: E-Portfolio – professional portfolio of writing samples from the course.
• Participation – In-class work; including writing exercises, reading quizzes, participation, and class preparation.

Please see Supplementary materials for examples of Course Artefacts, including syllabus, project assignments and one example of teaching material.

### 2.5 Instruments

Instruments consisted of an initial social media survey and three sets of open-ended questionnaires. A description of how the instruments were derived can be found in the original article [1]. Please see Instruments in Supplementary material.

### 2.6 Raw data from social media survey

A survey was given to participants at the beginning of the course, prior to the start of the social media component to glean information regarding current social media practices and beliefs. See Raw Data in Supplementary material.

**SPSS Descriptive Statistics Output** for expanded information on raw data, including frequencies tables, bar charts and correlations, please see SPSS Descriptive Statistic Output in Supplementary material.
