# Supplementary material for: Data to inform a social media component for professional development and practices: A design-based research study
Source: Data Brief. 2016 Dec 27;10:544–7. doi: 10.1016/j.dib.2016.12.039 (PMC5219639; doi:10.1016/j.dib.2016.12.039)
Supplement: Supplementary file 1 — Supplementary material [file mmc1.docx]

Conflicts of interest: none

JN

SM

SS
