# Supplementary material for: Data to inform a social media component for professional development and practices: A design-based research study
Source: Data Brief. 2016 Dec 27;10:544–7. doi: 10.1016/j.dib.2016.12.039 (PMC5219639; doi:10.1016/j.dib.2016.12.039)

# Course artefacts

## Course Website

**
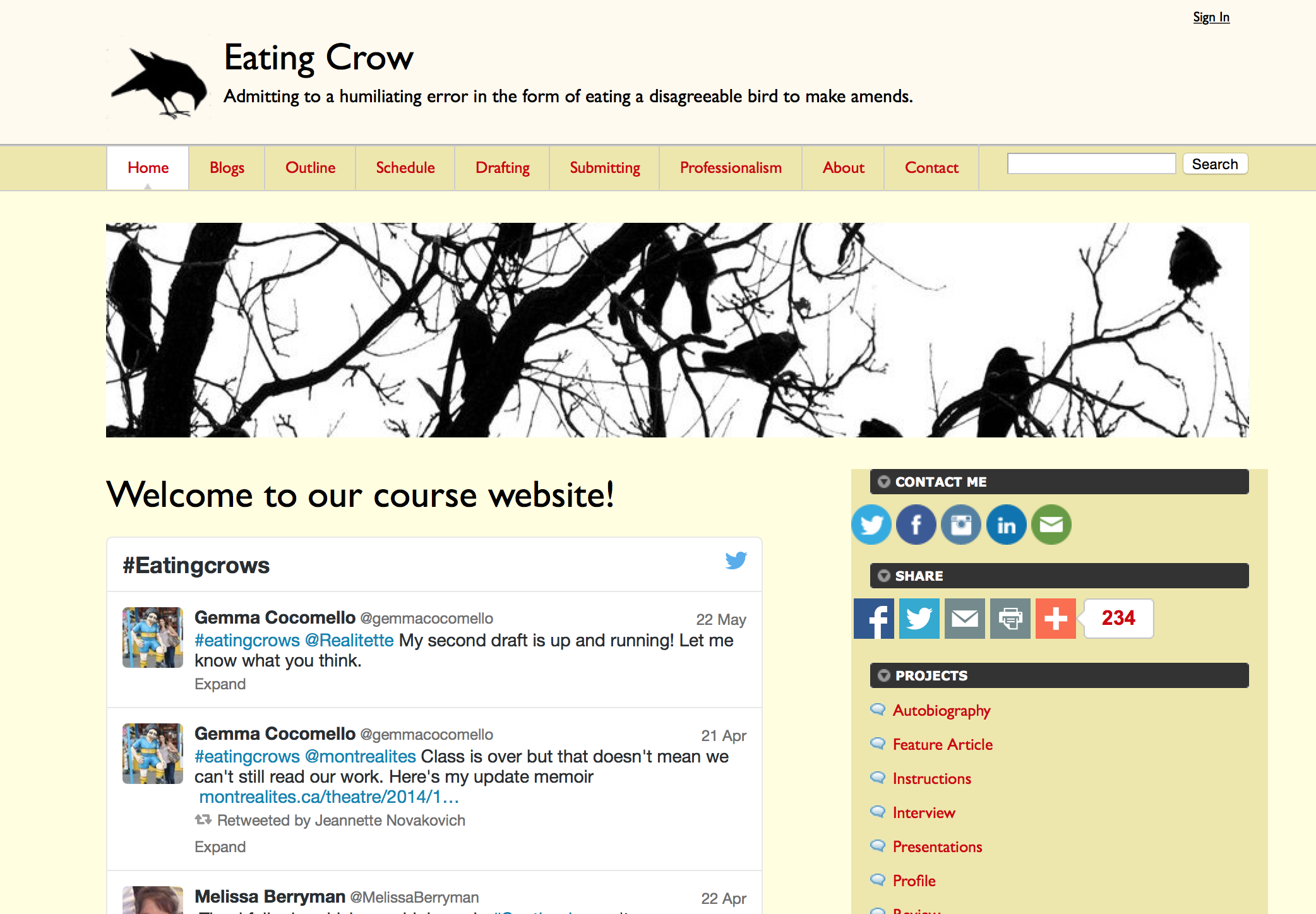
**

The course website provides the course outline, schedule, assignment sheets, grading rubrics, lecture notes, drafting, submitting, and course announcements. Students will be allowed to interact through Twitter posts fed to the course home page via a common hashtag #eatingcrows.

[**http://www.eatingcrow.ca**](http://www.eatingcrow.ca)

## E-portfolio Website

**
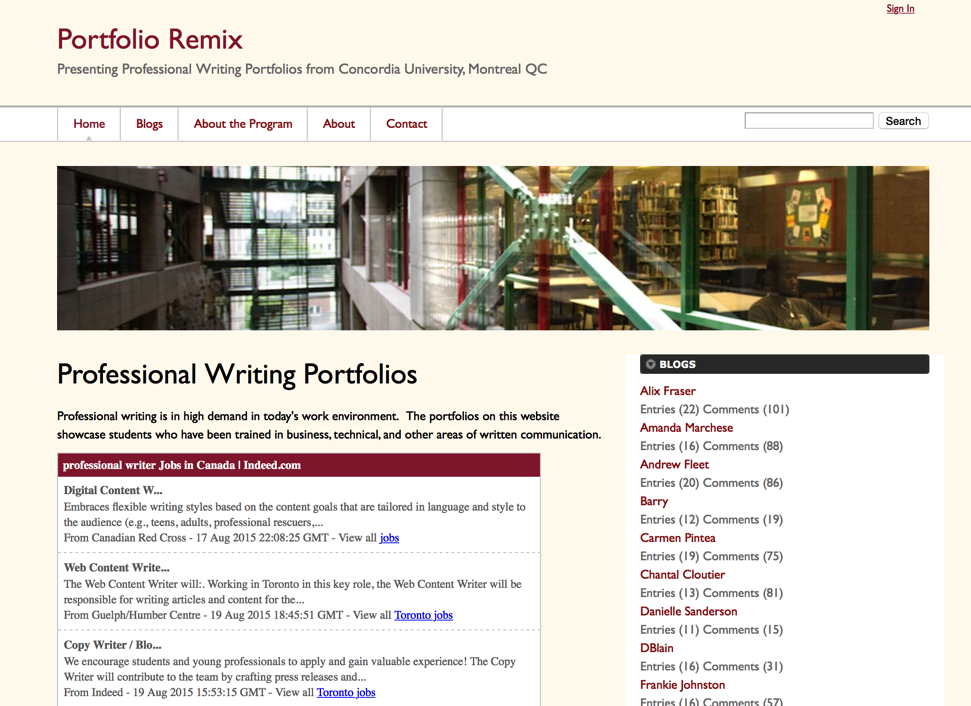
**

Students will develop process e-portfolios consisting of course projects and weekly blog entries. <http://www.portfolio-remix.com>

## Community Website

**
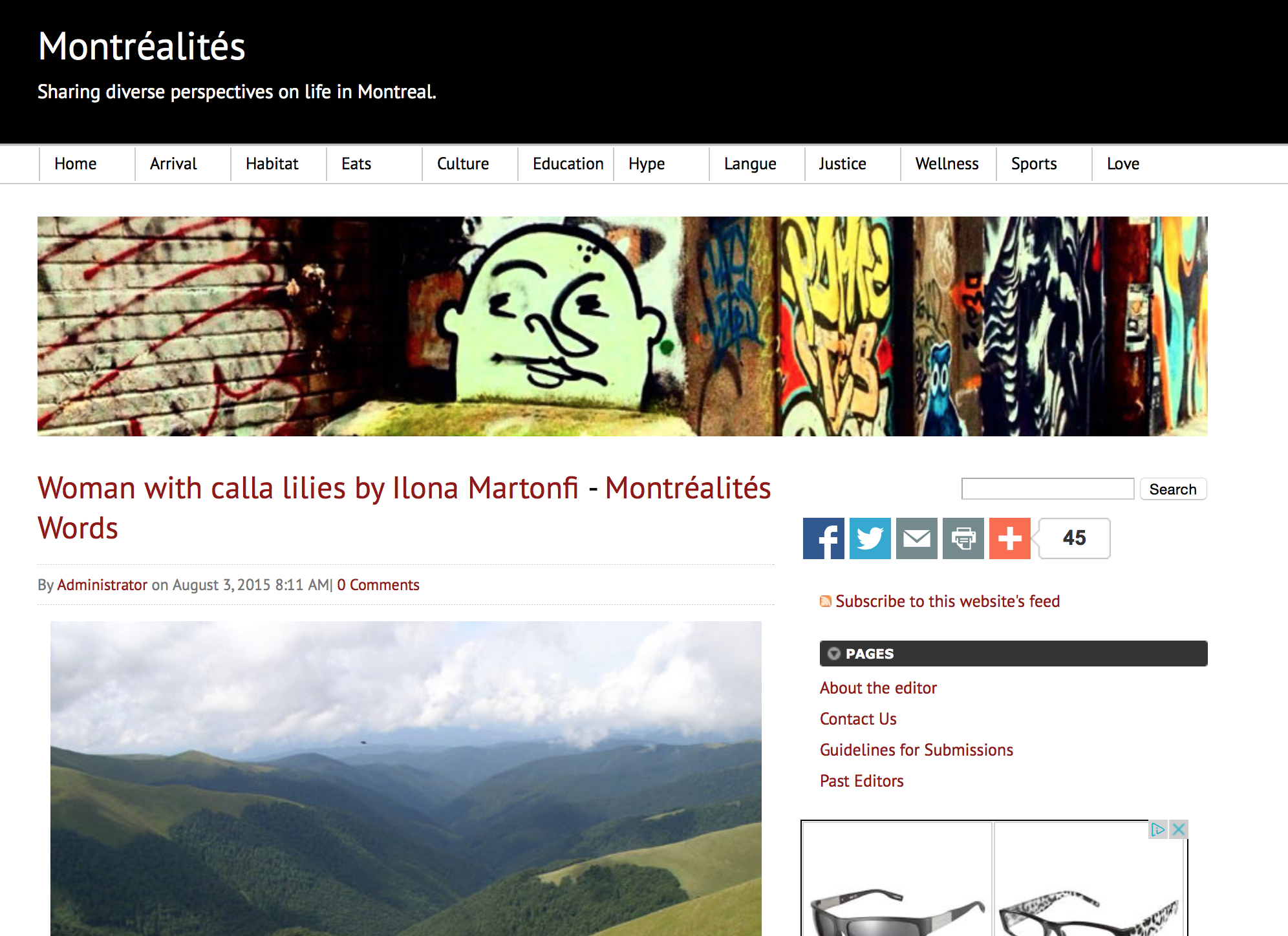
**

With a readership of 2000 plus monthly visitors, learners will have the opportunity to develop a readership within a community of practice. Google Analytics will collect readership data that students can use to guide future projects. <http://www.montrealites.com>

# Alternative Elective to Social Media

**Montrealites Editor Project:** Write a proposal for the Editor project and put together a paper portfolio of your work and substitute an "unpublished" project.

**Weblog Project:** Write ten journal entries in a diary form.

**Social Media Project:**

- SMP: Design and print 100 business cards. (keep a formal log)
- SMP: Distribute 100 business cards
- SMP: Collect at least 50 business cards from contacts.
- SMP: Write five thank-you letters to at least five of your new contacts.
- SMP: Write at least ten emails to ten of your new contacts.

**Portfolio Project:** Complete a paper portfolio.

If I missed anything, please let me know.

Each online course project page included the following multimedia items:

- An introductory image
- A description of the assignment
- Links to relevant examples and resources
- An embedded teaching PPT explaining the project
- A concluding video

# Course Outline and Projects 1^st^ Iteration

## Course Outline 2012-2013


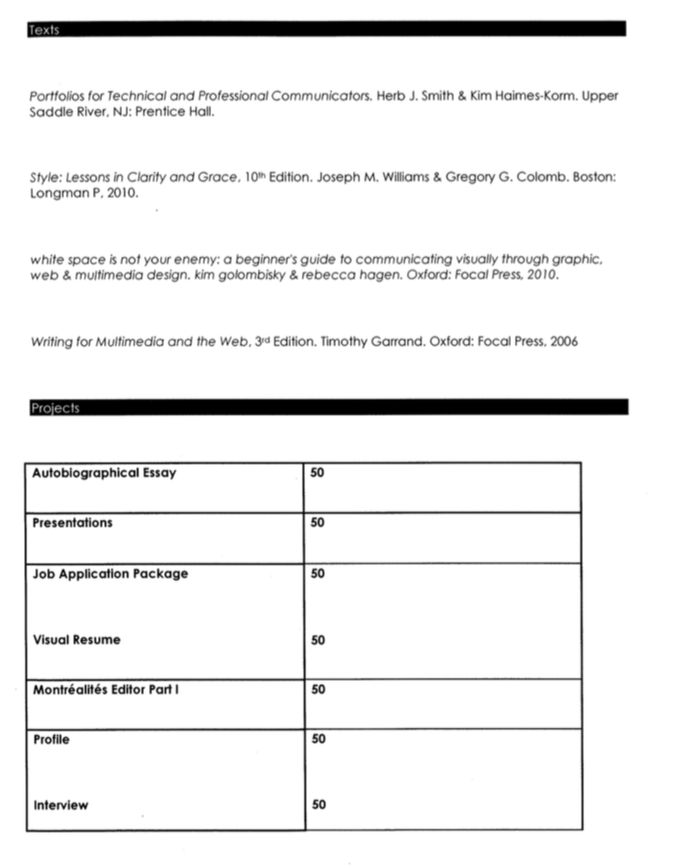


##
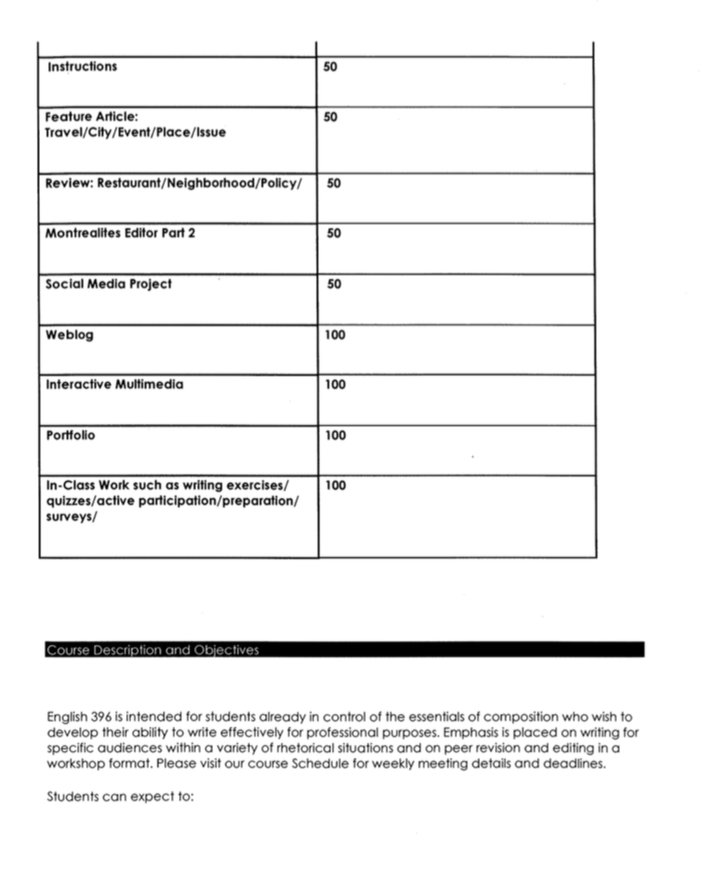


## Course Schedule 2012-2013

Before the Movable Type training course, learners will have read the following texts and completed the following tasks:

*Portfolios for Technical and Professional Communicators.* Herb J. Smith & Kim Haimes-Korm. Upper Saddle River, NJ: Prentice Hall.

*Writing for Multimedia and the Web*, 3^rd^ Edition. Timothy Garrand. Oxford: Focal Press, 2006.

Schedule:

| **Date** | **Topics** | **Reading** | **Due** |
| --- | --- | --- | --- |
| **Week 1** | Introduction to the Course  Assignments  Movable Type Introduced    Introduction to  Blogging &  Designing Portfolios    Planning workshop portfolio    Introduction to  Autobiographical writing | *Portfolios for Technical and Professional Communicators*: Chapters 1-3 and 5-6. | **Entry #1: Topic Self**  **(In class on Word.doc)**    **Portfolio Remix membership sign-up.**  **Sign-in to movable type installation.** |
| **Week 2** | Presentation: Writing for the web.    What are the best writing practices for the web. | *Writing for Multimedia and the Web*,: Chapter 2: Writing for many Media | **Sign-in to movable type installation.** |
| **Week 3** | Introduction to the Job Application Package    Resume writing    Writing cover letters  and memos  Complete the planning worksheet | *Portfolios for Technical and Professional Communicators* Chapter 8 | **Entry #2: Topic Work**  **(in class on Word.doc)**  **Autobiographical writing due**  **(on Word.doc)**    **Sign-in to movable type installation.** |
| **Week 4** | Draft workshop resume, cover letters and memo  Composing a visual resume |  | **Draft of résumés, cover letters and memo due**  **(on Word.doc)**  **Sign-in to movable type installation.** |
| **Week 5** | Presentation Lesson #4 JW Style  Write a storyboard for your visual resume    Working with Sliderocket.com and/or PowerPoint  **Movable Type Introduction** |  | **Entry #3: People**    **Verify ability to**  **Sign-in to movable type installation**  **Introduction to dashboard.**  **Publish entries on website** |
| **Week 6** | Workshop visual resume    Scribd.com - uploading your resume  Designing web space  **Movable Type Training Program: 60 minutes** |  | **Draft of Visual Résumé due**    **Publish autobiography on website’s About page**  **Publish résumé** |
| **Week 7** | Introduce Montrealites    Writing informal proposal letters    Write a proposal describing what you would like to do as editor of a section of this website. Describe your purpose, audience and goals.    **Movable Type Training Program: 60 minutes** |  | **Job application package due**    **Change your banner image.**  **Customize website.**  **Add social media widgets.** |
| **Week 8** | Presentation Lesson #7 JW Style    Writing a profile |  | **Montréalités Editor proposal letter due** |

## Autobiography

*If you're going to put your words on public display, it's your job to make that content compelling, intriguing, entertaining or informative. If it's boring... well. Suffice it to say that very few readers are going to be interested. …That's why your About page has to be just as good as every other piece of content on your site....*James Chartrand, [Five Tips for Writing Your About Page.](http://writetodone.com/2009/05/18/five-tips-and-a-bonus-on-how-to-write-a-fantastic-about-page/)

Write a personal essay for your About Page on your portfolio that analyzes the ways in which a particular experience or series of experiences contributed to your present interest in, understanding of, or attitude toward life. The essay should move beyond a mere recounting of events, but instead should analyze how an experience has shaped your identity. You will also publish this piece on Montrealites, so don't reveal personal details, your goal is to be entertaining and to create a story that embodies some aspect of your life that others will be able to relate to.

It's important to find a focus that will anchor your narrative. You may want to think ahead to how you want to use your portfolio:

- Focus on a moment of enlightenment: an event that helped you see some truth about yourself, your family, or the world for the first time.
- Focus on a moment of confrontation with the unknown or with people or situations that tested your values or challenged your identity in some way.
- Focus on a situation where you found yourself included in or excluded from a particular group or community.
- Focus on a moment of conflict between your own experience and conventional wisdom: doing something that couldn't be done, failing or struggling with something described as easy, finding value in something rejected by society.

Don't just stick to your basic credentials, degree and studies. Share some transparency; something unusual that explains how you made it here. Keep it concise and appropriate, make it honest, and tell a good story. Conclude by answering the question, how has this experience shaped your present interests or behaviors?

## Portfolio

More and more employers are asking applicants if they have a website or other web presence that they can access easily. By using material you develop for your traditional résumé and then considering what additional online elements you might use, you can develop a rich alternative for employers who want to see beyond your one-page résumé.

Remember, when you post your resume online, you are creating a "digital tattoo" or impression of yourself. When creating an online Identity, ask yourself the following question: How do you want a potential employer to see you?

**The Benefits of an Online Resume**

- Having an online professional presence
- Providing an alternative easy to access resume
- Moving beyond the one-page limitation
- Adding projects
- Distinguishing yourself from the pool
- Matching a name to a face
- Providing links

The following slideshow presents an overview of why you should make a portfolio and how you might want to develop the overall organization:

**Portfolio Requirements**

 The table below describes items that you might want to include in your portfolio.

**Designing Your Portfolio:**

- Home page
- Ten blog entries
- Embedded pdf Curriculum Vitae
- Possible projects page with optional screen shots of published work accompanied by brief descriptions and/or pdfs of print projects and any other professional writing project that you've completed
- About page- autobiographical narrative
- Contact page- email information
- Visual design, include images/screenshots (cite the sources of your images/url minimum)
- Consistent font style
- Easy navigation

**Procedure**

Step 1:  Write a home-page introduction and provide a media event such as a slideshow.

Step 2: Create a narrative biography on your About page.

Step 3: Create a simple Contact page (do not include personal information) .

Step 4:  Create a page for your Resume/Embed Pdf using Scribd.com, online hosting service.

**Embedding a Pdf File**

1. Save resume file as PDF

2. Scribd.com- open an account

3. Upload PDF to scribd

4. Open the new scribd pdf and select the Share button on Scribd toolbar

5. Select Copy the embed code.

6. Return to your Dashboard for the blogs/Open Manage Page/Resume.

7. Select <A> and paste the embed code

8. Preview the page

9. Save the page

Step 5:  Create pages for your e-portfolio.

Step 6:  Search Flickr for creative common licensed images to create an attractive and consistent visual argument.

Step 7:  Integrate any media projects that you have completed

Note: After generating ideas for your About Page, set to work drafting and polishing, crafting concisely vivid sentences and choosing each word with careful consideration. What impression do you want to create? Consider adding a professional image of yourself. Don't use prom pictures or weird cut outs. And finally, make sure that the overall impression is vibrant and worthy of notice.

Step 8: Write a headline for your resume.

Step 9: You might want to include some of the following information on your website:

- How did you choose your major?
- What is your ideal job?
- What accomplishment are you most proud of?
- What is the most significant project that you have completed?
- What role do you like to play on teams?
- Why should someone hire you?
- How do you approach a difficult project?

Warning! When Creating an Online Identity:

Protect yourself from stalking: minimize information about where you are (personal address) and what you are doing (schedules)

Protect yourself from identity theft (don't offer personal information)

## Personal Blog

This assignment requires you to write ten professional blog entries.

Blogs as a genre are more informal and immediate than most other forms of professional writing. The purpose of this assignment is to use that informality, as well as the capacity for linking to a variety of online media and to think reflectively. The goal is to engage more thoroughly with your own discipline and the role writing will have in your professional life. Blogging is your opportunity to join the read/write digital culture of the 21st century. Furthermore, it will enhance your social media skills.

Prompts are meant to be broad and open to interpretation. You will find the topics and due dates on the course schedule.

- Topic 1: Self
- Topic 2: Work
- Topic 3: People
- Topic 4: What do you do best?
- Topic 5: Etiquette
- Topic 6: Ethics
- Topic 7: Faves
- Topic 8: Stories
- Topic 9: Design
- Topic 10: Final Thoughts

**Creating an Entry**

- Step 1:  Brainstorm your topic.
- Step 2: Research online for professional articles relating to your topic. Consider using library databases.
- Step 3: Write a provocative title for your entry, one that will garner positive attention.
- Step 4: Search Flickr for an image that supports and adds interest to your entry.
- Step 5: Download image. Consider saving image with the URL of the Flickr image as the title, so that it will be easy to find later when you cite your source.
- Step 6: Upload image to your entry.
- Step 7: Write your entry, making sure to create a smooth and informative link to your image source and other outside conversations and make sure to provide concrete examples.
- Step 8: Consider searching Youtube for a supporting video to add interest and charm.
- Step 9: Embed the video.

**Notes on Writing for the Internet**

- Structure documents like an upside down triangle
  1. make big points first
  2. answers before explanation
  3. summary before details
  4. conclusions before discussion
- People don't read whole documents on the web: skimming / browsing are common, so make web documents easy to scan.
- Link to content rather than adding information, this allows readers to choose what they are interested in and builds trust by suggesting that you've done research

**Grammar / Mechanics**

- Keep paragraphs short (25 to 35 word range)
- Rely on simple sentences (under 20 words)
- Use present tense
- Establish a sense of community with the you and we voice
- Avoid legalese ("the applicant", "the customer").
- Use positive constructions.

**NOTE:** This is a compliance project, if your entry is complete and complies with the above requirements; you will receive an A for the entry. For each item that is missing, you will lose 20%.

## Job Application

Most people obtain jobs through a multi-stage process. First you research the types of jobs you are qualified for and the types of employers you would like to work for. Then you try to convince specific employers to consider you for a job. These days, most employers have too many applicants per job to interview each personally. Employers sort through job application packages (resumes and cover letters) to decide which applicants to consider further. Your first communication with your future employer is likely to be in writing and must persuade him or her to continue the conversation.

For this assignment, you will create:

- One cover letter and tailored resume addressed to a specific prospective employer/internship. The letter should highlight different aspects of your experience relevant to the job listing. The tailored resume may well differ significantly in content or in layout or both from your conventional generic resume. The choices of content and layout should emphasize appropriate experience for a specific job posting.
- A memo addressed to me that reviews what you know about the particular employer, and describes the strategies and tactics you have used to adapt your letter and resume to the specific job posting. I expect you to make good use of the information in this memo in the arguments you present in your cover letter to the employer.

**The Job Application Process**

**Step 1:** Know yourself. If you are lost, fill out the [Skills Inventory Worksheet.](http://www.personal.psu.edu/jun3/blogs/202d/skills_inventory_worksheet.pdf)

**Step 2:** Know the job market.

**Step 3:** Know the job application process. Find out how it works in your particular field.

**Step 4:** Design a resume for each specific job listing/situation.

**The Conventional CV**

The purpose of the resume is to describe your qualifications for work.

**Content.** Your resume should include contact information and relevant details of your educational training, professional training, special accomplishments, and skills. A resume is not a life history. The goal is to argue that you are qualified for a particular type of job and that you would be a capable, responsible, and personable employee who communicates effectively.

**Format.** Your format may be traditional or innovative as long as it is appropriate and as long as the information is highly accessible and is organized in a way that highlights the most important items--from the employer's perspective.

**Style.** Your style should be fairly formal. You need not use complete sentences, but you should use a concise, active style and show consistency in expression from section to section.

**Cover Letter**

While your resume is addressed to any employer with a certain type of job opening, the cover letter is most effective when tailored to a particular employer. The purpose of the cover letter is to persuade that specific employer to grant you an interview. The tailored resume may well differ significantly in content or in layout or both from your standardized one-size-fits-all resume format. The choices of content and layout should emphasize appropriate experience for a specific job posting. Just as you appreciate being treated as an individual rather than as a statistic, so does an employer. Are you applying hit-or-miss to every company in the country? Or have you invested some effort into finding a company that you are well suited for?

**Writing a Cover Letter**

**Content and Organization.** The opening of your letter should establish why you are writing to your reader. Be explicit about the fact that you are looking for a particular kind of job and explain why you would like to work at that particular company. Preview the body of the letter by stating your major qualifications for the job. The body of the letter develops each qualification with specific evidence. The goal is to show the reader both that you know what that specific company needs and that you have what it takes. You may organize this section in various ways: around your training and experience, around what the job or the company requires, or some other way. The letter should close by inviting a response.

**Style**. Cover letters are difficult to write because they aim at somewhat conflicting goals. On the one hand, you want to make a good first impression. So you want to sound polite and fairly formal. On the other hand, you want to stand out from the crowd--otherwise, why should the employer hire you rather than any of the other applicants? The best policy is probably to talk to your reader as directly and naturally as possible. Avoid hype.

**Format.** Use a conventional business letter format. Be brief: if possible, stick to one page.

**Cover Memo**

Write a brief memo (no more than one page, single-spaced) addressed to me that will help me read, understand, evaluate, and "coach" your resume and cover letter. The memo must contain a job description and audience analysis, as well as a commentary highlighting how you adapted your resume and cover letter to the job. You should look over it carefully at the very end to make sure that it tells me "how to read" your resume and cover letter.

**Writing a Memo**

Use the following outline for your memo:   To:  From:  Subject:  Date:   The purpose of this memo....   Heading: Job Description   Heading: Audience Analysis   Heading: Rhetorical Analysis   Heading: Recommendation

**Job Description.** You may base your job description on job listings that you find in a professional or trade journal, on the Internet, or in other resources on campus at Career Services.   You may also write for a summer job, an internship, or for a scholarship or other award. Note that you must hand in copies of the job ad you use.

**Audience Analysis.** Investigate the particular company you are applying to. You may obtain information on the company from the library, on the Internet, from Career Services, or other places. You may also contact the personnel office of the company directly. Then write one or two paragraphs that specify any special qualities or experience that this company may be looking for in its employees. For example, suppose you are applying for a job as a chemical engineer. A small company may be looking for an engineer who can work on a variety of projects, while another may be looking specifically for someone who has experience with polymers. This is also the place to describe anything you know about the particular person you are writing to.

**Note:** I expect you to make extensive use of this information in your cover letter. It might also have a big impact on the organization and choice of details in your resume.

**Rhetorical Analysis.** Describe how you will adapt your resume and cover letter for its particular type of job, company, and reader and why you will make those changes. Normally, your reasons will be closely related to the information in the job description and audience analysis.

When the assignment is complete, you will need to post a PDF copy of your resume and job application to your Portfolio.

**Standards for Correctness**

Employers impose strict standards of correctness on application materials: An error is the equivalent of a bad spot on your shirt. Accordingly, I will mark this assignment on a somewhat stricter scale than usual. If any letter or resume contains more than two typographical or grammatical errors, I reserve the right to fail the entire package.

- [Planning Worksheet](http://www.eatingcrow.ca/planning-worksheet-job-application.html)
- [Draft Worksheet Cover Letter and Tailored Resume](http://www.eatingcrow.ca/draft-worksheet-job-application.html)
- [Grading Rubric](http://www.eatingcrow.ca/grading-rubric-job-application.html)

## Visual CV

For the most part, a digital story blends 21^st^ century media culture with traditional narrative in a short 30 second to 5 minute video or slideshow to evoke thought, feeling and perhaps even empathy. Ultimately, it helps us connect. If we can create a memory of who we are at this time in our lives that raises awareness above the thin layer of the page, we might move people to value our progress and path as much as our final product.  Adding the digital story to the online resume creates an awareness of being, rather than simply of having been. It engages the world by producing a new way for us to talk about ourselves, our lives and our dreams. For this project, you will be creating a digital story of your life, a somewhat intimate insider's portrayal of how you live.

Why make a **visual resume**? Almost every employer requires a cv, but creating a visual CV can help you stand out and land a job.  More employers are seeking to know a potential employee on a personal level and visual resumes can give employers this insight.

**Digital storytelling** combines video, images, music, and spoken word to tell a story in a short video.

Digital storytelling focuses on seven elements:

- Point of view
- Dramatic Question
- Voice
- Pacing
- Soundtrack
- Economy
- Emotional Content

 The best digital stories are at once both personal and universal.

**Step 1: Decide on the Story You Want to Tell**

Compose a story about what you do, a place in your life, an event in your life, or about someone important.

**Step 2: Begin Writing Your Script**

Sketch out a script that you'll record with your own voice. Resist the temptation to take the easy way out and create a story with only images and music. People want to hear a personal voice. Don't be self-conscious about how your voice sounds; we all think we sound odd on tape.

Draft a short script. That's where many people get bogged down. Get past the fear of committing words to paper.

**Step 3: Create a Storyboard**

Professionals have used storyboards for decades to plot out the sequences of events that unfold in a movie, TV show, cartoon, or commercial. This is where you'll plot out your visual materials to make them align with your voice-over.

A storyboard is simply a place to plan out a visual story on two levels: 1) Time -- What happens in what order? and 2) Interaction -- How does the voiceover and music work with the images or video?

A good rule of thumb is to use no more than 15 images and no more than two minutes of video. As a general rule, four to six seconds is the ideal time for an image to appear on-screen.

**Step 4: Gather Your Materials**

**Step 5: Prep Your Equipment**

**Step 6: Digitize Your Media**

You can begin this process earlier, but be aware that the production work involved in creating a short personal story can take many, many hours. Set aside enough time to do it right.

If you're using old photos, you'll need a flatbed scanner. Scan them and save them to a single folder on your computer. If you're using digital photos, make sure they're in JPEG format.

**Step 7: Record a Voice-Over**

You may decide that the microphone built into your laptop or desktop computer will suffice for recording your narration.

Many software programs are available to capture audio from an external sound source like a microphone. The free, open-source program [Audacity](http://audacity.sourceforge.net/) can capture sound from either a computer's built-in mike or an external microphone.

Above all, speak slowly in a conversational voice. Don't make it sound like you're reading from a script.

**Step 8: Add Music**

Choose music that evokes the rhythm and pace of your story. For many people, this is the easiest part of the process. Most of us have soundtracks running in our heads that reflect the mood of the story we want to convey. The most effective tracks are often instrumental: classical, ambient, folk or jazz, with no vocals.

**Step 9: Edit Your Story**

Make sure you have all the elements of your story in your video-editing program. If you haven't done so already, import all images, video, your voice-over, and musical elements.

Next, bring the images or videos down into the timeline to match the layout of your storyboard.

It's time to create an initial rough cut before adding transitions or special effects. The draft version gives you an overview of your project and spotlights areas where images or video are insufficient to carry the story.

Next, add titles to the beginning and end of your story. You may also want to overlay text onto an image or video.

Next, add transitions -- a simple cross-dissolve generally works best -- and altering the length of each visual element to make sure it corresponds properly with the voice-over. Often, storytellers find that the "Ken Burns effect" on a Mac is a good way to add visual interest to an image, panning across and zooming into a photo to highlight an expression or important element.

Expect to spend a few hours editing your story to get it just right. Don't overproduce: often the spontaneity and directness of the initial drafts get lost with too much polishing.

**Step 10: Share Your Story**

When you've completed your video, upload it to a video hosting service and publish it to your portfolio.

In addition to telling the story of your passion or interest in your studies and future career, videos or slideshows must fulfill the following guidelines:

- Be 30 seconds to 10 minutes in length.
- Be made in as high a quality as you can.
- Contain original work, creative commons licensed material, public domain material or permission to use copyright protected material

## Presentations

For this assignment, you will be presenting one chapter from Joseph Williams' *Style: Lessons in Clarity and Grace or* Kim Golombisky & Rebecca Hagen's *white space is not your enemy*. Each student will be assigned one chapter.

This assignment requires the following items:

- Produce a thirty-minute oral and visual presentation on your assigned chapter
- Integrate at least four challenging exercises

The following example uses SlideRocket.com. Consider using Slideshares or Prezi to host your presentation. The presentation will take you several hours to complete. Don't wait until the last minute. No late presentations without a medical note will be allowed.

## Original Social Media Project

For this project, you will be required to complete the following items:

- Capture a before and after screen shot of your name's Google search results 3 times during the semester:
  - Beginning
  - Middle
  - End
- Sign up with at least three social media websites and create profiles, engage followers and make social connections (Foursquare, Twitter and LinkedIn.
- Set up metric tools for your portfolio to measure site traffic (Google analytics or sitemeter).
- Measure your overall efforts
- Write a memo to me summing up the experience

Identify Goals

Think about whether reach, reputation or engagement is your goal.

Write down *specific* objectives related to your social media efforts. For example, a goal could be to get more social media mentions of your blog, increase readership, or build reputation. Goals encourage you to be efficient and focused with your social media efforts.

In any case, the first step is to Google search your name in quotes "Your Name" and screen capture your Google results.

Screen-capture your web presence at the start of the project, midway through the course and capture the results one last time before you hand in the social media assignment (include all three screen shots).

Set up Google alerts for your name

Establish Credibility

Create a consistent social media identity. Include the same picture, credentials, and URLs for your ids.  Sites can include LinkedIn, Twitter, Flickr, FourSquare, YouTube, Vimeo, Google Profile, SlideShare, Delicious, Digg, Reddit, or any website of your choice with the exception of Facebook.

Find influentials on social media sites.

Listen. Each topic and application has its own culture.

Interact with others users. Focus on becoming part of the read-write web.

Social Media Monitoring

Search social media monitoring sites for mentions:

- [Addcitomatic](http://addictomatic.com/) Look up your twitter id and full name in quotes
- [Google Alerts](http://www.google.com/alerts) Set up an alert for your name or blog in quotes
- [SocialMention](http://www.socialmention.com/) Tracks mentions related to your name

Set up metric tools:

- [TweetStats](http://tweetstats.com/). Provide your average tweets per month.
- [Sitemeter](http://www.sitemeter.com/) (Traffic analyzer). Track your blog site traffic
- [Google Analytics](http://www.google.com/analytics/) Provide the visitor number for each month.
- [Feedburner](http://www.feedburner.com/)  Provide the number of people who subscribe to your blog via RSS.

 Measure Efforts

Beginning of project:

| Social Media | Followers/  Connections | Number and types of Posts | # of Responses to posts |
| --- | --- | --- | --- |
| Linked-In |  |  |  |
| Twitter |  |  |  |
| Choice |  |  |  |

 Middle of project:

| Social Media | Followers/  Connections | Number and types of Posts | # of Responses to posts |
| --- | --- | --- | --- |
| Linked-In |  |  |  |
| Twitter |  |  |  |
| Choice |  |  |  |

 End of project:

| Social Media | Followers/  Connections | Number and types of Posts | # of Responses to posts |
| --- | --- | --- | --- |
| Linked-In |  |  |  |
| Twitter |  |  |  |
| Choice |  |  |  |

**Monitoring Social Reputation Building**

Where do you best fit in terms of social media reputation building and why?

Which applications did you most enjoy?

What are your social media goals?

Which applications serve your goals best?

How will social media influence your career?

Why is relationship building important?

How is online content different than offline?

What were your perceptions before and after this assignment?

| **Social Media** | **Details** | **Excellent** | **Good** | **Poor** |
| --- | --- | --- | --- | --- |
| **Linked-In # of**  **connections** |  | 10 | 5 | 1 |
| **Linked-In**  **completion/content** |  | 100% | 50% | 25% |
| **Recommendations** |  | 2 | 1 | 0 |
| **Twitter** **# of Followers/**  **connections** |  | 50 | 25 | 0 |
| **Numbers and types of Tweets** |  | 25 | 10 | 0 |
| **Social feedback from followers/friends** |  | 5 | 3 | 0 |
| **Choice # of Followers/**  **connections** |  | 10 | 5 | 0 |
| **Choice: #'s   interaction/badges** |  | 5 | 2 | 0 |
| **Choice: Social feedback followers/friends** |  | 5 | 3 | 0 |
| **Include 3 Screenshots of process** |  | 3 | 2 | 0 |
| **Includes a memo with a summary and analysis of the experience** |  |  |  |  |

## Montrealites Editor Project 1

For this project, you will write a formal proposal letter seeking my approval for the Montréalités Editor Project. The proposal is the first document in a sequence leading up to the end-of-year completion of the course. As an editor of a section of Montrealites of your choosing, you will develop a number of projects. After your proposal has been approved, complete the Montrealites Editor Bio information and send it to me. After you have completed and turned in the proposal and bio, you will receive a grade for part 1 of the Montréalités Editor Project.

The sequence of projects that you will complete as editor will include the following works:

- Profile
- Interview
- Instruction set
- Feature article
- Review
- Interactive web project
- Manage social media interaction

When writing the proposal, think of me as someone who wants to be sure that you are choosing a project that you actually want to do and one in which you can do a good job. What you will be describing in your proposal is your vision. I need to be convinced that this project is important and that you have the ability to run it.

**Proposal Format**

The format of this assignment should be that of a formal business letter. Protocols for business letter composition are provided below. Select your information and organize it in such a way that it is persuasive and accessible. Include the following items:

- **An introduction** that tells me why you are writing.
- **A section on the Website**, including an explicit well-developed mission statement.
- **A description of the section of Montrealites** that you want to take over or establish, describing your plans for researching the subject matter and developing your section. Convince me that you know what kind of information you'll need and where to find it.
- **A description of your goals** for this project
- **A discussion of your credentials.** Convince me that you have the background and resources necessary to conduct your research.
- **A schedule.** Convince me that you know what activities your research will require and that you can get them done on time.
- **A conclusion** that formally requests permission to proceed.

You should probably begin your letter by convincing me that a significant need exists that calls for the website section or revision that you propose. In short, how will your section increase web traffic? After you have convinced me of a need for your work, include a detailed description of your work plan.

Convince me that this plan for research is the right path and that the time exists in this semester to do the work well.

This work plan must also be plotted with time; you must indicate what work you will be doing during each of the weeks left in the semester.

Format as a letter:

- Use block style.
- Employ headings and lists to render your information readily accessible

**Further Resources**

Explore the following Web sites for further information on this lesson's topic:

- "[Short Course" on proposal writing (from The Foundation Center)](http://fdncenter.org/learn/shortcourse/prop1.html) <http://foundationcenter.org/getstarted/tutorials/shortcourse/index.htmll>
- "[Ideas are a Dime a Dozen, So Why Should I Listen to Yours? 'Pitching' Your Ideas So That They Will Be Heard](http://www.stc.org/confproceed/2000/PDFs/00049.PDF)" <http://www.stc.org/confproceed/2000/PDFs/00049.PDF>
- Image source: [Flickr.](http://www.flickr.com/photos/patries71/261657070/#/photos/patries71/261657070/lightbox/)

**Creating your Montréalités Editor Bio**

Paste your info in the highlighted areas of the text box below. When you have completed the task, publish it to your process portfolio.

- Yellow highlight: upload a picture and copy a link to its location.
- Green highlight: substitute your name.
- Blue highlight: copy the url of your LinkedIn profile page.
- Purple highlight: copy the url of your portfolio page.
- Red highlight: substitute your Twitter id.
- Blue highlight: a mini version of your autobiography

<a href="http://www.montrealites.ca/Screen%20shot%202011-01-29%20at%2010.33.27%20PM.png"><img alt="Screen shot 2011-01-29 at 10.33.27PM.png"src="http://www.montrealites.ca/assets_c/2011/01/Screen%20shot%202011-01-29%20at%2010.33.27%20PM-thumb-75x73-853.png" class="mt-image-left" style="float: left; margin: 0pt 20px 20px 0pt;" height="69" width="70" /></a> Jeannette Novakovich<br /> <a href="http://www.linkedin.com/profile/view?id=82928836&amp;trk=tab_pro"><img alt="Screen shot 2011-03-05 at 7.21.06 PM.png" src="http://www.montrealites.ca/Screen%20shot%202011-03-05%20at%207.21.06%20PM.png" class="mt-image-left" style="float: left; margin: 0pt 20px 20px 0pt;" height="41" width="41" /> </a><a href="http://www.jnovakovich.com/"><img alt="portbutton.png" src="http://www.montrealites.ca/portbutton.png" class="mt-image-left" style="float: left; margin: 0pt 20px 20px 0pt;" height="39" width="45" /></a><a href="http://www.twitter.com/Realitette"><img src="http://twitter-badges.s3.amazonaws.com/t_logo-a.png" height="41" width="41" /></a><p class="MsoNormal"><a href="http://www.twitter.com/Realitette"><br /></a></p>

Add text for your bio here.

## Montrealites Editor Project 2

For this part of the project, you will be acting as editor of a section of Montréalités. In this capacity, you will publish a number of projects, measure and record Google Analytics of your published work, and integrate an active social media presence on the web, including, Twitter microblogs, Facebook updates, and a Pinterest board.

## Profile

This assignment will give you practice composing a descriptive and informative essay about a particular person (image source [Flickr](http://www.flickr.com/photos/25559122@N06/3739502813/sizes/z/in/photostream/)).

In an essay of approximately 800 to 1000 words, compose a profile of an individual whom you will interview in a follow-up project. The person may be either well-known in the community (a politician, a local media figure, the owner of a popular night spot) or relatively anonymous (a Red Cross volunteer, a server in a restaurant, a school teacher or college professor). The person should be someone of interest (or potential interest) not only to you but also to readers of Montréalités.

The purpose of this essay is to convey--through close observation and factual investigation--the distinct qualities of an individual.

**Composing Strategies**

**Getting Started.** One way to prepare for this assignment is to read some engaging character sketches. Look at recent issues of any magazine that regularly publishes interviews and profiles.

**Choosing a Subject.** Give some serious thought to your choice of a subject. Remember that you're not at all obliged to choose a person who's socially prominent or who has had an obviously exciting life. Your task is to bring out what's interesting about your subject--no matter how ordinary this individual may at first appear.

Keep in mind, however, that the present occupation of your subject may be inconsequential; the focus of the profile may instead be on your subject's involvement in some notable experience in the past.

**Writing a Profile Template**

A profile isn't a biography. It isn't a recounting of a person's work history. It's a story about some interesting aspect of a person's life.

**Key elements**

***An interesting lead***. You need to hook the reader right away. Try to capture the reader's attention and then keep it.

***Lively quotes***. The reader needs to know you were there. You interviewed your subject. This isn't some second-hand story taken from a press release or a web site.

***Anecdotes***. Funny, sad, touching or dramatic anecdotes will boost your story's appeal. Profile stories should entertain and inform.

***Perspective***. What makes your subject worth a profile? If you are profiling a hairdresser, what made you pick this hairdresser? What makes your profile subject special? You should give your reader a clue high in the story why you are doing the profile. Sometimes it's because a subject is exceptionally interesting. Sometimes it's because the person has sudden been thrust into the news.

***Background***.  You don't need to tell your subject's entire life story, but give some idea of the person's journey.

***Color***. Some description of your subject. What does he or she look like?

***A logical flow***. Add transitions between paragraphs when needed.

***Another point of view***. Interviews with at least two people who know your subject are required. These will help enrich your profile. Keep in mind that profiles aren't necessarily puff pieces. Just because you are writing about someone doesn't mean you work for the person or are the person's public relations agent. What matters is that you try to tell the truth. Your story should have balance. If a politician with a supposedly spotless record turns out to be a child molester - and you have some evidence or proof - then that's your story.

***Depth***. Profiles sometimes take on an investigative nature. Some reporters are known to do dozens of interviews for in-depth profiles of a single person. That's not what I'm expecting for this assignment, of course. But you should know that profiles should not be single-source stories. You shouldn't just take your subject's word on everything he or she says and leave it at that. You should try to bring some depth to your story.

***News and timeliness***. Profile a subject with some connection to news stories or controversial issues.

## Interview

For this assignment, you will be following up your profile with an interview. (Image source: [Flickr](http://www.flickr.com/photos/goaobserverstream/3034077568/))

**Interviewing a Subject**

- Before the interview, make a list of questions to ask your subject
- Get people talking. Learn to ask questions that will elicit answers about what is most interesting or vivid in their lives.
- Take notes during the interview. If you have trouble keeping up with your subject, just say, "Hold it a minute, please," and write until you catch up.
- Use a combination of direct quotations and summaries. "If the speaker's conversation is ragged, . . . the writer has no choice but to clean up the English and provide the missing links. . . . What's wrong . . . is to fabricate quotes or to surmise what someone might have said."
- To get the facts right, remember that you can call [or revisit] the person you interviewed.

**Drafting.** Your first rough draft may simply be a transcript of your interview. Next, supplement these remarks with descriptive and informative details based on your observations and research.

**Revising.** In moving from transcripts to profile, you face the task of how to focus your approach to the subject. Attend to key details, incidents, and experiences. Let your readers know what your subject looks like and sounds like. Build your essay on quotations from your subject as well as factual observations and other informative details.

**Editing.** In addition to the usual strategies that you follow when editing, examine all the direct quotations in your profile to see if any could be shortened without sacrificing significant information. By eliminating one sentence from a three-sentence quotation, for instance, your readers may find it easier to recognize the key point that you want to get across.

**Tips**:

- Before your interview, learn all you can about your subject.
- Google your subject.
- Bring a list of questions to the interview.
- Discover what makes your subject newsworthy.
- Develop a theme.
- Dress appropriately.
- Establish a rapport with your subject.

**Guidelines:**

- Find a subject from Montreal or of interest to Montreal readers.
- Interview in person or by phone or by email.
- Obtain a photograph of your subject.
- Make subject aware that your story will appear online.
- Include your subject's first and last name, job title or occupation, age and location.
- Talk to and quote at least two other sources who are knowledgeable about your subject.
- Use 10-point type, single-spaced Optima font.
- Provide an element of news, timeliness or human interest.
- Include quotes.
- Signed consent form.

License and Release Forms

[Montrealites.docx](http://www.eatingcrow.ca/Montrealites.docx)

## Instructions

Instruction sets are common documents for many disciplines and occupations. The main goal when producing an instruction set is to create easy to follow directions.

Keep this in mind when you produce this assignment. Your objective will be to create a professional instruction set for Montréalités in the form of written instructions and a comic strip. The comic strip is the essence of concise visual writing. Thus, for this project, I would like for you to produce a project that adapts an instruction set to the conventions and aesthetics of a comic strip.

To complete this assignment, you will complete a written instruction set and create a minimum of a ten-frame or ten-step comic strip for Montrealites based on the theme: "Montrealites Guide to Survival". The comic strip does not have to be humorous; instead, it needs to illustrate and make a point about something important in Montreal.

**Objectives**

- To adapt technical information to reach a larger audience
- To gain expertise creating an argument that is largely visual.
- To understand the basics of typography and color choices when designing documents

Be sure to put a title and your name in the text box above the comic strip. Feel free to use the online comic strip maker: [Pixton Comics](http://www.pixton.com/ca/login#video).

montrealites@montrealites.ca

montrealites

montrealites/fluffy

**Procedure**

You will develop a set of instructions advising users on how to perform a specific task that one can complete in Montréal.

**Guidelines**

- Choose something you are very familiar with and that is related to your section of Montréalités
- Ideally, your audience should be someone who has never performed this task before.
- Your audience should have a general understanding of the topic area.
- Choose a task with an appropriate level of difficulty--neither too easy nor too hard to explain in the space allotted.
- The task may involve a some type of process (e.g., applying to a university).
- The process should have discrete parts or steps that are fairly easy to name and refer to.

**Topics**

Here are some topics that you might want to choose for your project:

- How to park downtown Montreal
- How to bake a three-layer chocolate fudge cake
- How to register your car
- How to break a lease
- How to speak Quebecois
- How to choose a night club

**Rhetorical Situation**

Before you begin to write, consider the rhetorical situation for your instructions. Use the planning worksheet to help you determine the purpose, audience, context, and content for your instructions. Note, people have unique ways of learning. Take the [Vark Questionnaire](http://www.vark-learn.com/english/page.asp?p=questionnaire) to learn about your style of learning. Next, consider how your audience might best learn. Consider the following video and reflect on the type of instruction that would be most effective for this computer user.

Depending on the nature of your task, you may wish to include some or all of the following contents.

What your instructions should include:

- An overview of the steps needed to complete the task
- Definitions of terms or concepts they need to know before they proceed
- Cautions or warnings that apply to the task as a whole
- A sense of how long the task will take
- Where they should perform the task
- List of materials or ingredients needed.
- List of steps in chronological order.
- Each step should include a diagram, drawing, photograph, or figure.
- Include captions for each illustration or figure.

Additional Guidelines for Designing an Instruction Set

- Make sure you use the imperative mood. ("Attach the red wire.")
- Phrase each step clearly and concisely.
- Provide "feedback" that informs the reader what will happen after they complete each step.
- Include warnings or cautions before readers encounter the problems.
- Break long lists into sections with appropriate sub-headings.
- Make sure sub-headings and steps are phrased in parallel form.
- Include troubleshooting tips.
- Provide a glossary of key terms and definitions.

**Organization**

Instructions are normally organized in a chronological order. Beyond that, here are some other guidelines:

- Provide a clear hierarchy of headings and subheadings.
- Well-chosen fonts.
- Numbered lists and bulleted lists, where appropriate. Know the difference. Make sure bullets and numbering are consistently formatted. Do not number or bullet lists with fewer than two items.
- An appropriate amount of white space--neither too much nor too little.
- Effective use of alignment. Centered alignment may make it harder for users to skim headings and sub-headings; left alignment or indentations can be more effective for this.
- Effective use of contrast. Too much contrast means that nothing stands out; too little makes it hard for users to find what they need. Consider emphasizing elements like headings, key words, and warnings.
- Consistently used design features. Decide which fonts, font sizes, and forms of emphasis you will use and apply them consistently.
- Length should be about 2 pages single-spaced.

You will produce a traditional instruction set and a comic strip one. The traditional instruction set must be complete and include an introduction, steps, and brief conclusion.

## Feature Article

A special interest article for Montréalités focuses on the people, places, events, inventions, industries, and other elements that have shaped Montreal. In terms of style, they approximate an magazine article more than anything else. Content is meant to engage the reader. They should also be well-researched; books, journal articles, first-hand reporting, even internet sources used judiciously.

Writing Feature Articles

For this project, you will be writing a feature article. This type of article differs from a straight news story in one respect - its intent. A news story provides information about an event, idea or situation. The feature does a bit more - it may also interpret news, add depth and color to a story, instruct or entertain.

**Writing the Introduction**

- Step 1: Introduce the topic without giving away the focus of the article immediately and provide a small scope of what it's about with an interesting hook.
- Step 2: Name the topic and write a sentence or two establishing its significance and where to find it.
- Step 3: State why this topic should interest the reader beyond local interest.
- Step 4: Create a segue to the definition of topic.
- Step 5: Define topic.
- Step 6: Give an extensive explanation of the event, invention, place, etc. (about a paragraph long).
- Step 7: Give enough information to give the reader an understanding of where the article is going.

**History**

- How did it start? Or where did the first idea come from?
- Describe the necessary steps taken for this idea to evolve into the finished product, while giving dates and naming the most important contributors.

**Cultural Significance**

- What sets it apart from others like it?
- Impact on society or its importance?
- Who or what has it influenced?
- Relevance today?
- Do we still see it today? If not, what remnants are visible?

**Conclusion**

- Should be catchy, upbeat (if appropriate)

**Captions**

- Make sure picture is linked to the content of the article
- Ask yourself, "Why is this picture important above all others?"
- Describe picture like you are curator of a museum. For example, “Here we see_____________." But not so blatant.
- Try to include dates.
- Describe in a sentence the action of the picture...detail about the subject.

**Titles**

- Intriguing titles are best
- Subtitles can be useful

**Points to Keep in Mind**

- Focus on human interest - Don't think about writing a science story - think about writing a human-interest story.
- Be clear about why you are writing the article. Is it to inform, persuade, observe, evaluate, or evoke emotion?
- Write in the active voice.
- Accuracy is important.
- Keep your audience clearly in mind - what really matters to them?
- Avoid clichés - especially at the end of your article.
- Interviews for features usually need to be in-depth and in person rather than over the phone - this enables you to add in color and detail.
- Use anecdotes and direct quotes to tell the story -
- Talk to more than one person to provide a more complete picture
- Three to five sentence paragraphs are best. The articles will be published using the one-column format.

## Interactive Web Project

For this assignment, you will produce an interactive web project for your section of Montrealites showcasing all that you have learned this semester. Strive to go beyond a simple linear click and read interaction. Your project should have a clear purpose and design. Consider working with members of the class and create interactivity between sections of Montrealites.

A key component of this project involves writing. This project should include at least five web pages of articles/reviews/features/instructions/profiles and so forth. It should include a minimum **3000-word count**.

The final component of the project is Interactivity, engaging your readers by seeking input from them or by simply providing an interactive element, which might include all, some or one of the following items:

- Image slide shows (not PPT!)
- Map widgets (Google)
- Tool widgets (calculators)
- Multiple web page interactions
- Parallel narratives
- Type of gaming puzzle that leads users through the an exploration of Montréal or Montréalités, using a kind of Nancy Drew gaming platform with icons, menu, and limited interaction.
- Videos
- Quizzes
- Form widgets
- Survey widgets


Supplement: Supplementary file 2 — Supplementary material [file mmc2.docx]
