# Supplementary material for: Data to inform a social media component for professional development and practices: A design-based research study
Source: Data Brief. 2016 Dec 27;10:544–7. doi: 10.1016/j.dib.2016.12.039 (PMC5219639; doi:10.1016/j.dib.2016.12.039)
Supplement: Supplementary file 3 — Supplementary material [file mmc3.docx]

## Phase 1 Social Media Survey

Which of the following online sites are you familiar with? Check all that apply.

Face Book    Twitter    Pinterest    LinkedIn    Four Square    Tumblr    Google Plus Wiki forums    Yelp    You Tube    Yammer    My Space    Word Press    Blogspot Delicious    Digg    Reddit

Which of the following online sites have you joined? Check all that apply.

Face Book    Twitter    Pinterest    LinkedIn    Four Square    Tumblr    Google Plus Wiki forums    Yelp    You Tube    Yammer    My Space    Word Press    Blogspot   Delicious    Digg    Reddit

Which of the following online sites have you posted information on? Check all that apply.

Face Book    Twitter    Pinterest    LinkedIn    Four Square    Tumblr    Google Plus Wiki forums    Yelp    You Tube    Yammer    My Space    Word Press    Blogspot Delicious    Digg    Reddit

|  | Never | Practically never | Once in a while | Fairly often | Very often | Almost always |
| --- | --- | --- | --- | --- | --- | --- |
| How frequently do you visit social media sites? | 1 | 2 | 3 | 4 | 5 | 6 |
| How frequently do you post or share information? | 1 | 2 | 3 | 4 | 5 | 6 |
| Please indicate how frequently online software technologies are integrated into your academic activities. | 1 | 2 | 3 | 4 | 5 | 6 |

On average, how many hours per week do you spend using social media sites?

None    Less than 1 hour    1 to 3 hours    5 to 10 hours    More than 10 hours

Do you have experience creating and publishing online projects?

 Yes    No    Uncertain

Select the level that best describes you:

1 Unfamiliar, I have no experience with social media

2 Newcomer in the sense that I have account(s), but rarely visit or post

3 Beginner, I make occasional postings and have made a few connections

4 Average, I have made a number of connections and postings

5 Advanced, I have competently used a broad spectrum of sites

6 Expert, I have guru status

Choose the stage that best describes where you are in the process:

1 Awareness: I am aware that social media exists, but have not used it – perhaps I’m even avoiding it. I am anxious about the prospect of using it.

2 Learning: I am currently trying to learn the basics. I am sometimes frustrated and I lack confidence.

3 Understanding: I am beginning to understand the process of using social media and can think of specific tasks in which it might be useful.

4 Familiarity: I am gaining a sense of self-confidence in using social media sites for publishing online. I am starting to feel comfortable publishing.

5 Adaptation: I think about social media as a professional tool to help me and I am no longer concerned about publishing online.

6 Creative Application: I can apply what I know about technology to develop an online reputation through publications on social media sites.

Do you understand the basic framework of website design?  Yes    No    Uncertain

On a scale of 1 to 5, with 5 being the highest level of interest, rank the following statements.

|  | 1 | 2 | 3 | 4 | 5 5 |
| --- | --- | --- | --- | --- | --- |
| On a scale of 1 to 5, with 5 being the highest level of interest, how would you rank your interest in using online software technologies? | 1 | 2 | 3 | 4 | 5 |
| On a scale of 1 to 5, with 5 being the highest level of importance, how would you rank the importance of being familiar with and confident using online software technology? | 1 | 2 | 3 | 4 | 5 |

Please rate the extent to which you agree or disagree with the following statements regarding the use of social media.

|  | Strongly Disagree | Disagree | No opinion | Agree | Strongly agree |
| --- | --- | --- | --- | --- | --- |
| Social media is harmful to your professional reputation | 1 | 2 | 3 | 4 | 5 |
| Social media is mostly used to waste time | 1 | 2 | 3 | 4 | 5 |
| Social media is effective because I believe I can implement it successfully | 1 | 2 | 3 | 4 | 5 |
| Social media promotes social reputation | 1 | 2 | 3 | 4 | 5 |
| Social media is mostly intended for personal use | 1 | 2 | 3 | 4 | 5 |
| Social media promotes the development of communication skills | 1 | 2 | 3 | 4 | 5 |
| Social media is a valuable professional tool | 1 | 2 | 3 | 4 | 5 |
| Social media is too costly in terms of risk to professional reputation | 1 | 2 | 3 | 4 | 5 |
| Social media is an effective tool for students of all abilities | 1 | 2 | 3 | 4 | 5 |
| Social media enhances my professional development | 1 | 2 | 3 | 4 | 5 |
| Social media promotes the development of interpersonal skills | 1 | 2 | 3 | 4 | 5 |
| Social media increases stress and anxiety | 1 | 2 | 3 | 4 | 5 |

## Beginning of year questionnaire

- What was your opinion of social media prior to our guest speaker? Afterwards?
- What is your understanding of social writing? Describe or map out how it works.
- Have you ever encountered any serious issues when posting online? Please describe.
- What types of information do you believe professionals can post online?
- How would you describe your online digital presence?
- What questions do you have about the course social media-writing project?
- At what level do you plan to participate in the project and explain why?
- What additional skills in terms of social writing would you like to learn?

## Midyear questionnaire

- Describe the progress you have made regarding your social media project.
- What are the obstacles you have encountered in terms of building a social network?
- Describe what has been holding you back, psychologically or socially?
- How can you overcome obstacles and barriers to your performance?
- What is your social media strategy?
- What are your social media goals?

## End of year questionnaire

- Describe where you want to go in the future in terms of building social media networks: engagement, reputation, or numbers and why?
- How would you describe your online digital presence today?
- What questions do you have about the social media-writing project?
- Define what social writing means to you and its benefits and risks.
