# Supplementary material for: Data to inform a social media component for professional development and practices: A design-based research study
Source: Data Brief. 2016 Dec 27;10:544–7. doi: 10.1016/j.dib.2016.12.039 (PMC5219639; doi:10.1016/j.dib.2016.12.039)
Supplement: Supplementary file 5 — Supplementary material [file mmc5.zip › mmc5.htm]

xml version="1.0" encoding="UTF-8"?


SMSurvey1stIterationStatisics


|  |  |  |
| --- | --- | --- |
| IBM SPSS Web Report - Output2     ---   Contents  Previous  Next  Help |  | Controls disabled by the system   --- |

- Log

  - Log
- Frequencies

  - Active Dataset
  - Statistics
  - Frequency Table

    - Familiar with Facebook
    - Familiar with Twitter
    - Familiar with Pinterest
    - Familiar with LInkedIn
    - Familiar with Foursquare
    - Familiar with Tumblr
    - Familiar with GooglePlus
    - Familiar with WikiForums
    - Familiar with Yelp
    - Familiar with YouTube
    - Familiar with MySpace
    - Familiar with Blogs
    - Familiar with Social Bookmarking
    - Aggregate of number of social media sites familiar...
    - Joined Facebook
    - Joined Twitter
    - Joined Pinterest
    - Joined LinkedIn
    - Joined Foursquare
    - Joined Tumblr
    - Joined Google Plus
    - Joined YouTube
    - Joined MySpace
    - Joined blogs
    - Joined Social Bookmarking
    - Aggregate of number of social media sites joined
    - Posted Facebook
    - Posted Twitter
    - Posted Pinterest
    - Posted LinkedIN
    - Posted Foursquare
    - Posted Tumblr
    - Posted GooglePlus
    - Posted Youtube
    - Posted MySpace
    - Posted Blogs
    - Aggregate of number of social media sites posted
    - Frequency of visits to social media sites
    - Frequency of posting to social media sites
    - Frequency social media is integrated into academic...
    - How many hours per week do you spend on social med...
    - Published original online projects
    - Level of perceived social media expertise
    - Stage in the process of social media practice
    - Understand website design and publishing
    - Level of interest in using social media
    - Level of importance of social media
    - Belief that social media is harmful to your reputa...
    - Belief that social media is used to waste time
    - Belief that you have the ability to manage social ...
    - Belief that social media promotes social reputatio...
    - Belief that social media is intended for personal ...
    - Belief that social media develops communication sk...
    - Belief that social media is a valuable communicati...
    - Belief that the costs of social media outweigh the...
    - Belief that social media is an effective tool for ...
    - Belief that social media enhances professional dev...
    - Belief that social media promotes the development ...
    - Belief that social media increase stress and anxie...
  - Bar Chart

    - Familiar with Facebook
    - Familiar with Twitter
    - Familiar with Pinterest
    - Familiar with LInkedIn
    - Familiar with Foursquare
    - Familiar with Tumblr
    - Familiar with GooglePlus
    - Familiar with WikiForums
    - Familiar with Yelp
    - Familiar with YouTube
    - Familiar with MySpace
    - Familiar with Blogs
    - Familiar with Social Bookmarking
    - Aggregate of number of social media sites familiar
    - Joined Facebook
    - Joined Twitter
    - Joined Pinterest
    - Joined LinkedIn
    - Joined Foursquare
    - Joined Tumblr
    - Joined Google Plus
    - Joined YouTube
    - Joined MySpace
    - Joined blogs
    - Joined Social Bookmarking
    - Aggregate of number of social media sites joined
    - Posted Facebook
    - Posted Twitter
    - Posted Pinterest
    - Posted LinkedIN
    - Posted Foursquare
    - Posted Tumblr
    - Posted GooglePlus
    - Posted Youtube
    - Posted MySpace
    - Posted Blogs
    - Aggregate of number of social media sites posted
    - Frequency of visits to social media sites
    - Frequency of posting to social media sites
    - Frequency social media is integrated into academic...
    - How many hours per week do you spend on social med...
    - Published original online projects
    - Level of perceived social media expertise
    - Stage in the process of social media practice
    - Understand website design and publishing
    - Level of interest in using social media
    - Level of importance of social media
    - Belief that social media is harmful to your reputa...
    - Belief that social media is used to waste time
    - Belief that you have the ability to manage social ...
    - Belief that social media promotes social reputatio...
    - Belief that social media is intended for personal ...
    - Belief that social media develops communication sk...
    - Belief that social media is a valuable communicati...
    - Belief that the costs of social media outweigh the...
    - Belief that social media is an effective tool for ...
    - Belief that social media enhances professional dev...
    - Belief that social media promotes the development ...
    - Belief that social media increase stress and anxie...
- Log

  - Log
- Correlations

  - Correlations
- Log

  - Log
- Correlations

  - Correlations

- Delete

Log  
Log - Log - June 16, 2016

FREQUENCIES VARIABLES=FamFacebook FamTwitter FamPinterest FamLinkedIn FamFoursquare FamTumblr  
    FamGooglePlus FamWikiForums FamYelp FamYouTube FamMySpace FamBlogs FamiliarSocialBookmarking  
    AggregFamSites JoinedFacebook JoinedTwitter JoinedPinterest JoinedLinkedIN JoinedFoursquare  
    JoinedTumbler JoinedGooglePlus JoinedYoutube JoinedMySpace Joinedblogs JoinedSocialBookmarking  
    AggregJoinedSites PostedFacebook PostedTwitter PostedPinterest PostedLinkedIn PostedFourSquare  
    PostedTumblr PostedGooglePlus PostedYouTube PostedMySpace PostedBlogs AggregPostSites  
    Howfrequentlydoyouvisitsocialmediasites Howfrequentlydoyoupostorshareinformation  
    FrequentlysocialmediaisintegratedinAcademicactivities  
    Onaveragehowmanyhoursperweekdoyouspendusingsocialme  
    Doyouhaveexperiencecreatingandpublishingonlineprojects Selectthelevelthatbestdescribesyou  
    Choosethestagethatbestdescribeswhereyouareintheproces  
    Doyouunderstandthebasicframeworkofwebsitedesigninterms  
    Onascaleof1to5with5beingthehighestlevelofinterest Onascaleof1to5with5beingthehighestlevelofimportan  
    Socialmediaisharmfultoyourprofessionalreputation Socialmediaismostlyusedtowastetime  
    SocialmediaiseffectivebecauseIbelieveIcanimplementits Socialmediapromotessocialreputation  
    Socialmediaismostlyintendedforpersonaluse Socialmediapromotesthedevelopmentofcommunicationskills  
    Socialmediaisavaluableprofessionaltool Socialmediaistoocostlyintermsofrisktoprofessionalrepu  
    Socialmediaisaneffectivetoolforstudentsofallabilities Socialmediaenhancesmyprofessionaldevelopment  
    Socialmediapromotesthedevelopmentofinterpersonalskills Socialmediaincreasesstressandanxiety  
  /BARCHART PERCENT  
  /ORDER=ANALYSIS.

Frequencies

[DataSet1] /Users/jeanettenovakovich/Desktop/Data in Brief/SMSurvey1stIteration.sav

Frequencies

StatisticsStatistics, table, 1 levels of column headers and 2 levels of row headers, table with 61 columns and 4 rows

|  |  |  |  |  |  |  |  |  |  |  |  |  |  |  |  |  |  |  |  |  |  |  |  |  |  |  |  |  |  |  |  |  |  |  |  |  |  |  |  |  |  |  |  |  |  |  |  |  |  |  |  |  |  |  |  |  |  |  |  |  |
| --- | --- | --- | --- | --- | --- | --- | --- | --- | --- | --- | --- | --- | --- | --- | --- | --- | --- | --- | --- | --- | --- | --- | --- | --- | --- | --- | --- | --- | --- | --- | --- | --- | --- | --- | --- | --- | --- | --- | --- | --- | --- | --- | --- | --- | --- | --- | --- | --- | --- | --- | --- | --- | --- | --- | --- | --- | --- | --- | --- | --- |
|  | | Familiar with Facebook | Familiar with Twitter | Familiar with Pinterest | Familiar with LInkedIn | Familiar with Foursquare | Familiar with Tumblr | Familiar with GooglePlus | Familiar with WikiForums | Familiar with Yelp | Familiar with YouTube | Familiar with MySpace | Familiar with Blogs | Familiar with Social Bookmarking | Aggregate of number of social media sites familiar | Joined Facebook | Joined Twitter | Joined Pinterest | Joined LinkedIn | Joined Foursquare | Joined Tumblr | Joined Google Plus | Joined YouTube | Joined MySpace | Joined blogs | Joined Social Bookmarking | Aggregate of number of social media sites joined | Posted Facebook | Posted Twitter | Posted Pinterest | Posted LinkedIN | Posted Foursquare | Posted Tumblr | Posted GooglePlus | Posted Youtube | Posted MySpace | Posted Blogs | Aggregate of number of social media sites posted | Frequency of visits to social media sites | Frequency of posting to social media sites | Frequency social media is integrated into academic activities | How many hours per week do you spend on social media sites | Published original online projects | Level of perceived social media expertise | Stage in the process of social media practice | Understand website design and publishing | Level of interest in using social media | Level of importance of social media | Belief that social media is harmful to your reputation | Belief that social media is used to waste time | Belief that you have the ability to manage social media effectively | Belief that social media promotes social reputation | Belief that social media is intended for personal use | Belief that social media develops communication skills | Belief that social media is a valuable communication skill | Belief that the costs of social media outweigh the benefits to professional reputation | Belief that social media is an effective tool for all students | Belief that social media enhances professional development | Belief that social media promotes the development of interpersonal skills | Belief that social media increase stress and anxiety |
| N | Valid | 27 | 27 | 27 | 27 | 27 | 27 | 27 | 27 | 27 | 27 | 27 | 27 | 27 | 27 | 27 | 27 | 27 | 27 | 27 | 27 | 27 | 27 | 27 | 27 | 27 | 27 | 27 | 27 | 27 | 27 | 27 | 27 | 27 | 27 | 27 | 27 | 27 | 27 | 27 | 27 | 27 | 27 | 27 | 27 | 27 | 26 | 26 | 27 | 27 | 27 | 27 | 27 | 27 | 27 | 27 | 27 | 27 | 27 | 27 |
|  | Missing | 0 | 0 | 0 | 0 | 0 | 0 | 0 | 0 | 0 | 0 | 0 | 0 | 0 | 0 | 0 | 0 | 0 | 0 | 0 | 0 | 0 | 0 | 0 | 0 | 0 | 0 | 0 | 0 | 0 | 0 | 0 | 0 | 0 | 0 | 0 | 0 | 0 | 0 | 0 | 0 | 0 | 0 | 0 | 0 | 0 | 1 | 1 | 0 | 0 | 0 | 0 | 0 | 0 | 0 | 0 | 0 | 0 | 0 | 0 |
|  |  |  |  |  |  |  |  |  |  |  |  |  |  |  |  |  |  |  |  |  |  |  |  |  |  |  |  |  |  |  |  |  |  |  |  |  |  |  |  |  |  |  |  |  |  |  |  |  |  |  |  |  |  |  |  |  |  |  |  |  |

Frequency Table

Familiar with FacebookFamiliar with Facebook, table, 1 levels of column headers and 2 levels of row headers, table with 6 columns and 5 rows

|  |  |  |  |  |  |
| --- | --- | --- | --- | --- | --- |
|  | | Frequency | Percent | Valid Percent | Cumulative Percent |
| Valid | Familiar with Facebook | 23 | 85.2 | 85.2 | 85.2 |
|  | Not Familiar with Facebook | 4 | 14.8 | 14.8 | 100.0 |
|  | Total | 27 | 100.0 | 100.0 |  |
|  |  |  |  |  |  |

Frequency Table

Familiar with TwitterFamiliar with Twitter, table, 1 levels of column headers and 2 levels of row headers, table with 6 columns and 5 rows

|  |  |  |  |  |  |
| --- | --- | --- | --- | --- | --- |
|  | | Frequency | Percent | Valid Percent | Cumulative Percent |
| Valid | Familiar with Twitter | 21 | 77.8 | 77.8 | 77.8 |
|  | Not Familiar with Twitter | 6 | 22.2 | 22.2 | 100.0 |
|  | Total | 27 | 100.0 | 100.0 |  |
|  |  |  |  |  |  |

Frequency Table

Familiar with PinterestFamiliar with Pinterest, table, 1 levels of column headers and 2 levels of row headers, table with 6 columns and 5 rows

|  |  |  |  |  |  |
| --- | --- | --- | --- | --- | --- |
|  | | Frequency | Percent | Valid Percent | Cumulative Percent |
| Valid | Familiar with Pinterest | 16 | 59.3 | 59.3 | 59.3 |
|  | Not Familiar with Pinterest | 11 | 40.7 | 40.7 | 100.0 |
|  | Total | 27 | 100.0 | 100.0 |  |
|  |  |  |  |  |  |

Frequency Table

Familiar with LInkedInFamiliar with LInkedIn, table, 1 levels of column headers and 2 levels of row headers, table with 6 columns and 5 rows

|  |  |  |  |  |  |
| --- | --- | --- | --- | --- | --- |
|  | | Frequency | Percent | Valid Percent | Cumulative Percent |
| Valid | Familiar with LinkedIn | 18 | 66.7 | 66.7 | 66.7 |
|  | Not familiar LinkedIn | 9 | 33.3 | 33.3 | 100.0 |
|  | Total | 27 | 100.0 | 100.0 |  |
|  |  |  |  |  |  |

Frequency Table

Familiar with FoursquareFamiliar with Foursquare, table, 1 levels of column headers and 2 levels of row headers, table with 6 columns and 5 rows

|  |  |  |  |  |  |
| --- | --- | --- | --- | --- | --- |
|  | | Frequency | Percent | Valid Percent | Cumulative Percent |
| Valid | Familiar with Foursquare | 9 | 33.3 | 33.3 | 33.3 |
|  | Not familiar with foursquare | 18 | 66.7 | 66.7 | 100.0 |
|  | Total | 27 | 100.0 | 100.0 |  |
|  |  |  |  |  |  |

Frequency Table

Familiar with TumblrFamiliar with Tumblr, table, 1 levels of column headers and 2 levels of row headers, table with 6 columns and 5 rows

|  |  |  |  |  |  |
| --- | --- | --- | --- | --- | --- |
|  | | Frequency | Percent | Valid Percent | Cumulative Percent |
| Valid | Familiar with Tumblr | 11 | 40.7 | 40.7 | 40.7 |
|  | Not familiar with tumblr | 16 | 59.3 | 59.3 | 100.0 |
|  | Total | 27 | 100.0 | 100.0 |  |
|  |  |  |  |  |  |

Frequency Table

Familiar with GooglePlusFamiliar with GooglePlus, table, 1 levels of column headers and 2 levels of row headers, table with 6 columns and 5 rows

|  |  |  |  |  |  |
| --- | --- | --- | --- | --- | --- |
|  | | Frequency | Percent | Valid Percent | Cumulative Percent |
| Valid | Familiar with Google Plus | 13 | 48.1 | 48.1 | 48.1 |
|  | Not familiar with Google Plus | 14 | 51.9 | 51.9 | 100.0 |
|  | Total | 27 | 100.0 | 100.0 |  |
|  |  |  |  |  |  |

Frequency Table

Familiar with WikiForumsFamiliar with WikiForums, table, 1 levels of column headers and 2 levels of row headers, table with 6 columns and 5 rows

|  |  |  |  |  |  |
| --- | --- | --- | --- | --- | --- |
|  | | Frequency | Percent | Valid Percent | Cumulative Percent |
| Valid | Familiar with wiki | 5 | 18.5 | 18.5 | 18.5 |
|  | Not familiar with wiki | 22 | 81.5 | 81.5 | 100.0 |
|  | Total | 27 | 100.0 | 100.0 |  |
|  |  |  |  |  |  |

Frequency Table

Familiar with YelpFamiliar with Yelp, table, 1 levels of column headers and 2 levels of row headers, table with 6 columns and 5 rows

|  |  |  |  |  |  |
| --- | --- | --- | --- | --- | --- |
|  | | Frequency | Percent | Valid Percent | Cumulative Percent |
| Valid | Familiar with Yelp | 6 | 22.2 | 22.2 | 22.2 |
|  | Not familiar with Yelp | 21 | 77.8 | 77.8 | 100.0 |
|  | Total | 27 | 100.0 | 100.0 |  |
|  |  |  |  |  |  |

Frequency Table

Familiar with YouTubeFamiliar with YouTube, table, 1 levels of column headers and 2 levels of row headers, table with 6 columns and 5 rows

|  |  |  |  |  |  |
| --- | --- | --- | --- | --- | --- |
|  | | Frequency | Percent | Valid Percent | Cumulative Percent |
| Valid | Familiar with YouTube | 26 | 96.3 | 96.3 | 96.3 |
|  | Not familiar with YouTube | 1 | 3.7 | 3.7 | 100.0 |
|  | Total | 27 | 100.0 | 100.0 |  |
|  |  |  |  |  |  |

Frequency Table

Familiar with MySpaceFamiliar with MySpace, table, 1 levels of column headers and 2 levels of row headers, table with 6 columns and 5 rows

|  |  |  |  |  |  |
| --- | --- | --- | --- | --- | --- |
|  | | Frequency | Percent | Valid Percent | Cumulative Percent |
| Valid | Familiar with MySpace | 11 | 40.7 | 40.7 | 40.7 |
|  | Not familiar with MySpace | 16 | 59.3 | 59.3 | 100.0 |
|  | Total | 27 | 100.0 | 100.0 |  |
|  |  |  |  |  |  |

Frequency Table

Familiar with BlogsFamiliar with Blogs, table, 1 levels of column headers and 2 levels of row headers, table with 6 columns and 5 rows

|  |  |  |  |  |  |
| --- | --- | --- | --- | --- | --- |
|  | | Frequency | Percent | Valid Percent | Cumulative Percent |
| Valid | Familiar with blogs | 14 | 51.9 | 51.9 | 51.9 |
|  | Not familiar with blogs | 13 | 48.1 | 48.1 | 100.0 |
|  | Total | 27 | 100.0 | 100.0 |  |
|  |  |  |  |  |  |

Frequency Table

Familiar with Social BookmarkingFamiliar with Social Bookmarking, table, 1 levels of column headers and 2 levels of row headers, table with 6 columns and 5 rows

|  |  |  |  |  |  |
| --- | --- | --- | --- | --- | --- |
|  | | Frequency | Percent | Valid Percent | Cumulative Percent |
| Valid | Familiar with social bookmarking | 9 | 33.3 | 33.3 | 33.3 |
|  | Not familiar with social bookmarking | 18 | 66.7 | 66.7 | 100.0 |
|  | Total | 27 | 100.0 | 100.0 |  |
|  |  |  |  |  |  |

Frequency Table

Aggregate of number of social media sites familiarAggregate of number of social media sites familiar, table, 1 levels of column headers and 2 levels of row headers, table with 6 columns and 14 rows

|  |  |  |  |  |  |
| --- | --- | --- | --- | --- | --- |
|  | | Frequency | Percent | Valid Percent | Cumulative Percent |
| Valid | 1.00 | 1 | 3.7 | 3.7 | 3.7 |
|  | 3.00 | 3 | 11.1 | 11.1 | 14.8 |
|  | 4.00 | 5 | 18.5 | 18.5 | 33.3 |
|  | 5.00 | 4 | 14.8 | 14.8 | 48.1 |
|  | 6.00 | 4 | 14.8 | 14.8 | 63.0 |
|  | 8.00 | 1 | 3.7 | 3.7 | 66.7 |
|  | 9.00 | 1 | 3.7 | 3.7 | 70.4 |
|  | 10.00 | 3 | 11.1 | 11.1 | 81.5 |
|  | 11.00 | 1 | 3.7 | 3.7 | 85.2 |
|  | 12.00 | 3 | 11.1 | 11.1 | 96.3 |
|  | 13.00 | 1 | 3.7 | 3.7 | 100.0 |
|  | Total | 27 | 100.0 | 100.0 |  |
|  |  |  |  |  |  |

Frequency Table

Joined FacebookJoined Facebook, table, 1 levels of column headers and 2 levels of row headers, table with 6 columns and 5 rows

|  |  |  |  |  |  |
| --- | --- | --- | --- | --- | --- |
|  | | Frequency | Percent | Valid Percent | Cumulative Percent |
| Valid | Joined Facebook | 24 | 88.9 | 88.9 | 88.9 |
|  | Not joined Facebook | 3 | 11.1 | 11.1 | 100.0 |
|  | Total | 27 | 100.0 | 100.0 |  |
|  |  |  |  |  |  |

Frequency Table

Joined TwitterJoined Twitter, table, 1 levels of column headers and 2 levels of row headers, table with 6 columns and 5 rows

|  |  |  |  |  |  |
| --- | --- | --- | --- | --- | --- |
|  | | Frequency | Percent | Valid Percent | Cumulative Percent |
| Valid | Joined Twitter | 18 | 66.7 | 66.7 | 66.7 |
|  | Not joined Twitter | 9 | 33.3 | 33.3 | 100.0 |
|  | Total | 27 | 100.0 | 100.0 |  |
|  |  |  |  |  |  |

Frequency Table

Joined PinterestJoined Pinterest, table, 1 levels of column headers and 2 levels of row headers, table with 6 columns and 5 rows

|  |  |  |  |  |  |
| --- | --- | --- | --- | --- | --- |
|  | | Frequency | Percent | Valid Percent | Cumulative Percent |
| Valid | Joined Pinterest | 10 | 37.0 | 37.0 | 37.0 |
|  | Not joined Pinterest | 17 | 63.0 | 63.0 | 100.0 |
|  | Total | 27 | 100.0 | 100.0 |  |
|  |  |  |  |  |  |

Frequency Table

Joined LinkedInJoined LinkedIn, table, 1 levels of column headers and 2 levels of row headers, table with 6 columns and 5 rows

|  |  |  |  |  |  |
| --- | --- | --- | --- | --- | --- |
|  | | Frequency | Percent | Valid Percent | Cumulative Percent |
| Valid | Joined LinkedIn | 15 | 55.6 | 55.6 | 55.6 |
|  | Not joined LinkedIn | 12 | 44.4 | 44.4 | 100.0 |
|  | Total | 27 | 100.0 | 100.0 |  |
|  |  |  |  |  |  |

Frequency Table

Joined FoursquareJoined Foursquare, table, 1 levels of column headers and 2 levels of row headers, table with 6 columns and 5 rows

|  |  |  |  |  |  |
| --- | --- | --- | --- | --- | --- |
|  | | Frequency | Percent | Valid Percent | Cumulative Percent |
| Valid | Joined Foursquare | 6 | 22.2 | 22.2 | 22.2 |
|  | Not joined Foursquare | 21 | 77.8 | 77.8 | 100.0 |
|  | Total | 27 | 100.0 | 100.0 |  |
|  |  |  |  |  |  |

Frequency Table  
Frequency Table - Joined Tumblr - June 16, 2016

Joined TumblrJoined Tumblr, table, 1 levels of column headers and 2 levels of row headers, table with 6 columns and 5 rows

|  |  |  |  |  |  |
| --- | --- | --- | --- | --- | --- |
|  | | Frequency | Percent | Valid Percent | Cumulative Percent |
| Valid | Joined Tumblr | 5 | 18.5 | 18.5 | 18.5 |
|  | Not joined Tumblr | 22 | 81.5 | 81.5 | 100.0 |
|  | Total | 27 | 100.0 | 100.0 |  |
|  |  |  |  |  |  |

Frequency Table  
Frequency Table - Joined Google Plus - June 16, 2016

Joined Google PlusJoined Google Plus, table, 1 levels of column headers and 2 levels of row headers, table with 6 columns and 5 rows

|  |  |  |  |  |  |
| --- | --- | --- | --- | --- | --- |
|  | | Frequency | Percent | Valid Percent | Cumulative Percent |
| Valid | Joined Google Plus | 6 | 22.2 | 22.2 | 22.2 |
|  | Not joined Google Plus | 21 | 77.8 | 77.8 | 100.0 |
|  | Total | 27 | 100.0 | 100.0 |  |
|  |  |  |  |  |  |

Frequency Table  
Frequency Table - Joined YouTube - June 16, 2016

Joined YouTubeJoined YouTube, table, 1 levels of column headers and 2 levels of row headers, table with 6 columns and 5 rows

|  |  |  |  |  |  |
| --- | --- | --- | --- | --- | --- |
|  | | Frequency | Percent | Valid Percent | Cumulative Percent |
| Valid | Joined YouTube | 23 | 85.2 | 85.2 | 85.2 |
|  | Not joined YouTube | 4 | 14.8 | 14.8 | 100.0 |
|  | Total | 27 | 100.0 | 100.0 |  |
|  |  |  |  |  |  |

Frequency Table  
Frequency Table - Joined MySpace - June 16, 2016

Joined MySpaceJoined MySpace, table, 1 levels of column headers and 2 levels of row headers, table with 6 columns and 5 rows

|  |  |  |  |  |  |
| --- | --- | --- | --- | --- | --- |
|  | | Frequency | Percent | Valid Percent | Cumulative Percent |
| Valid | Joined Myspace | 6 | 22.2 | 22.2 | 22.2 |
|  | Not joined Myspace | 21 | 77.8 | 77.8 | 100.0 |
|  | Total | 27 | 100.0 | 100.0 |  |
|  |  |  |  |  |  |

Frequency Table  
Frequency Table - Joined blogs - June 16, 2016

Joined blogsJoined blogs, table, 1 levels of column headers and 2 levels of row headers, table with 6 columns and 5 rows

|  |  |  |  |  |  |
| --- | --- | --- | --- | --- | --- |
|  | | Frequency | Percent | Valid Percent | Cumulative Percent |
| Valid | Joined blogs | 10 | 37.0 | 37.0 | 37.0 |
|  | Not joined blogs | 17 | 63.0 | 63.0 | 100.0 |
|  | Total | 27 | 100.0 | 100.0 |  |
|  |  |  |  |  |  |

Frequency Table  
Frequency Table - Joined Social Bookmarking - June 16, 2016

Joined Social BookmarkingJoined Social Bookmarking, table, 1 levels of column headers and 2 levels of row headers, table with 6 columns and 5 rows

|  |  |  |  |  |  |
| --- | --- | --- | --- | --- | --- |
|  | | Frequency | Percent | Valid Percent | Cumulative Percent |
| Valid | Joined social bookmarking | 1 | 3.7 | 3.7 | 3.7 |
|  | Not joined social bookmarking | 26 | 96.3 | 96.3 | 100.0 |
|  | Total | 27 | 100.0 | 100.0 |  |
|  |  |  |  |  |  |

Frequency Table  
Frequency Table - Aggregate of number of social media sites joined - June 16, 2016

Aggregate of number of social media sites joinedAggregate of number of social media sites joined, table, 1 levels of column headers and 2 levels of row headers, table with 6 columns and 11 rows

|  |  |  |  |  |  |
| --- | --- | --- | --- | --- | --- |
|  | | Frequency | Percent | Valid Percent | Cumulative Percent |
| Valid | 1.00 | 1 | 3.7 | 3.7 | 3.7 |
|  | 3.00 | 10 | 37.0 | 37.0 | 40.7 |
|  | 4.00 | 4 | 14.8 | 14.8 | 55.6 |
|  | 5.00 | 3 | 11.1 | 11.1 | 66.7 |
|  | 6.00 | 5 | 18.5 | 18.5 | 85.2 |
|  | 7.00 | 1 | 3.7 | 3.7 | 88.9 |
|  | 8.00 | 2 | 7.4 | 7.4 | 96.3 |
|  | 9.00 | 1 | 3.7 | 3.7 | 100.0 |
|  | Total | 27 | 100.0 | 100.0 |  |
|  |  |  |  |  |  |

Frequency Table  
Frequency Table - Posted Facebook - June 16, 2016

Posted FacebookPosted Facebook, table, 1 levels of column headers and 2 levels of row headers, table with 6 columns and 5 rows

|  |  |  |  |  |  |
| --- | --- | --- | --- | --- | --- |
|  | | Frequency | Percent | Valid Percent | Cumulative Percent |
| Valid | Posted Facebook | 22 | 81.5 | 81.5 | 81.5 |
|  | Not posted Facebook | 5 | 18.5 | 18.5 | 100.0 |
|  | Total | 27 | 100.0 | 100.0 |  |
|  |  |  |  |  |  |

Frequency Table  
Frequency Table - Posted Twitter - June 16, 2016

Posted TwitterPosted Twitter, table, 1 levels of column headers and 2 levels of row headers, table with 6 columns and 5 rows

|  |  |  |  |  |  |
| --- | --- | --- | --- | --- | --- |
|  | | Frequency | Percent | Valid Percent | Cumulative Percent |
| Valid | Posted Twitter | 19 | 70.4 | 70.4 | 70.4 |
|  | Not posted Twitter | 8 | 29.6 | 29.6 | 100.0 |
|  | Total | 27 | 100.0 | 100.0 |  |
|  |  |  |  |  |  |

Frequency Table  
Frequency Table - Posted Pinterest - June 16, 2016

Posted PinterestPosted Pinterest, table, 1 levels of column headers and 2 levels of row headers, table with 6 columns and 5 rows

|  |  |  |  |  |  |
| --- | --- | --- | --- | --- | --- |
|  | | Frequency | Percent | Valid Percent | Cumulative Percent |
| Valid | Posted Pinterest | 10 | 37.0 | 37.0 | 37.0 |
|  | Not posted Pinterest | 17 | 63.0 | 63.0 | 100.0 |
|  | Total | 27 | 100.0 | 100.0 |  |
|  |  |  |  |  |  |

Frequency Table  
Frequency Table - Posted LinkedIN - June 16, 2016

Posted LinkedINPosted LinkedIN, table, 1 levels of column headers and 2 levels of row headers, table with 6 columns and 5 rows

|  |  |  |  |  |  |
| --- | --- | --- | --- | --- | --- |
|  | | Frequency | Percent | Valid Percent | Cumulative Percent |
| Valid | Posted LinkedIn | 15 | 55.6 | 55.6 | 55.6 |
|  | Not posted LinkedIn | 12 | 44.4 | 44.4 | 100.0 |
|  | Total | 27 | 100.0 | 100.0 |  |
|  |  |  |  |  |  |

Frequency Table  
Frequency Table - Posted Foursquare - June 16, 2016

Posted FoursquarePosted Foursquare, table, 1 levels of column headers and 2 levels of row headers, table with 6 columns and 5 rows

|  |  |  |  |  |  |
| --- | --- | --- | --- | --- | --- |
|  | | Frequency | Percent | Valid Percent | Cumulative Percent |
| Valid | Posted Foursquare | 6 | 22.2 | 22.2 | 22.2 |
|  | Not posted Foursquare | 21 | 77.8 | 77.8 | 100.0 |
|  | Total | 27 | 100.0 | 100.0 |  |
|  |  |  |  |  |  |

Frequency Table  
Frequency Table - Posted Tumblr - June 16, 2016

Posted TumblrPosted Tumblr, table, 1 levels of column headers and 2 levels of row headers, table with 6 columns and 5 rows

|  |  |  |  |  |  |
| --- | --- | --- | --- | --- | --- |
|  | | Frequency | Percent | Valid Percent | Cumulative Percent |
| Valid | Posted Tumblr | 5 | 18.5 | 18.5 | 18.5 |
|  | Not posted Tumblr | 22 | 81.5 | 81.5 | 100.0 |
|  | Total | 27 | 100.0 | 100.0 |  |
|  |  |  |  |  |  |

Frequency Table  
Frequency Table - Posted GooglePlus - June 16, 2016

Posted GooglePlusPosted GooglePlus, table, 1 levels of column headers and 2 levels of row headers, table with 6 columns and 5 rows

|  |  |  |  |  |  |
| --- | --- | --- | --- | --- | --- |
|  | | Frequency | Percent | Valid Percent | Cumulative Percent |
| Valid | Posted Google Plus | 23 | 85.2 | 85.2 | 85.2 |
|  | Not posted Google Plus | 4 | 14.8 | 14.8 | 100.0 |
|  | Total | 27 | 100.0 | 100.0 |  |
|  |  |  |  |  |  |

Frequency Table  
Frequency Table - Posted Youtube - June 16, 2016

Posted YoutubePosted Youtube, table, 1 levels of column headers and 2 levels of row headers, table with 6 columns and 5 rows

|  |  |  |  |  |  |
| --- | --- | --- | --- | --- | --- |
|  | | Frequency | Percent | Valid Percent | Cumulative Percent |
| Valid | Posted YouTube | 12 | 44.4 | 44.4 | 44.4 |
|  | Not posted Youtube | 15 | 55.6 | 55.6 | 100.0 |
|  | Total | 27 | 100.0 | 100.0 |  |
|  |  |  |  |  |  |

Frequency Table  
Frequency Table - Posted MySpace - June 16, 2016

Posted MySpacePosted MySpace, table, 1 levels of column headers and 2 levels of row headers, table with 6 columns and 5 rows

|  |  |  |  |  |  |
| --- | --- | --- | --- | --- | --- |
|  | | Frequency | Percent | Valid Percent | Cumulative Percent |
| Valid | Posted MySpace | 5 | 18.5 | 18.5 | 18.5 |
|  | Not posted MySpace | 22 | 81.5 | 81.5 | 100.0 |
|  | Total | 27 | 100.0 | 100.0 |  |
|  |  |  |  |  |  |

Frequency Table  
Frequency Table - Posted Blogs - June 16, 2016

Posted BlogsPosted Blogs, table, 1 levels of column headers and 2 levels of row headers, table with 6 columns and 5 rows

|  |  |  |  |  |  |
| --- | --- | --- | --- | --- | --- |
|  | | Frequency | Percent | Valid Percent | Cumulative Percent |
| Valid | Posted blogs | 10 | 37.0 | 37.0 | 37.0 |
|  | Not posted blogs | 17 | 63.0 | 63.0 | 100.0 |
|  | Total | 27 | 100.0 | 100.0 |  |
|  |  |  |  |  |  |

Frequency Table  
Frequency Table - Aggregate of number of social media sites posted - June 16, 2016

Aggregate of number of social media sites postedAggregate of number of social media sites posted, table, 1 levels of column headers and 2 levels of row headers, table with 6 columns and 10 rows

|  |  |  |  |  |  |
| --- | --- | --- | --- | --- | --- |
|  | | Frequency | Percent | Valid Percent | Cumulative Percent |
| Valid | 2.00 | 1 | 3.7 | 3.7 | 3.7 |
|  | 3.00 | 6 | 22.2 | 22.2 | 25.9 |
|  | 4.00 | 8 | 29.6 | 29.6 | 55.6 |
|  | 5.00 | 2 | 7.4 | 7.4 | 63.0 |
|  | 6.00 | 6 | 22.2 | 22.2 | 85.2 |
|  | 7.00 | 3 | 11.1 | 11.1 | 96.3 |
|  | 8.00 | 1 | 3.7 | 3.7 | 100.0 |
|  | Total | 27 | 100.0 | 100.0 |  |
|  |  |  |  |  |  |

Frequency Table  
Frequency Table - Frequency of visits to social media sites - June 16, 2016

Frequency of visits to social media sitesFrequency of visits to social media sites, table, 1 levels of column headers and 2 levels of row headers, table with 6 columns and 9 rows

|  |  |  |  |  |  |
| --- | --- | --- | --- | --- | --- |
|  | | Frequency | Percent | Valid Percent | Cumulative Percent |
| Valid | Never | 2 | 7.4 | 7.4 | 7.4 |
|  | Practically never | 2 | 7.4 | 7.4 | 14.8 |
|  | Once in a while | 3 | 11.1 | 11.1 | 25.9 |
|  | Fairly often | 3 | 11.1 | 11.1 | 37.0 |
|  | Very often | 7 | 25.9 | 25.9 | 63.0 |
|  | Almost always | 10 | 37.0 | 37.0 | 100.0 |
|  | Total | 27 | 100.0 | 100.0 |  |
|  |  |  |  |  |  |

Frequency Table  
Frequency Table - Frequency of posting to social media sites - June 16, 2016

Frequency of posting to social media sitesFrequency of posting to social media sites, table, 1 levels of column headers and 2 levels of row headers, table with 6 columns and 9 rows

|  |  |  |  |  |  |
| --- | --- | --- | --- | --- | --- |
|  | | Frequency | Percent | Valid Percent | Cumulative Percent |
| Valid | Never | 2 | 7.4 | 7.4 | 7.4 |
|  | Practically never | 6 | 22.2 | 22.2 | 29.6 |
|  | Once in a while | 5 | 18.5 | 18.5 | 48.1 |
|  | Fairly often | 9 | 33.3 | 33.3 | 81.5 |
|  | Very often | 3 | 11.1 | 11.1 | 92.6 |
|  | Almost always | 2 | 7.4 | 7.4 | 100.0 |
|  | Total | 27 | 100.0 | 100.0 |  |
|  |  |  |  |  |  |

Frequency Table  
Frequency Table - Frequency social media is integrated into academic activities - June 16, 2016

Frequency social media is integrated into academic activitiesFrequency social media is integrated into academic activities, table, 1 levels of column headers and 2 levels of row headers, table with 6 columns and 9 rows

|  |  |  |  |  |  |
| --- | --- | --- | --- | --- | --- |
|  | | Frequency | Percent | Valid Percent | Cumulative Percent |
| Valid | Never | 2 | 7.4 | 7.4 | 7.4 |
|  | Practically never | 4 | 14.8 | 14.8 | 22.2 |
|  | Once in a while | 8 | 29.6 | 29.6 | 51.9 |
|  | Fairly often | 6 | 22.2 | 22.2 | 74.1 |
|  | Very often | 5 | 18.5 | 18.5 | 92.6 |
|  | Almost always | 2 | 7.4 | 7.4 | 100.0 |
|  | Total | 27 | 100.0 | 100.0 |  |
|  |  |  |  |  |  |

Frequency Table  
Frequency Table - How many hours per week do you spend on social media sites - June 16, 2016

How many hours per week do you spend on social media sitesHow many hours per week do you spend on social media sites, table, 1 levels of column headers and 2 levels of row headers, table with 6 columns and 8 rows

|  |  |  |  |  |  |
| --- | --- | --- | --- | --- | --- |
|  | | Frequency | Percent | Valid Percent | Cumulative Percent |
| Valid | None | 4 | 14.8 | 14.8 | 14.8 |
|  | Less than 1 hour | 2 | 7.4 | 7.4 | 22.2 |
|  | 1 to 3 hours | 5 | 18.5 | 18.5 | 40.7 |
|  | 5 t0 10 hours | 10 | 37.0 | 37.0 | 77.8 |
|  | More than 10 hours | 6 | 22.2 | 22.2 | 100.0 |
|  | Total | 27 | 100.0 | 100.0 |  |
|  |  |  |  |  |  |

Frequency Table  
Frequency Table - Published original online projects - June 16, 2016

Published original online projectsPublished original online projects, table, 1 levels of column headers and 2 levels of row headers, table with 6 columns and 6 rows

|  |  |  |  |  |  |
| --- | --- | --- | --- | --- | --- |
|  | | Frequency | Percent | Valid Percent | Cumulative Percent |
| Valid | yes | 14 | 51.9 | 51.9 | 51.9 |
|  | No | 5 | 18.5 | 18.5 | 70.4 |
|  | Uncertain | 8 | 29.6 | 29.6 | 100.0 |
|  | Total | 27 | 100.0 | 100.0 |  |
|  |  |  |  |  |  |

Frequency Table  
Frequency Table - Level of perceived social media expertise - June 16, 2016

Level of perceived social media expertiseLevel of perceived social media expertise, table, 1 levels of column headers and 2 levels of row headers, table with 6 columns and 9 rows

|  |  |  |  |  |  |
| --- | --- | --- | --- | --- | --- |
|  | | Frequency | Percent | Valid Percent | Cumulative Percent |
| Valid | Unfamiliar with no experience | 1 | 3.7 | 3.7 | 3.7 |
|  | Newcomer | 6 | 22.2 | 22.2 | 25.9 |
|  | Beginner | 3 | 11.1 | 11.1 | 37.0 |
|  | Average | 8 | 29.6 | 29.6 | 66.7 |
|  | Advanced | 8 | 29.6 | 29.6 | 96.3 |
|  | Expert | 1 | 3.7 | 3.7 | 100.0 |
|  | Total | 27 | 100.0 | 100.0 |  |
|  |  |  |  |  |  |

Frequency Table  
Frequency Table - Stage in the process of social media practice - June 16, 2016

Stage in the process of social media practiceStage in the process of social media practice, table, 1 levels of column headers and 2 levels of row headers, table with 6 columns and 9 rows

|  |  |  |  |  |  |
| --- | --- | --- | --- | --- | --- |
|  | | Frequency | Percent | Valid Percent | Cumulative Percent |
| Valid | Awareness | 3 | 11.1 | 11.1 | 11.1 |
|  | Learning | 5 | 18.5 | 18.5 | 29.6 |
|  | Understanding | 2 | 7.4 | 7.4 | 37.0 |
|  | Familiarity | 9 | 33.3 | 33.3 | 70.4 |
|  | Adaptation | 5 | 18.5 | 18.5 | 88.9 |
|  | Creative Application | 3 | 11.1 | 11.1 | 100.0 |
|  | Total | 27 | 100.0 | 100.0 |  |
|  |  |  |  |  |  |

Frequency Table  
Frequency Table - Understand website design and publishing - June 16, 2016

Understand website design and publishingUnderstand website design and publishing, table, 1 levels of column headers and 2 levels of row headers, table with 6 columns and 6 rows

|  |  |  |  |  |  |
| --- | --- | --- | --- | --- | --- |
|  | | Frequency | Percent | Valid Percent | Cumulative Percent |
| Valid | Yes | 12 | 44.4 | 44.4 | 44.4 |
|  | No | 10 | 37.0 | 37.0 | 81.5 |
|  | Uncertain | 5 | 18.5 | 18.5 | 100.0 |
|  | Total | 27 | 100.0 | 100.0 |  |
|  |  |  |  |  |  |

Frequency Table  
Frequency Table - Level of interest in using social media - June 16, 2016

Level of interest in using social mediaLevel of interest in using social media, table, 1 levels of column headers and 2 levels of row headers, table with 6 columns and 10 rows

|  |  |  |  |  |  |
| --- | --- | --- | --- | --- | --- |
|  | | Frequency | Percent | Valid Percent | Cumulative Percent |
| Valid | Strongly disagree | 2 | 7.4 | 7.7 | 7.7 |
|  | Disagree | 2 | 7.4 | 7.7 | 15.4 |
|  | No opinion | 7 | 25.9 | 26.9 | 42.3 |
|  | Agree | 5 | 18.5 | 19.2 | 61.5 |
|  | Strongly agree | 10 | 37.0 | 38.5 | 100.0 |
|  | Total | 26 | 96.3 | 100.0 |  |
| Missing | System | 1 | 3.7 |  |  |
| Total | | 27 | 100.0 |  |  |
|  |  |  |  |  |  |

Frequency Table  
Frequency Table - Level of importance of social media - June 16, 2016

Level of importance of social mediaLevel of importance of social media, table, 1 levels of column headers and 2 levels of row headers, table with 6 columns and 9 rows

|  |  |  |  |  |  |
| --- | --- | --- | --- | --- | --- |
|  | | Frequency | Percent | Valid Percent | Cumulative Percent |
| Valid | Disagree | 1 | 3.7 | 3.8 | 3.8 |
|  | No opinion | 6 | 22.2 | 23.1 | 26.9 |
|  | Agree | 5 | 18.5 | 19.2 | 46.2 |
|  | Strongly agree | 14 | 51.9 | 53.8 | 100.0 |
|  | Total | 26 | 96.3 | 100.0 |  |
| Missing | System | 1 | 3.7 |  |  |
| Total | | 27 | 100.0 |  |  |
|  |  |  |  |  |  |

Frequency Table  
Frequency Table - Belief that social media is harmful to your reputation - June 16, 2016

Belief that social media is harmful to your reputationBelief that social media is harmful to your reputation, table, 1 levels of column headers and 2 levels of row headers, table with 6 columns and 7 rows

|  |  |  |  |  |  |
| --- | --- | --- | --- | --- | --- |
|  | | Frequency | Percent | Valid Percent | Cumulative Percent |
| Valid | Strongly disagree | 2 | 7.4 | 7.4 | 7.4 |
|  | Disagree | 13 | 48.1 | 48.1 | 55.6 |
|  | No opinion | 10 | 37.0 | 37.0 | 92.6 |
|  | Agree | 2 | 7.4 | 7.4 | 100.0 |
|  | Total | 27 | 100.0 | 100.0 |  |
|  |  |  |  |  |  |

Frequency Table  
Frequency Table - Belief that social media is used to waste time - June 16, 2016

Belief that social media is used to waste timeBelief that social media is used to waste time, table, 1 levels of column headers and 2 levels of row headers, table with 6 columns and 8 rows

|  |  |  |  |  |  |
| --- | --- | --- | --- | --- | --- |
|  | | Frequency | Percent | Valid Percent | Cumulative Percent |
| Valid | Strongly disagree | 2 | 7.4 | 7.4 | 7.4 |
|  | Disagree | 13 | 48.1 | 48.1 | 55.6 |
|  | No opinion | 3 | 11.1 | 11.1 | 66.7 |
|  | Agree | 7 | 25.9 | 25.9 | 92.6 |
|  | Strongly agree | 2 | 7.4 | 7.4 | 100.0 |
|  | Total | 27 | 100.0 | 100.0 |  |
|  |  |  |  |  |  |

Frequency Table  
Frequency Table - Belief that you have the ability to manage social media effectively - June 16, 201(more)6(less)

Belief that you have the ability to manage social media effectivelyBelief that you have the ability to manage social media effectively, table, 1 levels of column headers and 2 levels of row headers, table with 6 columns and 8 rows

|  |  |  |  |  |  |
| --- | --- | --- | --- | --- | --- |
|  | | Frequency | Percent | Valid Percent | Cumulative Percent |
| Valid | Strongly disagree | 4 | 14.8 | 14.8 | 14.8 |
|  | Disagree | 2 | 7.4 | 7.4 | 22.2 |
|  | No opinion | 6 | 22.2 | 22.2 | 44.4 |
|  | Agree | 10 | 37.0 | 37.0 | 81.5 |
|  | Strongly agree | 5 | 18.5 | 18.5 | 100.0 |
|  | Total | 27 | 100.0 | 100.0 |  |
|  |  |  |  |  |  |

Frequency Table  
Frequency Table - Belief that social media promotes social reputation - June 16, 2016

Belief that social media promotes social reputationBelief that social media promotes social reputation, table, 1 levels of column headers and 2 levels of row headers, table with 6 columns and 7 rows

|  |  |  |  |  |  |
| --- | --- | --- | --- | --- | --- |
|  | | Frequency | Percent | Valid Percent | Cumulative Percent |
| Valid | Disagree | 2 | 7.4 | 7.4 | 7.4 |
|  | No opinion | 7 | 25.9 | 25.9 | 33.3 |
|  | Agree | 10 | 37.0 | 37.0 | 70.4 |
|  | Strongly agree | 8 | 29.6 | 29.6 | 100.0 |
|  | Total | 27 | 100.0 | 100.0 |  |
|  |  |  |  |  |  |

Frequency Table  
Frequency Table - Belief that social media is intended for personal use - June 16, 2016

Belief that social media is intended for personal useBelief that social media is intended for personal use, table, 1 levels of column headers and 2 levels of row headers, table with 6 columns and 8 rows

|  |  |  |  |  |  |
| --- | --- | --- | --- | --- | --- |
|  | | Frequency | Percent | Valid Percent | Cumulative Percent |
| Valid | Strongly disagree | 1 | 3.7 | 3.7 | 3.7 |
|  | Disagree | 10 | 37.0 | 37.0 | 40.7 |
|  | No opinoon | 8 | 29.6 | 29.6 | 70.4 |
|  | Agree | 6 | 22.2 | 22.2 | 92.6 |
|  | Strongly agree | 2 | 7.4 | 7.4 | 100.0 |
|  | Total | 27 | 100.0 | 100.0 |  |
|  |  |  |  |  |  |

Frequency Table  
Frequency Table - Belief that social media develops communication skills - June 16, 2016

Belief that social media develops communication skillsBelief that social media develops communication skills, table, 1 levels of column headers and 2 levels of row headers, table with 6 columns and 8 rows

|  |  |  |  |  |  |
| --- | --- | --- | --- | --- | --- |
|  | | Frequency | Percent | Valid Percent | Cumulative Percent |
| Valid | Strongly disagree | 2 | 7.4 | 7.4 | 7.4 |
|  | Disagree | 3 | 11.1 | 11.1 | 18.5 |
|  | No opinion | 4 | 14.8 | 14.8 | 33.3 |
|  | Agree | 13 | 48.1 | 48.1 | 81.5 |
|  | Strongly agree | 5 | 18.5 | 18.5 | 100.0 |
|  | Total | 27 | 100.0 | 100.0 |  |
|  |  |  |  |  |  |

Frequency Table  
Frequency Table - Belief that social media is a valuable communication skill - June 16, 2016

Belief that social media is a valuable communication skillBelief that social media is a valuable communication skill, table, 1 levels of column headers and 2 levels of row headers, table with 6 columns and 6 rows

|  |  |  |  |  |  |
| --- | --- | --- | --- | --- | --- |
|  | | Frequency | Percent | Valid Percent | Cumulative Percent |
| Valid | No opinion | 7 | 25.9 | 25.9 | 25.9 |
|  | Agree | 14 | 51.9 | 51.9 | 77.8 |
|  | Strongly agree | 6 | 22.2 | 22.2 | 100.0 |
|  | Total | 27 | 100.0 | 100.0 |  |
|  |  |  |  |  |  |

Frequency Table  
Frequency Table - Belief that the costs of social media outweigh the benefits to professional reputa(more)tion - June 16, 2016(less)

Belief that the costs of social media outweigh the benefits to professional reputationBelief that the costs of social media outweigh the benefits to professional reputation, table, 1 levels of column headers and 2 levels of row headers, table with 6 columns and 8 rows

|  |  |  |  |  |  |
| --- | --- | --- | --- | --- | --- |
|  | | Frequency | Percent | Valid Percent | Cumulative Percent |
| Valid | Strongly disagree | 3 | 11.1 | 11.1 | 11.1 |
|  | Disagree | 10 | 37.0 | 37.0 | 48.1 |
|  | No opinion | 7 | 25.9 | 25.9 | 74.1 |
|  | Agree | 5 | 18.5 | 18.5 | 92.6 |
|  | Strongly agree | 2 | 7.4 | 7.4 | 100.0 |
|  | Total | 27 | 100.0 | 100.0 |  |
|  |  |  |  |  |  |

Frequency Table  
Frequency Table - Belief that social media is an effective tool for all students - June 16, 2016

Belief that social media is an effective tool for all studentsBelief that social media is an effective tool for all students, table, 1 levels of column headers and 2 levels of row headers, table with 6 columns and 8 rows

|  |  |  |  |  |  |
| --- | --- | --- | --- | --- | --- |
|  | | Frequency | Percent | Valid Percent | Cumulative Percent |
| Valid | Strongly disagree | 1 | 3.7 | 3.7 | 3.7 |
|  | Disagree | 4 | 14.8 | 14.8 | 18.5 |
|  | No opinion | 8 | 29.6 | 29.6 | 48.1 |
|  | Agree | 9 | 33.3 | 33.3 | 81.5 |
|  | Strongly agree | 5 | 18.5 | 18.5 | 100.0 |
|  | Total | 27 | 100.0 | 100.0 |  |
|  |  |  |  |  |  |

Frequency Table  
Frequency Table - Belief that social media enhances professional development - June 16, 2016

Belief that social media enhances professional developmentBelief that social media enhances professional development, table, 1 levels of column headers and 2 levels of row headers, table with 6 columns and 8 rows

|  |  |  |  |  |  |
| --- | --- | --- | --- | --- | --- |
|  | | Frequency | Percent | Valid Percent | Cumulative Percent |
| Valid | Strongly disagree | 1 | 3.7 | 3.7 | 3.7 |
|  | Disagree | 2 | 7.4 | 7.4 | 11.1 |
|  | No opinion | 10 | 37.0 | 37.0 | 48.1 |
|  | Agree | 8 | 29.6 | 29.6 | 77.8 |
|  | Strongly agree | 6 | 22.2 | 22.2 | 100.0 |
|  | Total | 27 | 100.0 | 100.0 |  |
|  |  |  |  |  |  |

Frequency Table  
Frequency Table - Belief that social media promotes the development of interpersonal skills - June 1(more)6, 2016(less)

Belief that social media promotes the development of interpersonal skillsBelief that social media promotes the development of interpersonal skills, table, 1 levels of column headers and 2 levels of row headers, table with 6 columns and 8 rows

|  |  |  |  |  |  |
| --- | --- | --- | --- | --- | --- |
|  | | Frequency | Percent | Valid Percent | Cumulative Percent |
| Valid | Strongly disagree | 2 | 7.4 | 7.4 | 7.4 |
|  | Disagree | 2 | 7.4 | 7.4 | 14.8 |
|  | No opinion | 13 | 48.1 | 48.1 | 63.0 |
|  | Agree | 7 | 25.9 | 25.9 | 88.9 |
|  | Strongly agree | 3 | 11.1 | 11.1 | 100.0 |
|  | Total | 27 | 100.0 | 100.0 |  |
|  |  |  |  |  |  |

Frequency Table  
Frequency Table - Belief that social media increase stress and anxiety - June 16, 2016

Belief that social media increase stress and anxietyBelief that social media increase stress and anxiety, table, 1 levels of column headers and 2 levels of row headers, table with 6 columns and 7 rows

|  |  |  |  |  |  |
| --- | --- | --- | --- | --- | --- |
|  | | Frequency | Percent | Valid Percent | Cumulative Percent |
| Valid | Disagree | 6 | 22.2 | 22.2 | 22.2 |
|  | No opinion | 13 | 48.1 | 48.1 | 70.4 |
|  | Agree | 6 | 22.2 | 22.2 | 92.6 |
|  | Strongly agree | 2 | 7.4 | 7.4 | 100.0 |
|  | Total | 27 | 100.0 | 100.0 |  |
|  |  |  |  |  |  |

Bar Chart  
Bar Chart - Familiar with Facebook - June 16, 2016

{"style":{"fill":{"g":255,"b":255,"r":255},"outline":{"g":0,"b":0,"r":0,"a":0.0}},"data":[{"id":"dSource","rows":[[0,85.18518518518519],[1,14.81481481481481]],"fields":[{"id":"fVariable","categories":["Familiar with Facebook","Not Familiar with Facebook"],"label":"Familiar with Facebook","format":{"numericPattern":"###"}},{"min":14.81481481481481,"id":"fVariable1","max":85.18518518518519,"label":"Y Axis"}]}],"titles":[{"backgroundStyle":{"fill":{"g":0,"b":0,"r":0,"a":0.0},"outline":{"g":0,"b":0,"r":0,"a":0.0}},"content":["Familiar with Facebook"],"style":{"font":{"weight":"bold","family":"'Helvetica Neue;Helvetica;Arial;SansSerif', sans-serif","size":"12pt"},"fill":{"g":100,"b":157,"r":0},"padding":3.0},"type":"title"}],"copyright":"(C) Copyright IBM Corp. 2011","version":"6.0","size":{"height":500.0,"width":625.0},"grammar":[{"elements":[{"position":[{"field":{"$ref":"fVariable1"}},{"field":{"$ref":"fVariable"}}],"style":{"fill":{"g":178,"b":239,"r":0},"outline":{"g":100,"b":157,"r":0},"size":"75.0%"},"data":{"$ref":"dSource"},"type":"interval"}],"coordinates":{"style":{"fill":{"g":255,"b":255,"r":255},"outline":{"g":100,"b":157,"r":0}},"dimensions":[{"axis":[{"title":["Percent"],"lineStyle":{"stroke":{"width":0.6666667},"fill":{"g":100,"b":157,"r":0}},"tickStyle":{"font":{"weight":"normal","family":"sans-serif","size":"8pt"},"fill":{"g":100,"b":157,"r":0}},"titleStyle":{"font":{"weight":"bold","family":"'Helvetica Neue;Helvetica;Arial;SansSerif', sans-serif","size":"11pt"},"fill":{"g":100,"b":157,"r":0},"padding":6.0},"markStyle":{"stroke":{"width":1.3333334},"fill":{"g":100,"b":157,"r":0}}}],"scale":{"spans":[{"tickDelta":20.0,"outRange":{"min":0.0,"max":1.0}}],"padding":{"right":"5%"}}},{"axis":[{"title":["Familiar with Facebook"],"lineStyle":{"stroke":{"width":0.6666667},"fill":{"g":100,"b":157,"r":0}},"tickStyle":{"font":{"weight":"normal","family":"sans-serif","size":"8pt"},"fill":{"g":100,"b":157,"r":0}},"titleStyle":{"font":{"weight":"bold","family":"'Helvetica Neue;Helvetica;Arial;SansSerif', sans-serif","size":"11pt"},"fill":{"g":100,"b":157,"r":0},"padding":6.0},"markStyle":{"stroke":{"width":1.3333334},"fill":{"g":100,"b":157,"r":0}}}],"scale":{"spans":[{"tickDelta":1.0}],"padding":{"left":"5%","right":"5%"}}}]}}]}

Bar Chart  
Bar Chart - Familiar with Twitter - June 16, 2016

{"style":{"fill":{"g":255,"b":255,"r":255},"outline":{"g":0,"b":0,"r":0,"a":0.0}},"data":[{"id":"dSource","rows":[[0,77.77777777777779],[1,22.22222222222222]],"fields":[{"id":"fVariable","categories":["Familiar with Twitter","Not Familiar with Twitter"],"label":"Familiar with Twitter","format":{"numericPattern":"###"}},{"min":22.22222222222222,"id":"fVariable1","max":77.77777777777779,"label":"Y Axis"}]}],"titles":[{"backgroundStyle":{"fill":{"g":0,"b":0,"r":0,"a":0.0},"outline":{"g":0,"b":0,"r":0,"a":0.0}},"content":["Familiar with Twitter"],"style":{"font":{"weight":"bold","family":"'Helvetica Neue;Helvetica;Arial;SansSerif', sans-serif","size":"12pt"},"fill":{"g":100,"b":157,"r":0},"padding":3.0},"type":"title"}],"copyright":"(C) Copyright IBM Corp. 2011","version":"6.0","size":{"height":500.0,"width":625.0},"grammar":[{"elements":[{"position":[{"field":{"$ref":"fVariable1"}},{"field":{"$ref":"fVariable"}}],"style":{"fill":{"g":178,"b":239,"r":0},"outline":{"g":100,"b":157,"r":0},"size":"75.0%"},"data":{"$ref":"dSource"},"type":"interval"}],"coordinates":{"style":{"fill":{"g":255,"b":255,"r":255},"outline":{"g":100,"b":157,"r":0}},"dimensions":[{"axis":[{"title":["Percent"],"lineStyle":{"stroke":{"width":0.6666667},"fill":{"g":100,"b":157,"r":0}},"tickStyle":{"font":{"weight":"normal","family":"sans-serif","size":"8pt"},"fill":{"g":100,"b":157,"r":0}},"titleStyle":{"font":{"weight":"bold","family":"'Helvetica Neue;Helvetica;Arial;SansSerif', sans-serif","size":"11pt"},"fill":{"g":100,"b":157,"r":0},"padding":6.0},"markStyle":{"stroke":{"width":1.3333334},"fill":{"g":100,"b":157,"r":0}}}],"scale":{"spans":[{"tickDelta":20.0,"outRange":{"min":0.0,"max":1.0}}],"padding":{"right":"5%"}}},{"axis":[{"title":["Familiar with Twitter"],"lineStyle":{"stroke":{"width":0.6666667},"fill":{"g":100,"b":157,"r":0}},"tickStyle":{"font":{"weight":"normal","family":"sans-serif","size":"8pt"},"fill":{"g":100,"b":157,"r":0}},"titleStyle":{"font":{"weight":"bold","family":"'Helvetica Neue;Helvetica;Arial;SansSerif', sans-serif","size":"11pt"},"fill":{"g":100,"b":157,"r":0},"padding":6.0},"markStyle":{"stroke":{"width":1.3333334},"fill":{"g":100,"b":157,"r":0}}}],"scale":{"spans":[{"tickDelta":1.0}],"padding":{"left":"5%","right":"5%"}}}]}}]}

Bar Chart  
Bar Chart - Familiar with Pinterest - June 16, 2016

{"style":{"fill":{"g":255,"b":255,"r":255},"outline":{"g":0,"b":0,"r":0,"a":0.0}},"data":[{"id":"dSource","rows":[[0,59.25925925925925],[1,40.74074074074074]],"fields":[{"id":"fVariable","categories":["Familiar with Pinterest","Not Familiar with Pinterest"],"label":"Familiar with Pinterest","format":{"numericPattern":"###"}},{"min":40.74074074074074,"id":"fVariable1","max":59.25925925925925,"label":"Y Axis"}]}],"titles":[{"backgroundStyle":{"fill":{"g":0,"b":0,"r":0,"a":0.0},"outline":{"g":0,"b":0,"r":0,"a":0.0}},"content":["Familiar with Pinterest"],"style":{"font":{"weight":"bold","family":"'Helvetica Neue;Helvetica;Arial;SansSerif', sans-serif","size":"12pt"},"fill":{"g":100,"b":157,"r":0},"padding":3.0},"type":"title"}],"copyright":"(C) Copyright IBM Corp. 2011","version":"6.0","size":{"height":500.0,"width":625.0},"grammar":[{"elements":[{"position":[{"field":{"$ref":"fVariable1"}},{"field":{"$ref":"fVariable"}}],"style":{"fill":{"g":178,"b":239,"r":0},"outline":{"g":100,"b":157,"r":0},"size":"75.0%"},"data":{"$ref":"dSource"},"type":"interval"}],"coordinates":{"style":{"fill":{"g":255,"b":255,"r":255},"outline":{"g":100,"b":157,"r":0}},"dimensions":[{"axis":[{"title":["Percent"],"lineStyle":{"stroke":{"width":0.6666667},"fill":{"g":100,"b":157,"r":0}},"tickStyle":{"font":{"weight":"normal","family":"sans-serif","size":"8pt"},"fill":{"g":100,"b":157,"r":0}},"titleStyle":{"font":{"weight":"bold","family":"'Helvetica Neue;Helvetica;Arial;SansSerif', sans-serif","size":"11pt"},"fill":{"g":100,"b":157,"r":0},"padding":6.0},"markStyle":{"stroke":{"width":1.3333334},"fill":{"g":100,"b":157,"r":0}}}],"scale":{"spans":[{"tickDelta":10.0,"outRange":{"min":0.0,"max":1.0}}],"padding":{"right":"5%"}}},{"axis":[{"title":["Familiar with Pinterest"],"lineStyle":{"stroke":{"width":0.6666667},"fill":{"g":100,"b":157,"r":0}},"tickStyle":{"font":{"weight":"normal","family":"sans-serif","size":"8pt"},"fill":{"g":100,"b":157,"r":0}},"titleStyle":{"font":{"weight":"bold","family":"'Helvetica Neue;Helvetica;Arial;SansSerif', sans-serif","size":"11pt"},"fill":{"g":100,"b":157,"r":0},"padding":6.0},"markStyle":{"stroke":{"width":1.3333334},"fill":{"g":100,"b":157,"r":0}}}],"scale":{"spans":[{"tickDelta":1.0}],"padding":{"left":"5%","right":"5%"}}}]}}]}

Bar Chart  
Bar Chart - Familiar with LInkedIn - June 16, 2016

{"style":{"fill":{"g":255,"b":255,"r":255},"outline":{"g":0,"b":0,"r":0,"a":0.0}},"data":[{"id":"dSource","rows":[[0,66.66666666666666],[1,33.33333333333333]],"fields":[{"id":"fVariable","categories":["Familiar with LinkedIn","Not familiar LinkedIn"],"label":"Familiar with LInkedIn","format":{"numericPattern":"###"}},{"min":33.33333333333333,"id":"fVariable1","max":66.66666666666666,"label":"Y Axis"}]}],"titles":[{"backgroundStyle":{"fill":{"g":0,"b":0,"r":0,"a":0.0},"outline":{"g":0,"b":0,"r":0,"a":0.0}},"content":["Familiar with LInkedIn"],"style":{"font":{"weight":"bold","family":"'Helvetica Neue;Helvetica;Arial;SansSerif', sans-serif","size":"12pt"},"fill":{"g":100,"b":157,"r":0},"padding":3.0},"type":"title"}],"copyright":"(C) Copyright IBM Corp. 2011","version":"6.0","size":{"height":500.0,"width":625.0},"grammar":[{"elements":[{"position":[{"field":{"$ref":"fVariable1"}},{"field":{"$ref":"fVariable"}}],"style":{"fill":{"g":178,"b":239,"r":0},"outline":{"g":100,"b":157,"r":0},"size":"75.0%"},"data":{"$ref":"dSource"},"type":"interval"}],"coordinates":{"style":{"fill":{"g":255,"b":255,"r":255},"outline":{"g":100,"b":157,"r":0}},"dimensions":[{"axis":[{"title":["Percent"],"lineStyle":{"stroke":{"width":0.6666667},"fill":{"g":100,"b":157,"r":0}},"tickStyle":{"font":{"weight":"normal","family":"sans-serif","size":"8pt"},"fill":{"g":100,"b":157,"r":0}},"titleStyle":{"font":{"weight":"bold","family":"'Helvetica Neue;Helvetica;Arial;SansSerif', sans-serif","size":"11pt"},"fill":{"g":100,"b":157,"r":0},"padding":6.0},"markStyle":{"stroke":{"width":1.3333334},"fill":{"g":100,"b":157,"r":0}}}],"scale":{"spans":[{"tickDelta":20.0,"outRange":{"min":0.0,"max":1.0}}],"padding":{"right":"5%"}}},{"axis":[{"title":["Familiar with LInkedIn"],"lineStyle":{"stroke":{"width":0.6666667},"fill":{"g":100,"b":157,"r":0}},"tickStyle":{"font":{"weight":"normal","family":"sans-serif","size":"8pt"},"fill":{"g":100,"b":157,"r":0}},"titleStyle":{"font":{"weight":"bold","family":"'Helvetica Neue;Helvetica;Arial;SansSerif', sans-serif","size":"11pt"},"fill":{"g":100,"b":157,"r":0},"padding":6.0},"markStyle":{"stroke":{"width":1.3333334},"fill":{"g":100,"b":157,"r":0}}}],"scale":{"spans":[{"tickDelta":1.0}],"padding":{"left":"5%","right":"5%"}}}]}}]}

Bar Chart  
Bar Chart - Familiar with Foursquare - June 16, 2016

{"style":{"fill":{"g":255,"b":255,"r":255},"outline":{"g":0,"b":0,"r":0,"a":0.0}},"data":[{"id":"dSource","rows":[[0,33.33333333333333],[1,66.66666666666666]],"fields":[{"id":"fVariable","categories":["Familiar with Foursquare","Not familiar with foursquare"],"label":"Familiar with Foursquare","format":{"numericPattern":"###"}},{"min":33.33333333333333,"id":"fVariable1","max":66.66666666666666,"label":"Y Axis"}]}],"titles":[{"backgroundStyle":{"fill":{"g":0,"b":0,"r":0,"a":0.0},"outline":{"g":0,"b":0,"r":0,"a":0.0}},"content":["Familiar with Foursquare"],"style":{"font":{"weight":"bold","family":"'Helvetica Neue;Helvetica;Arial;SansSerif', sans-serif","size":"12pt"},"fill":{"g":100,"b":157,"r":0},"padding":3.0},"type":"title"}],"copyright":"(C) Copyright IBM Corp. 2011","version":"6.0","size":{"height":500.0,"width":625.0},"grammar":[{"elements":[{"position":[{"field":{"$ref":"fVariable1"}},{"field":{"$ref":"fVariable"}}],"style":{"fill":{"g":178,"b":239,"r":0},"outline":{"g":100,"b":157,"r":0},"size":"75.0%"},"data":{"$ref":"dSource"},"type":"interval"}],"coordinates":{"style":{"fill":{"g":255,"b":255,"r":255},"outline":{"g":100,"b":157,"r":0}},"dimensions":[{"axis":[{"title":["Percent"],"lineStyle":{"stroke":{"width":0.6666667},"fill":{"g":100,"b":157,"r":0}},"tickStyle":{"font":{"weight":"normal","family":"sans-serif","size":"8pt"},"fill":{"g":100,"b":157,"r":0}},"titleStyle":{"font":{"weight":"bold","family":"'Helvetica Neue;Helvetica;Arial;SansSerif', sans-serif","size":"11pt"},"fill":{"g":100,"b":157,"r":0},"padding":6.0},"markStyle":{"stroke":{"width":1.3333334},"fill":{"g":100,"b":157,"r":0}}}],"scale":{"spans":[{"tickDelta":20.0,"outRange":{"min":0.0,"max":1.0}}],"padding":{"right":"5%"}}},{"axis":[{"title":["Familiar with Foursquare"],"lineStyle":{"stroke":{"width":0.6666667},"fill":{"g":100,"b":157,"r":0}},"tickStyle":{"font":{"weight":"normal","family":"sans-serif","size":"8pt"},"fill":{"g":100,"b":157,"r":0}},"titleStyle":{"font":{"weight":"bold","family":"'Helvetica Neue;Helvetica;Arial;SansSerif', sans-serif","size":"11pt"},"fill":{"g":100,"b":157,"r":0},"padding":6.0},"markStyle":{"stroke":{"width":1.3333334},"fill":{"g":100,"b":157,"r":0}}}],"scale":{"spans":[{"tickDelta":1.0}],"padding":{"left":"5%","right":"5%"}}}]}}]}

Bar Chart  
Bar Chart - Familiar with Tumblr - June 16, 2016

{"style":{"fill":{"g":255,"b":255,"r":255},"outline":{"g":0,"b":0,"r":0,"a":0.0}},"data":[{"id":"dSource","rows":[[0,40.74074074074074],[1,59.25925925925925]],"fields":[{"id":"fVariable","categories":["Familiar with Tumblr","Not familiar with tumblr"],"label":"Familiar with Tumblr","format":{"numericPattern":"###"}},{"min":40.74074074074074,"id":"fVariable1","max":59.25925925925925,"label":"Y Axis"}]}],"titles":[{"backgroundStyle":{"fill":{"g":0,"b":0,"r":0,"a":0.0},"outline":{"g":0,"b":0,"r":0,"a":0.0}},"content":["Familiar with Tumblr"],"style":{"font":{"weight":"bold","family":"'Helvetica Neue;Helvetica;Arial;SansSerif', sans-serif","size":"12pt"},"fill":{"g":100,"b":157,"r":0},"padding":3.0},"type":"title"}],"copyright":"(C) Copyright IBM Corp. 2011","version":"6.0","size":{"height":500.0,"width":625.0},"grammar":[{"elements":[{"position":[{"field":{"$ref":"fVariable1"}},{"field":{"$ref":"fVariable"}}],"style":{"fill":{"g":178,"b":239,"r":0},"outline":{"g":100,"b":157,"r":0},"size":"75.0%"},"data":{"$ref":"dSource"},"type":"interval"}],"coordinates":{"style":{"fill":{"g":255,"b":255,"r":255},"outline":{"g":100,"b":157,"r":0}},"dimensions":[{"axis":[{"title":["Percent"],"lineStyle":{"stroke":{"width":0.6666667},"fill":{"g":100,"b":157,"r":0}},"tickStyle":{"font":{"weight":"normal","family":"sans-serif","size":"8pt"},"fill":{"g":100,"b":157,"r":0}},"titleStyle":{"font":{"weight":"bold","family":"'Helvetica Neue;Helvetica;Arial;SansSerif', sans-serif","size":"11pt"},"fill":{"g":100,"b":157,"r":0},"padding":6.0},"markStyle":{"stroke":{"width":1.3333334},"fill":{"g":100,"b":157,"r":0}}}],"scale":{"spans":[{"tickDelta":10.0,"outRange":{"min":0.0,"max":1.0}}],"padding":{"right":"5%"}}},{"axis":[{"title":["Familiar with Tumblr"],"lineStyle":{"stroke":{"width":0.6666667},"fill":{"g":100,"b":157,"r":0}},"tickStyle":{"font":{"weight":"normal","family":"sans-serif","size":"8pt"},"fill":{"g":100,"b":157,"r":0}},"titleStyle":{"font":{"weight":"bold","family":"'Helvetica Neue;Helvetica;Arial;SansSerif', sans-serif","size":"11pt"},"fill":{"g":100,"b":157,"r":0},"padding":6.0},"markStyle":{"stroke":{"width":1.3333334},"fill":{"g":100,"b":157,"r":0}}}],"scale":{"spans":[{"tickDelta":1.0}],"padding":{"left":"5%","right":"5%"}}}]}}]}

Bar Chart  
Bar Chart - Familiar with GooglePlus - June 16, 2016

{"style":{"fill":{"g":255,"b":255,"r":255},"outline":{"g":0,"b":0,"r":0,"a":0.0}},"data":[{"id":"dSource","rows":[[0,48.14814814814815],[1,51.85185185185185]],"fields":[{"id":"fVariable","categories":["Familiar with Google Plus","Not familiar with Google Plus"],"label":"Familiar with GooglePlus","format":{"numericPattern":"###"}},{"min":48.14814814814815,"id":"fVariable1","max":51.85185185185185,"label":"Y Axis"}]}],"titles":[{"backgroundStyle":{"fill":{"g":0,"b":0,"r":0,"a":0.0},"outline":{"g":0,"b":0,"r":0,"a":0.0}},"content":["Familiar with GooglePlus"],"style":{"font":{"weight":"bold","family":"'Helvetica Neue;Helvetica;Arial;SansSerif', sans-serif","size":"12pt"},"fill":{"g":100,"b":157,"r":0},"padding":3.0},"type":"title"}],"copyright":"(C) Copyright IBM Corp. 2011","version":"6.0","size":{"height":500.0,"width":625.0},"grammar":[{"elements":[{"position":[{"field":{"$ref":"fVariable1"}},{"field":{"$ref":"fVariable"}}],"style":{"fill":{"g":178,"b":239,"r":0},"outline":{"g":100,"b":157,"r":0},"size":"75.0%"},"data":{"$ref":"dSource"},"type":"interval"}],"coordinates":{"style":{"fill":{"g":255,"b":255,"r":255},"outline":{"g":100,"b":157,"r":0}},"dimensions":[{"axis":[{"title":["Percent"],"lineStyle":{"stroke":{"width":0.6666667},"fill":{"g":100,"b":157,"r":0}},"tickStyle":{"font":{"weight":"normal","family":"sans-serif","size":"8pt"},"fill":{"g":100,"b":157,"r":0}},"titleStyle":{"font":{"weight":"bold","family":"'Helvetica Neue;Helvetica;Arial;SansSerif', sans-serif","size":"11pt"},"fill":{"g":100,"b":157,"r":0},"padding":6.0},"markStyle":{"stroke":{"width":1.3333334},"fill":{"g":100,"b":157,"r":0}}}],"scale":{"spans":[{"tickDelta":10.0,"outRange":{"min":0.0,"max":1.0}}],"padding":{"right":"5%"}}},{"axis":[{"title":["Familiar with GooglePlus"],"lineStyle":{"stroke":{"width":0.6666667},"fill":{"g":100,"b":157,"r":0}},"tickStyle":{"font":{"weight":"normal","family":"sans-serif","size":"8pt"},"fill":{"g":100,"b":157,"r":0}},"titleStyle":{"font":{"weight":"bold","family":"'Helvetica Neue;Helvetica;Arial;SansSerif', sans-serif","size":"11pt"},"fill":{"g":100,"b":157,"r":0},"padding":6.0},"markStyle":{"stroke":{"width":1.3333334},"fill":{"g":100,"b":157,"r":0}}}],"scale":{"spans":[{"tickDelta":1.0}],"padding":{"left":"5%","right":"5%"}}}]}}]}

Bar Chart  
Bar Chart - Familiar with WikiForums - June 16, 2016

{"style":{"fill":{"g":255,"b":255,"r":255},"outline":{"g":0,"b":0,"r":0,"a":0.0}},"data":[{"id":"dSource","rows":[[0,18.51851851851852],[1,81.48148148148148]],"fields":[{"id":"fVariable","categories":["Familiar with wiki","Not familiar with wiki"],"label":"Familiar with WikiForums","format":{"numericPattern":"###"}},{"min":18.51851851851852,"id":"fVariable1","max":81.48148148148148,"label":"Y Axis"}]}],"titles":[{"backgroundStyle":{"fill":{"g":0,"b":0,"r":0,"a":0.0},"outline":{"g":0,"b":0,"r":0,"a":0.0}},"content":["Familiar with WikiForums"],"style":{"font":{"weight":"bold","family":"'Helvetica Neue;Helvetica;Arial;SansSerif', sans-serif","size":"12pt"},"fill":{"g":100,"b":157,"r":0},"padding":3.0},"type":"title"}],"copyright":"(C) Copyright IBM Corp. 2011","version":"6.0","size":{"height":500.0,"width":625.0},"grammar":[{"elements":[{"position":[{"field":{"$ref":"fVariable1"}},{"field":{"$ref":"fVariable"}}],"style":{"fill":{"g":178,"b":239,"r":0},"outline":{"g":100,"b":157,"r":0},"size":"75.0%"},"data":{"$ref":"dSource"},"type":"interval"}],"coordinates":{"style":{"fill":{"g":255,"b":255,"r":255},"outline":{"g":100,"b":157,"r":0}},"dimensions":[{"axis":[{"title":["Percent"],"lineStyle":{"stroke":{"width":0.6666667},"fill":{"g":100,"b":157,"r":0}},"tickStyle":{"font":{"weight":"normal","family":"sans-serif","size":"8pt"},"fill":{"g":100,"b":157,"r":0}},"titleStyle":{"font":{"weight":"bold","family":"'Helvetica Neue;Helvetica;Arial;SansSerif', sans-serif","size":"11pt"},"fill":{"g":100,"b":157,"r":0},"padding":6.0},"markStyle":{"stroke":{"width":1.3333334},"fill":{"g":100,"b":157,"r":0}}}],"scale":{"spans":[{"tickDelta":20.0,"outRange":{"min":0.0,"max":1.0}}],"padding":{"right":"5%"}}},{"axis":[{"title":["Familiar with WikiForums"],"lineStyle":{"stroke":{"width":0.6666667},"fill":{"g":100,"b":157,"r":0}},"tickStyle":{"font":{"weight":"normal","family":"sans-serif","size":"8pt"},"fill":{"g":100,"b":157,"r":0}},"titleStyle":{"font":{"weight":"bold","family":"'Helvetica Neue;Helvetica;Arial;SansSerif', sans-serif","size":"11pt"},"fill":{"g":100,"b":157,"r":0},"padding":6.0},"markStyle":{"stroke":{"width":1.3333334},"fill":{"g":100,"b":157,"r":0}}}],"scale":{"spans":[{"tickDelta":1.0}],"padding":{"left":"5%","right":"5%"}}}]}}]}

Bar Chart  
Bar Chart - Familiar with Yelp - June 16, 2016

{"style":{"fill":{"g":255,"b":255,"r":255},"outline":{"g":0,"b":0,"r":0,"a":0.0}},"data":[{"id":"dSource","rows":[[0,22.22222222222222],[1,77.77777777777779]],"fields":[{"id":"fVariable","categories":["Familiar with Yelp","Not familiar with Yelp"],"label":"Familiar with Yelp","format":{"numericPattern":"###"}},{"min":22.22222222222222,"id":"fVariable1","max":77.77777777777779,"label":"Y Axis"}]}],"titles":[{"backgroundStyle":{"fill":{"g":0,"b":0,"r":0,"a":0.0},"outline":{"g":0,"b":0,"r":0,"a":0.0}},"content":["Familiar with Yelp"],"style":{"font":{"weight":"bold","family":"'Helvetica Neue;Helvetica;Arial;SansSerif', sans-serif","size":"12pt"},"fill":{"g":100,"b":157,"r":0},"padding":3.0},"type":"title"}],"copyright":"(C) Copyright IBM Corp. 2011","version":"6.0","size":{"height":500.0,"width":625.0},"grammar":[{"elements":[{"position":[{"field":{"$ref":"fVariable1"}},{"field":{"$ref":"fVariable"}}],"style":{"fill":{"g":178,"b":239,"r":0},"outline":{"g":100,"b":157,"r":0},"size":"75.0%"},"data":{"$ref":"dSource"},"type":"interval"}],"coordinates":{"style":{"fill":{"g":255,"b":255,"r":255},"outline":{"g":100,"b":157,"r":0}},"dimensions":[{"axis":[{"title":["Percent"],"lineStyle":{"stroke":{"width":0.6666667},"fill":{"g":100,"b":157,"r":0}},"tickStyle":{"font":{"weight":"normal","family":"sans-serif","size":"8pt"},"fill":{"g":100,"b":157,"r":0}},"titleStyle":{"font":{"weight":"bold","family":"'Helvetica Neue;Helvetica;Arial;SansSerif', sans-serif","size":"11pt"},"fill":{"g":100,"b":157,"r":0},"padding":6.0},"markStyle":{"stroke":{"width":1.3333334},"fill":{"g":100,"b":157,"r":0}}}],"scale":{"spans":[{"tickDelta":20.0,"outRange":{"min":0.0,"max":1.0}}],"padding":{"right":"5%"}}},{"axis":[{"title":["Familiar with Yelp"],"lineStyle":{"stroke":{"width":0.6666667},"fill":{"g":100,"b":157,"r":0}},"tickStyle":{"font":{"weight":"normal","family":"sans-serif","size":"8pt"},"fill":{"g":100,"b":157,"r":0}},"titleStyle":{"font":{"weight":"bold","family":"'Helvetica Neue;Helvetica;Arial;SansSerif', sans-serif","size":"11pt"},"fill":{"g":100,"b":157,"r":0},"padding":6.0},"markStyle":{"stroke":{"width":1.3333334},"fill":{"g":100,"b":157,"r":0}}}],"scale":{"spans":[{"tickDelta":1.0}],"padding":{"left":"5%","right":"5%"}}}]}}]}

Bar Chart  
Bar Chart - Familiar with YouTube - June 16, 2016

{"style":{"fill":{"g":255,"b":255,"r":255},"outline":{"g":0,"b":0,"r":0,"a":0.0}},"data":[{"id":"dSource","rows":[[0,96.29629629629629],[1,3.703703703703703]],"fields":[{"id":"fVariable","categories":["Familiar with YouTube","Not familiar with YouTube"],"label":"Familiar with YouTube","format":{"numericPattern":"###"}},{"min":3.703703703703703,"id":"fVariable1","max":96.29629629629629,"label":"Y Axis"}]}],"titles":[{"backgroundStyle":{"fill":{"g":0,"b":0,"r":0,"a":0.0},"outline":{"g":0,"b":0,"r":0,"a":0.0}},"content":["Familiar with YouTube"],"style":{"font":{"weight":"bold","family":"'Helvetica Neue;Helvetica;Arial;SansSerif', sans-serif","size":"12pt"},"fill":{"g":100,"b":157,"r":0},"padding":3.0},"type":"title"}],"copyright":"(C) Copyright IBM Corp. 2011","version":"6.0","size":{"height":500.0,"width":625.0},"grammar":[{"elements":[{"position":[{"field":{"$ref":"fVariable1"}},{"field":{"$ref":"fVariable"}}],"style":{"fill":{"g":178,"b":239,"r":0},"outline":{"g":100,"b":157,"r":0},"size":"75.0%"},"data":{"$ref":"dSource"},"type":"interval"}],"coordinates":{"style":{"fill":{"g":255,"b":255,"r":255},"outline":{"g":100,"b":157,"r":0}},"dimensions":[{"axis":[{"title":["Percent"],"lineStyle":{"stroke":{"width":0.6666667},"fill":{"g":100,"b":157,"r":0}},"tickStyle":{"font":{"weight":"normal","family":"sans-serif","size":"8pt"},"fill":{"g":100,"b":157,"r":0}},"titleStyle":{"font":{"weight":"bold","family":"'Helvetica Neue;Helvetica;Arial;SansSerif', sans-serif","size":"11pt"},"fill":{"g":100,"b":157,"r":0},"padding":6.0},"markStyle":{"stroke":{"width":1.3333334},"fill":{"g":100,"b":157,"r":0}}}],"scale":{"spans":[{"tickDelta":20.0,"outRange":{"min":0.0,"max":1.0}}],"padding":{"right":"5%"}}},{"axis":[{"title":["Familiar with YouTube"],"lineStyle":{"stroke":{"width":0.6666667},"fill":{"g":100,"b":157,"r":0}},"tickStyle":{"font":{"weight":"normal","family":"sans-serif","size":"8pt"},"fill":{"g":100,"b":157,"r":0}},"titleStyle":{"font":{"weight":"bold","family":"'Helvetica Neue;Helvetica;Arial;SansSerif', sans-serif","size":"11pt"},"fill":{"g":100,"b":157,"r":0},"padding":6.0},"markStyle":{"stroke":{"width":1.3333334},"fill":{"g":100,"b":157,"r":0}}}],"scale":{"spans":[{"tickDelta":1.0}],"padding":{"left":"5%","right":"5%"}}}]}}]}

Bar Chart  
Bar Chart - Familiar with MySpace - June 16, 2016

{"style":{"fill":{"g":255,"b":255,"r":255},"outline":{"g":0,"b":0,"r":0,"a":0.0}},"data":[{"id":"dSource","rows":[[0,40.74074074074074],[1,59.25925925925925]],"fields":[{"id":"fVariable","categories":["Familiar with MySpace","Not familiar with MySpace"],"label":"Familiar with MySpace","format":{"numericPattern":"###"}},{"min":40.74074074074074,"id":"fVariable1","max":59.25925925925925,"label":"Y Axis"}]}],"titles":[{"backgroundStyle":{"fill":{"g":0,"b":0,"r":0,"a":0.0},"outline":{"g":0,"b":0,"r":0,"a":0.0}},"content":["Familiar with MySpace"],"style":{"font":{"weight":"bold","family":"'Helvetica Neue;Helvetica;Arial;SansSerif', sans-serif","size":"12pt"},"fill":{"g":100,"b":157,"r":0},"padding":3.0},"type":"title"}],"copyright":"(C) Copyright IBM Corp. 2011","version":"6.0","size":{"height":500.0,"width":625.0},"grammar":[{"elements":[{"position":[{"field":{"$ref":"fVariable1"}},{"field":{"$ref":"fVariable"}}],"style":{"fill":{"g":178,"b":239,"r":0},"outline":{"g":100,"b":157,"r":0},"size":"75.0%"},"data":{"$ref":"dSource"},"type":"interval"}],"coordinates":{"style":{"fill":{"g":255,"b":255,"r":255},"outline":{"g":100,"b":157,"r":0}},"dimensions":[{"axis":[{"title":["Percent"],"lineStyle":{"stroke":{"width":0.6666667},"fill":{"g":100,"b":157,"r":0}},"tickStyle":{"font":{"weight":"normal","family":"sans-serif","size":"8pt"},"fill":{"g":100,"b":157,"r":0}},"titleStyle":{"font":{"weight":"bold","family":"'Helvetica Neue;Helvetica;Arial;SansSerif', sans-serif","size":"11pt"},"fill":{"g":100,"b":157,"r":0},"padding":6.0},"markStyle":{"stroke":{"width":1.3333334},"fill":{"g":100,"b":157,"r":0}}}],"scale":{"spans":[{"tickDelta":10.0,"outRange":{"min":0.0,"max":1.0}}],"padding":{"right":"5%"}}},{"axis":[{"title":["Familiar with MySpace"],"lineStyle":{"stroke":{"width":0.6666667},"fill":{"g":100,"b":157,"r":0}},"tickStyle":{"font":{"weight":"normal","family":"sans-serif","size":"8pt"},"fill":{"g":100,"b":157,"r":0}},"titleStyle":{"font":{"weight":"bold","family":"'Helvetica Neue;Helvetica;Arial;SansSerif', sans-serif","size":"11pt"},"fill":{"g":100,"b":157,"r":0},"padding":6.0},"markStyle":{"stroke":{"width":1.3333334},"fill":{"g":100,"b":157,"r":0}}}],"scale":{"spans":[{"tickDelta":1.0}],"padding":{"left":"5%","right":"5%"}}}]}}]}

Bar Chart  
Bar Chart - Familiar with Blogs - June 16, 2016

{"style":{"fill":{"g":255,"b":255,"r":255},"outline":{"g":0,"b":0,"r":0,"a":0.0}},"data":[{"id":"dSource","rows":[[0,51.85185185185185],[1,48.14814814814815]],"fields":[{"id":"fVariable","categories":["Familiar with blogs","Not familiar with blogs"],"label":"Familiar with Blogs","format":{"numericPattern":"###"}},{"min":48.14814814814815,"id":"fVariable1","max":51.85185185185185,"label":"Y Axis"}]}],"titles":[{"backgroundStyle":{"fill":{"g":0,"b":0,"r":0,"a":0.0},"outline":{"g":0,"b":0,"r":0,"a":0.0}},"content":["Familiar with Blogs"],"style":{"font":{"weight":"bold","family":"'Helvetica Neue;Helvetica;Arial;SansSerif', sans-serif","size":"12pt"},"fill":{"g":100,"b":157,"r":0},"padding":3.0},"type":"title"}],"copyright":"(C) Copyright IBM Corp. 2011","version":"6.0","size":{"height":500.0,"width":625.0},"grammar":[{"elements":[{"position":[{"field":{"$ref":"fVariable1"}},{"field":{"$ref":"fVariable"}}],"style":{"fill":{"g":178,"b":239,"r":0},"outline":{"g":100,"b":157,"r":0},"size":"75.0%"},"data":{"$ref":"dSource"},"type":"interval"}],"coordinates":{"style":{"fill":{"g":255,"b":255,"r":255},"outline":{"g":100,"b":157,"r":0}},"dimensions":[{"axis":[{"title":["Percent"],"lineStyle":{"stroke":{"width":0.6666667},"fill":{"g":100,"b":157,"r":0}},"tickStyle":{"font":{"weight":"normal","family":"sans-serif","size":"8pt"},"fill":{"g":100,"b":157,"r":0}},"titleStyle":{"font":{"weight":"bold","family":"'Helvetica Neue;Helvetica;Arial;SansSerif', sans-serif","size":"11pt"},"fill":{"g":100,"b":157,"r":0},"padding":6.0},"markStyle":{"stroke":{"width":1.3333334},"fill":{"g":100,"b":157,"r":0}}}],"scale":{"spans":[{"tickDelta":10.0,"outRange":{"min":0.0,"max":1.0}}],"padding":{"right":"5%"}}},{"axis":[{"title":["Familiar with Blogs"],"lineStyle":{"stroke":{"width":0.6666667},"fill":{"g":100,"b":157,"r":0}},"tickStyle":{"font":{"weight":"normal","family":"sans-serif","size":"8pt"},"fill":{"g":100,"b":157,"r":0}},"titleStyle":{"font":{"weight":"bold","family":"'Helvetica Neue;Helvetica;Arial;SansSerif', sans-serif","size":"11pt"},"fill":{"g":100,"b":157,"r":0},"padding":6.0},"markStyle":{"stroke":{"width":1.3333334},"fill":{"g":100,"b":157,"r":0}}}],"scale":{"spans":[{"tickDelta":1.0}],"padding":{"left":"5%","right":"5%"}}}]}}]}

Bar Chart  
Bar Chart - Familiar with Social Bookmarking - June 16, 2016

{"style":{"fill":{"g":255,"b":255,"r":255},"outline":{"g":0,"b":0,"r":0,"a":0.0}},"data":[{"id":"dSource","rows":[[0,33.33333333333333],[1,66.66666666666666]],"fields":[{"id":"fVariable","categories":["Familiar with social bookmarking","Not familiar with social bookmarking"],"label":"Familiar with Social Bookmarking","format":{"numericPattern":"###"}},{"min":33.33333333333333,"id":"fVariable1","max":66.66666666666666,"label":"Y Axis"}]}],"titles":[{"backgroundStyle":{"fill":{"g":0,"b":0,"r":0,"a":0.0},"outline":{"g":0,"b":0,"r":0,"a":0.0}},"content":["Familiar with Social Bookmarking"],"style":{"font":{"weight":"bold","family":"'Helvetica Neue;Helvetica;Arial;SansSerif', sans-serif","size":"12pt"},"fill":{"g":100,"b":157,"r":0},"padding":3.0},"type":"title"}],"copyright":"(C) Copyright IBM Corp. 2011","version":"6.0","size":{"height":500.0,"width":625.0},"grammar":[{"elements":[{"position":[{"field":{"$ref":"fVariable1"}},{"field":{"$ref":"fVariable"}}],"style":{"fill":{"g":178,"b":239,"r":0},"outline":{"g":100,"b":157,"r":0},"size":"75.0%"},"data":{"$ref":"dSource"},"type":"interval"}],"coordinates":{"style":{"fill":{"g":255,"b":255,"r":255},"outline":{"g":100,"b":157,"r":0}},"dimensions":[{"axis":[{"title":["Percent"],"lineStyle":{"stroke":{"width":0.6666667},"fill":{"g":100,"b":157,"r":0}},"tickStyle":{"font":{"weight":"normal","family":"sans-serif","size":"8pt"},"fill":{"g":100,"b":157,"r":0}},"titleStyle":{"font":{"weight":"bold","family":"'Helvetica Neue;Helvetica;Arial;SansSerif', sans-serif","size":"11pt"},"fill":{"g":100,"b":157,"r":0},"padding":6.0},"markStyle":{"stroke":{"width":1.3333334},"fill":{"g":100,"b":157,"r":0}}}],"scale":{"spans":[{"tickDelta":20.0,"outRange":{"min":0.0,"max":1.0}}],"padding":{"right":"5%"}}},{"axis":[{"title":["Familiar with Social Bookmarking"],"lineStyle":{"stroke":{"width":0.6666667},"fill":{"g":100,"b":157,"r":0}},"tickStyle":{"font":{"weight":"normal","family":"sans-serif","size":"8pt"},"fill":{"g":100,"b":157,"r":0}},"titleStyle":{"font":{"weight":"bold","family":"'Helvetica Neue;Helvetica;Arial;SansSerif', sans-serif","size":"11pt"},"fill":{"g":100,"b":157,"r":0},"padding":6.0},"markStyle":{"stroke":{"width":1.3333334},"fill":{"g":100,"b":157,"r":0}}}],"scale":{"spans":[{"tickDelta":1.0}],"padding":{"left":"5%","right":"5%"}}}]}}]}

Bar Chart  
Bar Chart - Aggregate of number of social media sites familiar - June 16, 2016

{"style":{"fill":{"g":255,"b":255,"r":255},"outline":{"g":0,"b":0,"r":0,"a":0.0}},"data":[{"id":"dSource","rows":[[0,3.703703703703703],[1,11.11111111111111],[2,18.51851851851852],[3,14.81481481481481],[4,14.81481481481481],[5,3.703703703703703],[6,3.703703703703703],[7,11.11111111111111],[8,3.703703703703703],[9,11.11111111111111],[10,3.703703703703703]],"fields":[{"id":"fVariable","categories":["1.00","3.00","4.00","5.00","6.00","8.00","9.00","10.00","11.00","12.00","13.00"],"label":"Aggregate of number of social media sites familiar","format":{"numericPattern":"###"}},{"min":3.703703703703703,"id":"fVariable1","max":18.51851851851852,"label":"Y Axis"}]}],"titles":[{"backgroundStyle":{"fill":{"g":0,"b":0,"r":0,"a":0.0},"outline":{"g":0,"b":0,"r":0,"a":0.0}},"content":["Aggregate of number of social media sites familiar"],"style":{"font":{"weight":"bold","family":"'Helvetica Neue;Helvetica;Arial;SansSerif', sans-serif","size":"12pt"},"fill":{"g":100,"b":157,"r":0},"padding":3.0},"type":"title"}],"copyright":"(C) Copyright IBM Corp. 2011","version":"6.0","size":{"height":500.0,"width":625.0},"grammar":[{"elements":[{"position":[{"field":{"$ref":"fVariable1"}},{"field":{"$ref":"fVariable"}}],"style":{"fill":{"g":178,"b":239,"r":0},"outline":{"g":100,"b":157,"r":0},"size":"75.0%"},"data":{"$ref":"dSource"},"type":"interval"}],"coordinates":{"style":{"fill":{"g":255,"b":255,"r":255},"outline":{"g":100,"b":157,"r":0}},"dimensions":[{"axis":[{"title":["Percent"],"lineStyle":{"stroke":{"width":0.6666667},"fill":{"g":100,"b":157,"r":0}},"tickStyle":{"font":{"weight":"normal","family":"sans-serif","size":"8pt"},"fill":{"g":100,"b":157,"r":0}},"titleStyle":{"font":{"weight":"bold","family":"'Helvetica Neue;Helvetica;Arial;SansSerif', sans-serif","size":"11pt"},"fill":{"g":100,"b":157,"r":0},"padding":6.0},"markStyle":{"stroke":{"width":1.3333334},"fill":{"g":100,"b":157,"r":0}}}],"scale":{"spans":[{"tickDelta":5.0,"outRange":{"min":0.0,"max":1.0}}],"padding":{"right":"5%"}}},{"axis":[{"title":["Aggregate of number of social media sites familiar"],"lineStyle":{"stroke":{"width":0.6666667},"fill":{"g":100,"b":157,"r":0}},"tickStyle":{"font":{"weight":"normal","family":"sans-serif","size":"8pt"},"fill":{"g":100,"b":157,"r":0}},"titleStyle":{"font":{"weight":"bold","family":"'Helvetica Neue;Helvetica;Arial;SansSerif', sans-serif","size":"11pt"},"fill":{"g":100,"b":157,"r":0},"padding":6.0},"markStyle":{"stroke":{"width":1.3333334},"fill":{"g":100,"b":157,"r":0}}}],"scale":{"spans":[{"tickDelta":1.0}],"padding":{"left":"5%","right":"5%"}}}]}}]}

Bar Chart  
Bar Chart - Joined Facebook - June 16, 2016

{"style":{"fill":{"g":255,"b":255,"r":255},"outline":{"g":0,"b":0,"r":0,"a":0.0}},"data":[{"id":"dSource","rows":[[0,88.88888888888889],[1,11.11111111111111]],"fields":[{"id":"fVariable","categories":["Joined Facebook","Not joined Facebook"],"label":"Joined Facebook","format":{"numericPattern":"###"}},{"min":11.11111111111111,"id":"fVariable1","max":88.88888888888889,"label":"Y Axis"}]}],"titles":[{"backgroundStyle":{"fill":{"g":0,"b":0,"r":0,"a":0.0},"outline":{"g":0,"b":0,"r":0,"a":0.0}},"content":["Joined Facebook"],"style":{"font":{"weight":"bold","family":"'Helvetica Neue;Helvetica;Arial;SansSerif', sans-serif","size":"12pt"},"fill":{"g":100,"b":157,"r":0},"padding":3.0},"type":"title"}],"copyright":"(C) Copyright IBM Corp. 2011","version":"6.0","size":{"height":500.0,"width":625.0},"grammar":[{"elements":[{"position":[{"field":{"$ref":"fVariable1"}},{"field":{"$ref":"fVariable"}}],"style":{"fill":{"g":178,"b":239,"r":0},"outline":{"g":100,"b":157,"r":0},"size":"75.0%"},"data":{"$ref":"dSource"},"type":"interval"}],"coordinates":{"style":{"fill":{"g":255,"b":255,"r":255},"outline":{"g":100,"b":157,"r":0}},"dimensions":[{"axis":[{"title":["Percent"],"lineStyle":{"stroke":{"width":0.6666667},"fill":{"g":100,"b":157,"r":0}},"tickStyle":{"font":{"weight":"normal","family":"sans-serif","size":"8pt"},"fill":{"g":100,"b":157,"r":0}},"titleStyle":{"font":{"weight":"bold","family":"'Helvetica Neue;Helvetica;Arial;SansSerif', sans-serif","size":"11pt"},"fill":{"g":100,"b":157,"r":0},"padding":6.0},"markStyle":{"stroke":{"width":1.3333334},"fill":{"g":100,"b":157,"r":0}}}],"scale":{"spans":[{"tickDelta":20.0,"outRange":{"min":0.0,"max":1.0}}],"padding":{"right":"5%"}}},{"axis":[{"title":["Joined Facebook"],"lineStyle":{"stroke":{"width":0.6666667},"fill":{"g":100,"b":157,"r":0}},"tickStyle":{"font":{"weight":"normal","family":"sans-serif","size":"8pt"},"fill":{"g":100,"b":157,"r":0}},"titleStyle":{"font":{"weight":"bold","family":"'Helvetica Neue;Helvetica;Arial;SansSerif', sans-serif","size":"11pt"},"fill":{"g":100,"b":157,"r":0},"padding":6.0},"markStyle":{"stroke":{"width":1.3333334},"fill":{"g":100,"b":157,"r":0}}}],"scale":{"spans":[{"tickDelta":1.0}],"padding":{"left":"5%","right":"5%"}}}]}}]}

Bar Chart  
Bar Chart - Joined Twitter - June 16, 2016

{"style":{"fill":{"g":255,"b":255,"r":255},"outline":{"g":0,"b":0,"r":0,"a":0.0}},"data":[{"id":"dSource","rows":[[0,66.66666666666666],[1,33.33333333333333]],"fields":[{"id":"fVariable","categories":["Joined Twitter","Not joined Twitter"],"label":"Joined Twitter","format":{"numericPattern":"###"}},{"min":33.33333333333333,"id":"fVariable1","max":66.66666666666666,"label":"Y Axis"}]}],"titles":[{"backgroundStyle":{"fill":{"g":0,"b":0,"r":0,"a":0.0},"outline":{"g":0,"b":0,"r":0,"a":0.0}},"content":["Joined Twitter"],"style":{"font":{"weight":"bold","family":"'Helvetica Neue;Helvetica;Arial;SansSerif', sans-serif","size":"12pt"},"fill":{"g":100,"b":157,"r":0},"padding":3.0},"type":"title"}],"copyright":"(C) Copyright IBM Corp. 2011","version":"6.0","size":{"height":500.0,"width":625.0},"grammar":[{"elements":[{"position":[{"field":{"$ref":"fVariable1"}},{"field":{"$ref":"fVariable"}}],"style":{"fill":{"g":178,"b":239,"r":0},"outline":{"g":100,"b":157,"r":0},"size":"75.0%"},"data":{"$ref":"dSource"},"type":"interval"}],"coordinates":{"style":{"fill":{"g":255,"b":255,"r":255},"outline":{"g":100,"b":157,"r":0}},"dimensions":[{"axis":[{"title":["Percent"],"lineStyle":{"stroke":{"width":0.6666667},"fill":{"g":100,"b":157,"r":0}},"tickStyle":{"font":{"weight":"normal","family":"sans-serif","size":"8pt"},"fill":{"g":100,"b":157,"r":0}},"titleStyle":{"font":{"weight":"bold","family":"'Helvetica Neue;Helvetica;Arial;SansSerif', sans-serif","size":"11pt"},"fill":{"g":100,"b":157,"r":0},"padding":6.0},"markStyle":{"stroke":{"width":1.3333334},"fill":{"g":100,"b":157,"r":0}}}],"scale":{"spans":[{"tickDelta":20.0,"outRange":{"min":0.0,"max":1.0}}],"padding":{"right":"5%"}}},{"axis":[{"title":["Joined Twitter"],"lineStyle":{"stroke":{"width":0.6666667},"fill":{"g":100,"b":157,"r":0}},"tickStyle":{"font":{"weight":"normal","family":"sans-serif","size":"8pt"},"fill":{"g":100,"b":157,"r":0}},"titleStyle":{"font":{"weight":"bold","family":"'Helvetica Neue;Helvetica;Arial;SansSerif', sans-serif","size":"11pt"},"fill":{"g":100,"b":157,"r":0},"padding":6.0},"markStyle":{"stroke":{"width":1.3333334},"fill":{"g":100,"b":157,"r":0}}}],"scale":{"spans":[{"tickDelta":1.0}],"padding":{"left":"5%","right":"5%"}}}]}}]}

Bar Chart  
Bar Chart - Joined Pinterest - June 16, 2016

{"style":{"fill":{"g":255,"b":255,"r":255},"outline":{"g":0,"b":0,"r":0,"a":0.0}},"data":[{"id":"dSource","rows":[[0,37.03703703703704],[1,62.96296296296296]],"fields":[{"id":"fVariable","categories":["Joined Pinterest","Not joined Pinterest"],"label":"Joined Pinterest","format":{"numericPattern":"###"}},{"min":37.03703703703704,"id":"fVariable1","max":62.96296296296296,"label":"Y Axis"}]}],"titles":[{"backgroundStyle":{"fill":{"g":0,"b":0,"r":0,"a":0.0},"outline":{"g":0,"b":0,"r":0,"a":0.0}},"content":["Joined Pinterest"],"style":{"font":{"weight":"bold","family":"'Helvetica Neue;Helvetica;Arial;SansSerif', sans-serif","size":"12pt"},"fill":{"g":100,"b":157,"r":0},"padding":3.0},"type":"title"}],"copyright":"(C) Copyright IBM Corp. 2011","version":"6.0","size":{"height":500.0,"width":625.0},"grammar":[{"elements":[{"position":[{"field":{"$ref":"fVariable1"}},{"field":{"$ref":"fVariable"}}],"style":{"fill":{"g":178,"b":239,"r":0},"outline":{"g":100,"b":157,"r":0},"size":"75.0%"},"data":{"$ref":"dSource"},"type":"interval"}],"coordinates":{"style":{"fill":{"g":255,"b":255,"r":255},"outline":{"g":100,"b":157,"r":0}},"dimensions":[{"axis":[{"title":["Percent"],"lineStyle":{"stroke":{"width":0.6666667},"fill":{"g":100,"b":157,"r":0}},"tickStyle":{"font":{"weight":"normal","family":"sans-serif","size":"8pt"},"fill":{"g":100,"b":157,"r":0}},"titleStyle":{"font":{"weight":"bold","family":"'Helvetica Neue;Helvetica;Arial;SansSerif', sans-serif","size":"11pt"},"fill":{"g":100,"b":157,"r":0},"padding":6.0},"markStyle":{"stroke":{"width":1.3333334},"fill":{"g":100,"b":157,"r":0}}}],"scale":{"spans":[{"tickDelta":20.0,"outRange":{"min":0.0,"max":1.0}}],"padding":{"right":"5%"}}},{"axis":[{"title":["Joined Pinterest"],"lineStyle":{"stroke":{"width":0.6666667},"fill":{"g":100,"b":157,"r":0}},"tickStyle":{"font":{"weight":"normal","family":"sans-serif","size":"8pt"},"fill":{"g":100,"b":157,"r":0}},"titleStyle":{"font":{"weight":"bold","family":"'Helvetica Neue;Helvetica;Arial;SansSerif', sans-serif","size":"11pt"},"fill":{"g":100,"b":157,"r":0},"padding":6.0},"markStyle":{"stroke":{"width":1.3333334},"fill":{"g":100,"b":157,"r":0}}}],"scale":{"spans":[{"tickDelta":1.0}],"padding":{"left":"5%","right":"5%"}}}]}}]}

Bar Chart  
Bar Chart - Joined LinkedIn - June 16, 2016

{"style":{"fill":{"g":255,"b":255,"r":255},"outline":{"g":0,"b":0,"r":0,"a":0.0}},"data":[{"id":"dSource","rows":[[0,55.55555555555556],[1,44.44444444444444]],"fields":[{"id":"fVariable","categories":["Joined LinkedIn","Not joined LinkedIn"],"label":"Joined LinkedIn","format":{"numericPattern":"###"}},{"min":44.44444444444444,"id":"fVariable1","max":55.55555555555556,"label":"Y Axis"}]}],"titles":[{"backgroundStyle":{"fill":{"g":0,"b":0,"r":0,"a":0.0},"outline":{"g":0,"b":0,"r":0,"a":0.0}},"content":["Joined LinkedIn"],"style":{"font":{"weight":"bold","family":"'Helvetica Neue;Helvetica;Arial;SansSerif', sans-serif","size":"12pt"},"fill":{"g":100,"b":157,"r":0},"padding":3.0},"type":"title"}],"copyright":"(C) Copyright IBM Corp. 2011","version":"6.0","size":{"height":500.0,"width":625.0},"grammar":[{"elements":[{"position":[{"field":{"$ref":"fVariable1"}},{"field":{"$ref":"fVariable"}}],"style":{"fill":{"g":178,"b":239,"r":0},"outline":{"g":100,"b":157,"r":0},"size":"75.0%"},"data":{"$ref":"dSource"},"type":"interval"}],"coordinates":{"style":{"fill":{"g":255,"b":255,"r":255},"outline":{"g":100,"b":157,"r":0}},"dimensions":[{"axis":[{"title":["Percent"],"lineStyle":{"stroke":{"width":0.6666667},"fill":{"g":100,"b":157,"r":0}},"tickStyle":{"font":{"weight":"normal","family":"sans-serif","size":"8pt"},"fill":{"g":100,"b":157,"r":0}},"titleStyle":{"font":{"weight":"bold","family":"'Helvetica Neue;Helvetica;Arial;SansSerif', sans-serif","size":"11pt"},"fill":{"g":100,"b":157,"r":0},"padding":6.0},"markStyle":{"stroke":{"width":1.3333334},"fill":{"g":100,"b":157,"r":0}}}],"scale":{"spans":[{"tickDelta":10.0,"outRange":{"min":0.0,"max":1.0}}],"padding":{"right":"5%"}}},{"axis":[{"title":["Joined LinkedIn"],"lineStyle":{"stroke":{"width":0.6666667},"fill":{"g":100,"b":157,"r":0}},"tickStyle":{"font":{"weight":"normal","family":"sans-serif","size":"8pt"},"fill":{"g":100,"b":157,"r":0}},"titleStyle":{"font":{"weight":"bold","family":"'Helvetica Neue;Helvetica;Arial;SansSerif', sans-serif","size":"11pt"},"fill":{"g":100,"b":157,"r":0},"padding":6.0},"markStyle":{"stroke":{"width":1.3333334},"fill":{"g":100,"b":157,"r":0}}}],"scale":{"spans":[{"tickDelta":1.0}],"padding":{"left":"5%","right":"5%"}}}]}}]}

Bar Chart  
Bar Chart - Joined Foursquare - June 16, 2016

{"style":{"fill":{"g":255,"b":255,"r":255},"outline":{"g":0,"b":0,"r":0,"a":0.0}},"data":[{"id":"dSource","rows":[[0,22.22222222222222],[1,77.77777777777779]],"fields":[{"id":"fVariable","categories":["Joined Foursquare","Not joined Foursquare"],"label":"Joined Foursquare","format":{"numericPattern":"###"}},{"min":22.22222222222222,"id":"fVariable1","max":77.77777777777779,"label":"Y Axis"}]}],"titles":[{"backgroundStyle":{"fill":{"g":0,"b":0,"r":0,"a":0.0},"outline":{"g":0,"b":0,"r":0,"a":0.0}},"content":["Joined Foursquare"],"style":{"font":{"weight":"bold","family":"'Helvetica Neue;Helvetica;Arial;SansSerif', sans-serif","size":"12pt"},"fill":{"g":100,"b":157,"r":0},"padding":3.0},"type":"title"}],"copyright":"(C) Copyright IBM Corp. 2011","version":"6.0","size":{"height":500.0,"width":625.0},"grammar":[{"elements":[{"position":[{"field":{"$ref":"fVariable1"}},{"field":{"$ref":"fVariable"}}],"style":{"fill":{"g":178,"b":239,"r":0},"outline":{"g":100,"b":157,"r":0},"size":"75.0%"},"data":{"$ref":"dSource"},"type":"interval"}],"coordinates":{"style":{"fill":{"g":255,"b":255,"r":255},"outline":{"g":100,"b":157,"r":0}},"dimensions":[{"axis":[{"title":["Percent"],"lineStyle":{"stroke":{"width":0.6666667},"fill":{"g":100,"b":157,"r":0}},"tickStyle":{"font":{"weight":"normal","family":"sans-serif","size":"8pt"},"fill":{"g":100,"b":157,"r":0}},"titleStyle":{"font":{"weight":"bold","family":"'Helvetica Neue;Helvetica;Arial;SansSerif', sans-serif","size":"11pt"},"fill":{"g":100,"b":157,"r":0},"padding":6.0},"markStyle":{"stroke":{"width":1.3333334},"fill":{"g":100,"b":157,"r":0}}}],"scale":{"spans":[{"tickDelta":20.0,"outRange":{"min":0.0,"max":1.0}}],"padding":{"right":"5%"}}},{"axis":[{"title":["Joined Foursquare"],"lineStyle":{"stroke":{"width":0.6666667},"fill":{"g":100,"b":157,"r":0}},"tickStyle":{"font":{"weight":"normal","family":"sans-serif","size":"8pt"},"fill":{"g":100,"b":157,"r":0}},"titleStyle":{"font":{"weight":"bold","family":"'Helvetica Neue;Helvetica;Arial;SansSerif', sans-serif","size":"11pt"},"fill":{"g":100,"b":157,"r":0},"padding":6.0},"markStyle":{"stroke":{"width":1.3333334},"fill":{"g":100,"b":157,"r":0}}}],"scale":{"spans":[{"tickDelta":1.0}],"padding":{"left":"5%","right":"5%"}}}]}}]}

Bar Chart  
Bar Chart - Joined Tumblr - June 16, 2016

{"style":{"fill":{"g":255,"b":255,"r":255},"outline":{"g":0,"b":0,"r":0,"a":0.0}},"data":[{"id":"dSource","rows":[[0,18.51851851851852],[1,81.48148148148148]],"fields":[{"id":"fVariable","categories":["Joined Tumblr","Not joined Tumblr"],"label":"Joined Tumblr","format":{"numericPattern":"###"}},{"min":18.51851851851852,"id":"fVariable1","max":81.48148148148148,"label":"Y Axis"}]}],"titles":[{"backgroundStyle":{"fill":{"g":0,"b":0,"r":0,"a":0.0},"outline":{"g":0,"b":0,"r":0,"a":0.0}},"content":["Joined Tumblr"],"style":{"font":{"weight":"bold","family":"'Helvetica Neue;Helvetica;Arial;SansSerif', sans-serif","size":"12pt"},"fill":{"g":100,"b":157,"r":0},"padding":3.0},"type":"title"}],"copyright":"(C) Copyright IBM Corp. 2011","version":"6.0","size":{"height":500.0,"width":625.0},"grammar":[{"elements":[{"position":[{"field":{"$ref":"fVariable1"}},{"field":{"$ref":"fVariable"}}],"style":{"fill":{"g":178,"b":239,"r":0},"outline":{"g":100,"b":157,"r":0},"size":"75.0%"},"data":{"$ref":"dSource"},"type":"interval"}],"coordinates":{"style":{"fill":{"g":255,"b":255,"r":255},"outline":{"g":100,"b":157,"r":0}},"dimensions":[{"axis":[{"title":["Percent"],"lineStyle":{"stroke":{"width":0.6666667},"fill":{"g":100,"b":157,"r":0}},"tickStyle":{"font":{"weight":"normal","family":"sans-serif","size":"8pt"},"fill":{"g":100,"b":157,"r":0}},"titleStyle":{"font":{"weight":"bold","family":"'Helvetica Neue;Helvetica;Arial;SansSerif', sans-serif","size":"11pt"},"fill":{"g":100,"b":157,"r":0},"padding":6.0},"markStyle":{"stroke":{"width":1.3333334},"fill":{"g":100,"b":157,"r":0}}}],"scale":{"spans":[{"tickDelta":20.0,"outRange":{"min":0.0,"max":1.0}}],"padding":{"right":"5%"}}},{"axis":[{"title":["Joined Tumblr"],"lineStyle":{"stroke":{"width":0.6666667},"fill":{"g":100,"b":157,"r":0}},"tickStyle":{"font":{"weight":"normal","family":"sans-serif","size":"8pt"},"fill":{"g":100,"b":157,"r":0}},"titleStyle":{"font":{"weight":"bold","family":"'Helvetica Neue;Helvetica;Arial;SansSerif', sans-serif","size":"11pt"},"fill":{"g":100,"b":157,"r":0},"padding":6.0},"markStyle":{"stroke":{"width":1.3333334},"fill":{"g":100,"b":157,"r":0}}}],"scale":{"spans":[{"tickDelta":1.0}],"padding":{"left":"5%","right":"5%"}}}]}}]}

Bar Chart  
Bar Chart - Joined Google Plus - June 16, 2016

{"style":{"fill":{"g":255,"b":255,"r":255},"outline":{"g":0,"b":0,"r":0,"a":0.0}},"data":[{"id":"dSource","rows":[[0,22.22222222222222],[1,77.77777777777779]],"fields":[{"id":"fVariable","categories":["Joined Google Plus","Not joined Google Plus"],"label":"Joined Google Plus","format":{"numericPattern":"###"}},{"min":22.22222222222222,"id":"fVariable1","max":77.77777777777779,"label":"Y Axis"}]}],"titles":[{"backgroundStyle":{"fill":{"g":0,"b":0,"r":0,"a":0.0},"outline":{"g":0,"b":0,"r":0,"a":0.0}},"content":["Joined Google Plus"],"style":{"font":{"weight":"bold","family":"'Helvetica Neue;Helvetica;Arial;SansSerif', sans-serif","size":"12pt"},"fill":{"g":100,"b":157,"r":0},"padding":3.0},"type":"title"}],"copyright":"(C) Copyright IBM Corp. 2011","version":"6.0","size":{"height":500.0,"width":625.0},"grammar":[{"elements":[{"position":[{"field":{"$ref":"fVariable1"}},{"field":{"$ref":"fVariable"}}],"style":{"fill":{"g":178,"b":239,"r":0},"outline":{"g":100,"b":157,"r":0},"size":"75.0%"},"data":{"$ref":"dSource"},"type":"interval"}],"coordinates":{"style":{"fill":{"g":255,"b":255,"r":255},"outline":{"g":100,"b":157,"r":0}},"dimensions":[{"axis":[{"title":["Percent"],"lineStyle":{"stroke":{"width":0.6666667},"fill":{"g":100,"b":157,"r":0}},"tickStyle":{"font":{"weight":"normal","family":"sans-serif","size":"8pt"},"fill":{"g":100,"b":157,"r":0}},"titleStyle":{"font":{"weight":"bold","family":"'Helvetica Neue;Helvetica;Arial;SansSerif', sans-serif","size":"11pt"},"fill":{"g":100,"b":157,"r":0},"padding":6.0},"markStyle":{"stroke":{"width":1.3333334},"fill":{"g":100,"b":157,"r":0}}}],"scale":{"spans":[{"tickDelta":20.0,"outRange":{"min":0.0,"max":1.0}}],"padding":{"right":"5%"}}},{"axis":[{"title":["Joined Google Plus"],"lineStyle":{"stroke":{"width":0.6666667},"fill":{"g":100,"b":157,"r":0}},"tickStyle":{"font":{"weight":"normal","family":"sans-serif","size":"8pt"},"fill":{"g":100,"b":157,"r":0}},"titleStyle":{"font":{"weight":"bold","family":"'Helvetica Neue;Helvetica;Arial;SansSerif', sans-serif","size":"11pt"},"fill":{"g":100,"b":157,"r":0},"padding":6.0},"markStyle":{"stroke":{"width":1.3333334},"fill":{"g":100,"b":157,"r":0}}}],"scale":{"spans":[{"tickDelta":1.0}],"padding":{"left":"5%","right":"5%"}}}]}}]}

Bar Chart  
Bar Chart - Joined YouTube - June 16, 2016

{"style":{"fill":{"g":255,"b":255,"r":255},"outline":{"g":0,"b":0,"r":0,"a":0.0}},"data":[{"id":"dSource","rows":[[0,85.18518518518519],[1,14.81481481481481]],"fields":[{"id":"fVariable","categories":["Joined YouTube","Not joined YouTube"],"label":"Joined YouTube","format":{"numericPattern":"###"}},{"min":14.81481481481481,"id":"fVariable1","max":85.18518518518519,"label":"Y Axis"}]}],"titles":[{"backgroundStyle":{"fill":{"g":0,"b":0,"r":0,"a":0.0},"outline":{"g":0,"b":0,"r":0,"a":0.0}},"content":["Joined YouTube"],"style":{"font":{"weight":"bold","family":"'Helvetica Neue;Helvetica;Arial;SansSerif', sans-serif","size":"12pt"},"fill":{"g":100,"b":157,"r":0},"padding":3.0},"type":"title"}],"copyright":"(C) Copyright IBM Corp. 2011","version":"6.0","size":{"height":500.0,"width":625.0},"grammar":[{"elements":[{"position":[{"field":{"$ref":"fVariable1"}},{"field":{"$ref":"fVariable"}}],"style":{"fill":{"g":178,"b":239,"r":0},"outline":{"g":100,"b":157,"r":0},"size":"75.0%"},"data":{"$ref":"dSource"},"type":"interval"}],"coordinates":{"style":{"fill":{"g":255,"b":255,"r":255},"outline":{"g":100,"b":157,"r":0}},"dimensions":[{"axis":[{"title":["Percent"],"lineStyle":{"stroke":{"width":0.6666667},"fill":{"g":100,"b":157,"r":0}},"tickStyle":{"font":{"weight":"normal","family":"sans-serif","size":"8pt"},"fill":{"g":100,"b":157,"r":0}},"titleStyle":{"font":{"weight":"bold","family":"'Helvetica Neue;Helvetica;Arial;SansSerif', sans-serif","size":"11pt"},"fill":{"g":100,"b":157,"r":0},"padding":6.0},"markStyle":{"stroke":{"width":1.3333334},"fill":{"g":100,"b":157,"r":0}}}],"scale":{"spans":[{"tickDelta":20.0,"outRange":{"min":0.0,"max":1.0}}],"padding":{"right":"5%"}}},{"axis":[{"title":["Joined YouTube"],"lineStyle":{"stroke":{"width":0.6666667},"fill":{"g":100,"b":157,"r":0}},"tickStyle":{"font":{"weight":"normal","family":"sans-serif","size":"8pt"},"fill":{"g":100,"b":157,"r":0}},"titleStyle":{"font":{"weight":"bold","family":"'Helvetica Neue;Helvetica;Arial;SansSerif', sans-serif","size":"11pt"},"fill":{"g":100,"b":157,"r":0},"padding":6.0},"markStyle":{"stroke":{"width":1.3333334},"fill":{"g":100,"b":157,"r":0}}}],"scale":{"spans":[{"tickDelta":1.0}],"padding":{"left":"5%","right":"5%"}}}]}}]}

Bar Chart  
Bar Chart - Joined MySpace - June 16, 2016

{"style":{"fill":{"g":255,"b":255,"r":255},"outline":{"g":0,"b":0,"r":0,"a":0.0}},"data":[{"id":"dSource","rows":[[0,22.22222222222222],[1,77.77777777777779]],"fields":[{"id":"fVariable","categories":["Joined Myspace","Not joined Myspace"],"label":"Joined MySpace","format":{"numericPattern":"###"}},{"min":22.22222222222222,"id":"fVariable1","max":77.77777777777779,"label":"Y Axis"}]}],"titles":[{"backgroundStyle":{"fill":{"g":0,"b":0,"r":0,"a":0.0},"outline":{"g":0,"b":0,"r":0,"a":0.0}},"content":["Joined MySpace"],"style":{"font":{"weight":"bold","family":"'Helvetica Neue;Helvetica;Arial;SansSerif', sans-serif","size":"12pt"},"fill":{"g":100,"b":157,"r":0},"padding":3.0},"type":"title"}],"copyright":"(C) Copyright IBM Corp. 2011","version":"6.0","size":{"height":500.0,"width":625.0},"grammar":[{"elements":[{"position":[{"field":{"$ref":"fVariable1"}},{"field":{"$ref":"fVariable"}}],"style":{"fill":{"g":178,"b":239,"r":0},"outline":{"g":100,"b":157,"r":0},"size":"75.0%"},"data":{"$ref":"dSource"},"type":"interval"}],"coordinates":{"style":{"fill":{"g":255,"b":255,"r":255},"outline":{"g":100,"b":157,"r":0}},"dimensions":[{"axis":[{"title":["Percent"],"lineStyle":{"stroke":{"width":0.6666667},"fill":{"g":100,"b":157,"r":0}},"tickStyle":{"font":{"weight":"normal","family":"sans-serif","size":"8pt"},"fill":{"g":100,"b":157,"r":0}},"titleStyle":{"font":{"weight":"bold","family":"'Helvetica Neue;Helvetica;Arial;SansSerif', sans-serif","size":"11pt"},"fill":{"g":100,"b":157,"r":0},"padding":6.0},"markStyle":{"stroke":{"width":1.3333334},"fill":{"g":100,"b":157,"r":0}}}],"scale":{"spans":[{"tickDelta":20.0,"outRange":{"min":0.0,"max":1.0}}],"padding":{"right":"5%"}}},{"axis":[{"title":["Joined MySpace"],"lineStyle":{"stroke":{"width":0.6666667},"fill":{"g":100,"b":157,"r":0}},"tickStyle":{"font":{"weight":"normal","family":"sans-serif","size":"8pt"},"fill":{"g":100,"b":157,"r":0}},"titleStyle":{"font":{"weight":"bold","family":"'Helvetica Neue;Helvetica;Arial;SansSerif', sans-serif","size":"11pt"},"fill":{"g":100,"b":157,"r":0},"padding":6.0},"markStyle":{"stroke":{"width":1.3333334},"fill":{"g":100,"b":157,"r":0}}}],"scale":{"spans":[{"tickDelta":1.0}],"padding":{"left":"5%","right":"5%"}}}]}}]}

Bar Chart  
Bar Chart - Joined blogs - June 16, 2016

{"style":{"fill":{"g":255,"b":255,"r":255},"outline":{"g":0,"b":0,"r":0,"a":0.0}},"data":[{"id":"dSource","rows":[[0,37.03703703703704],[1,62.96296296296296]],"fields":[{"id":"fVariable","categories":["Joined blogs","Not joined blogs"],"label":"Joined blogs","format":{"numericPattern":"###"}},{"min":37.03703703703704,"id":"fVariable1","max":62.96296296296296,"label":"Y Axis"}]}],"titles":[{"backgroundStyle":{"fill":{"g":0,"b":0,"r":0,"a":0.0},"outline":{"g":0,"b":0,"r":0,"a":0.0}},"content":["Joined blogs"],"style":{"font":{"weight":"bold","family":"'Helvetica Neue;Helvetica;Arial;SansSerif', sans-serif","size":"12pt"},"fill":{"g":100,"b":157,"r":0},"padding":3.0},"type":"title"}],"copyright":"(C) Copyright IBM Corp. 2011","version":"6.0","size":{"height":500.0,"width":625.0},"grammar":[{"elements":[{"position":[{"field":{"$ref":"fVariable1"}},{"field":{"$ref":"fVariable"}}],"style":{"fill":{"g":178,"b":239,"r":0},"outline":{"g":100,"b":157,"r":0},"size":"75.0%"},"data":{"$ref":"dSource"},"type":"interval"}],"coordinates":{"style":{"fill":{"g":255,"b":255,"r":255},"outline":{"g":100,"b":157,"r":0}},"dimensions":[{"axis":[{"title":["Percent"],"lineStyle":{"stroke":{"width":0.6666667},"fill":{"g":100,"b":157,"r":0}},"tickStyle":{"font":{"weight":"normal","family":"sans-serif","size":"8pt"},"fill":{"g":100,"b":157,"r":0}},"titleStyle":{"font":{"weight":"bold","family":"'Helvetica Neue;Helvetica;Arial;SansSerif', sans-serif","size":"11pt"},"fill":{"g":100,"b":157,"r":0},"padding":6.0},"markStyle":{"stroke":{"width":1.3333334},"fill":{"g":100,"b":157,"r":0}}}],"scale":{"spans":[{"tickDelta":20.0,"outRange":{"min":0.0,"max":1.0}}],"padding":{"right":"5%"}}},{"axis":[{"title":["Joined blogs"],"lineStyle":{"stroke":{"width":0.6666667},"fill":{"g":100,"b":157,"r":0}},"tickStyle":{"font":{"weight":"normal","family":"sans-serif","size":"8pt"},"fill":{"g":100,"b":157,"r":0}},"titleStyle":{"font":{"weight":"bold","family":"'Helvetica Neue;Helvetica;Arial;SansSerif', sans-serif","size":"11pt"},"fill":{"g":100,"b":157,"r":0},"padding":6.0},"markStyle":{"stroke":{"width":1.3333334},"fill":{"g":100,"b":157,"r":0}}}],"scale":{"spans":[{"tickDelta":1.0}],"padding":{"left":"5%","right":"5%"}}}]}}]}

Bar Chart  
Bar Chart - Joined Social Bookmarking - June 16, 2016

{"style":{"fill":{"g":255,"b":255,"r":255},"outline":{"g":0,"b":0,"r":0,"a":0.0}},"data":[{"id":"dSource","rows":[[0,3.703703703703703],[1,96.29629629629629]],"fields":[{"id":"fVariable","categories":["Joined social bookmarking","Not joined social bookmarking"],"label":"Joined Social Bookmarking","format":{"numericPattern":"###"}},{"min":3.703703703703703,"id":"fVariable1","max":96.29629629629629,"label":"Y Axis"}]}],"titles":[{"backgroundStyle":{"fill":{"g":0,"b":0,"r":0,"a":0.0},"outline":{"g":0,"b":0,"r":0,"a":0.0}},"content":["Joined Social Bookmarking"],"style":{"font":{"weight":"bold","family":"'Helvetica Neue;Helvetica;Arial;SansSerif', sans-serif","size":"12pt"},"fill":{"g":100,"b":157,"r":0},"padding":3.0},"type":"title"}],"copyright":"(C) Copyright IBM Corp. 2011","version":"6.0","size":{"height":500.0,"width":625.0},"grammar":[{"elements":[{"position":[{"field":{"$ref":"fVariable1"}},{"field":{"$ref":"fVariable"}}],"style":{"fill":{"g":178,"b":239,"r":0},"outline":{"g":100,"b":157,"r":0},"size":"75.0%"},"data":{"$ref":"dSource"},"type":"interval"}],"coordinates":{"style":{"fill":{"g":255,"b":255,"r":255},"outline":{"g":100,"b":157,"r":0}},"dimensions":[{"axis":[{"title":["Percent"],"lineStyle":{"stroke":{"width":0.6666667},"fill":{"g":100,"b":157,"r":0}},"tickStyle":{"font":{"weight":"normal","family":"sans-serif","size":"8pt"},"fill":{"g":100,"b":157,"r":0}},"titleStyle":{"font":{"weight":"bold","family":"'Helvetica Neue;Helvetica;Arial;SansSerif', sans-serif","size":"11pt"},"fill":{"g":100,"b":157,"r":0},"padding":6.0},"markStyle":{"stroke":{"width":1.3333334},"fill":{"g":100,"b":157,"r":0}}}],"scale":{"spans":[{"tickDelta":20.0,"outRange":{"min":0.0,"max":1.0}}],"padding":{"right":"5%"}}},{"axis":[{"title":["Joined Social Bookmarking"],"lineStyle":{"stroke":{"width":0.6666667},"fill":{"g":100,"b":157,"r":0}},"tickStyle":{"font":{"weight":"normal","family":"sans-serif","size":"8pt"},"fill":{"g":100,"b":157,"r":0}},"titleStyle":{"font":{"weight":"bold","family":"'Helvetica Neue;Helvetica;Arial;SansSerif', sans-serif","size":"11pt"},"fill":{"g":100,"b":157,"r":0},"padding":6.0},"markStyle":{"stroke":{"width":1.3333334},"fill":{"g":100,"b":157,"r":0}}}],"scale":{"spans":[{"tickDelta":1.0}],"padding":{"left":"5%","right":"5%"}}}]}}]}

Bar Chart  
Bar Chart - Aggregate of number of social media sites joined - June 16, 2016

{"style":{"fill":{"g":255,"b":255,"r":255},"outline":{"g":0,"b":0,"r":0,"a":0.0}},"data":[{"id":"dSource","rows":[[0,3.703703703703703],[1,37.03703703703704],[2,14.81481481481481],[3,11.11111111111111],[4,18.51851851851852],[5,3.703703703703703],[6,7.407407407407407],[7,3.703703703703703]],"fields":[{"id":"fVariable","categories":["1.00","3.00","4.00","5.00","6.00","7.00","8.00","9.00"],"label":"Aggregate of number of social media sites joined","format":{"numericPattern":"###"}},{"min":3.703703703703703,"id":"fVariable1","max":37.03703703703704,"label":"Y Axis"}]}],"titles":[{"backgroundStyle":{"fill":{"g":0,"b":0,"r":0,"a":0.0},"outline":{"g":0,"b":0,"r":0,"a":0.0}},"content":["Aggregate of number of social media sites joined"],"style":{"font":{"weight":"bold","family":"'Helvetica Neue;Helvetica;Arial;SansSerif', sans-serif","size":"12pt"},"fill":{"g":100,"b":157,"r":0},"padding":3.0},"type":"title"}],"copyright":"(C) Copyright IBM Corp. 2011","version":"6.0","size":{"height":500.0,"width":625.0},"grammar":[{"elements":[{"position":[{"field":{"$ref":"fVariable1"}},{"field":{"$ref":"fVariable"}}],"style":{"fill":{"g":178,"b":239,"r":0},"outline":{"g":100,"b":157,"r":0},"size":"75.0%"},"data":{"$ref":"dSource"},"type":"interval"}],"coordinates":{"style":{"fill":{"g":255,"b":255,"r":255},"outline":{"g":100,"b":157,"r":0}},"dimensions":[{"axis":[{"title":["Percent"],"lineStyle":{"stroke":{"width":0.6666667},"fill":{"g":100,"b":157,"r":0}},"tickStyle":{"font":{"weight":"normal","family":"sans-serif","size":"8pt"},"fill":{"g":100,"b":157,"r":0}},"titleStyle":{"font":{"weight":"bold","family":"'Helvetica Neue;Helvetica;Arial;SansSerif', sans-serif","size":"11pt"},"fill":{"g":100,"b":157,"r":0},"padding":6.0},"markStyle":{"stroke":{"width":1.3333334},"fill":{"g":100,"b":157,"r":0}}}],"scale":{"spans":[{"tickDelta":10.0,"outRange":{"min":0.0,"max":1.0}}],"padding":{"right":"5%"}}},{"axis":[{"title":["Aggregate of number of social media sites joined"],"lineStyle":{"stroke":{"width":0.6666667},"fill":{"g":100,"b":157,"r":0}},"tickStyle":{"font":{"weight":"normal","family":"sans-serif","size":"8pt"},"fill":{"g":100,"b":157,"r":0}},"titleStyle":{"font":{"weight":"bold","family":"'Helvetica Neue;Helvetica;Arial;SansSerif', sans-serif","size":"11pt"},"fill":{"g":100,"b":157,"r":0},"padding":6.0},"markStyle":{"stroke":{"width":1.3333334},"fill":{"g":100,"b":157,"r":0}}}],"scale":{"spans":[{"tickDelta":1.0}],"padding":{"left":"5%","right":"5%"}}}]}}]}

Bar Chart  
Bar Chart - Posted Facebook - June 16, 2016

{"style":{"fill":{"g":255,"b":255,"r":255},"outline":{"g":0,"b":0,"r":0,"a":0.0}},"data":[{"id":"dSource","rows":[[0,81.48148148148148],[1,18.51851851851852]],"fields":[{"id":"fVariable","categories":["Posted Facebook","Not posted Facebook"],"label":"Posted Facebook","format":{"numericPattern":"###"}},{"min":18.51851851851852,"id":"fVariable1","max":81.48148148148148,"label":"Y Axis"}]}],"titles":[{"backgroundStyle":{"fill":{"g":0,"b":0,"r":0,"a":0.0},"outline":{"g":0,"b":0,"r":0,"a":0.0}},"content":["Posted Facebook"],"style":{"font":{"weight":"bold","family":"'Helvetica Neue;Helvetica;Arial;SansSerif', sans-serif","size":"12pt"},"fill":{"g":100,"b":157,"r":0},"padding":3.0},"type":"title"}],"copyright":"(C) Copyright IBM Corp. 2011","version":"6.0","size":{"height":500.0,"width":625.0},"grammar":[{"elements":[{"position":[{"field":{"$ref":"fVariable1"}},{"field":{"$ref":"fVariable"}}],"style":{"fill":{"g":178,"b":239,"r":0},"outline":{"g":100,"b":157,"r":0},"size":"75.0%"},"data":{"$ref":"dSource"},"type":"interval"}],"coordinates":{"style":{"fill":{"g":255,"b":255,"r":255},"outline":{"g":100,"b":157,"r":0}},"dimensions":[{"axis":[{"title":["Percent"],"lineStyle":{"stroke":{"width":0.6666667},"fill":{"g":100,"b":157,"r":0}},"tickStyle":{"font":{"weight":"normal","family":"sans-serif","size":"8pt"},"fill":{"g":100,"b":157,"r":0}},"titleStyle":{"font":{"weight":"bold","family":"'Helvetica Neue;Helvetica;Arial;SansSerif', sans-serif","size":"11pt"},"fill":{"g":100,"b":157,"r":0},"padding":6.0},"markStyle":{"stroke":{"width":1.3333334},"fill":{"g":100,"b":157,"r":0}}}],"scale":{"spans":[{"tickDelta":20.0,"outRange":{"min":0.0,"max":1.0}}],"padding":{"right":"5%"}}},{"axis":[{"title":["Posted Facebook"],"lineStyle":{"stroke":{"width":0.6666667},"fill":{"g":100,"b":157,"r":0}},"tickStyle":{"font":{"weight":"normal","family":"sans-serif","size":"8pt"},"fill":{"g":100,"b":157,"r":0}},"titleStyle":{"font":{"weight":"bold","family":"'Helvetica Neue;Helvetica;Arial;SansSerif', sans-serif","size":"11pt"},"fill":{"g":100,"b":157,"r":0},"padding":6.0},"markStyle":{"stroke":{"width":1.3333334},"fill":{"g":100,"b":157,"r":0}}}],"scale":{"spans":[{"tickDelta":1.0}],"padding":{"left":"5%","right":"5%"}}}]}}]}

Bar Chart  
Bar Chart - Posted Twitter - June 16, 2016

{"style":{"fill":{"g":255,"b":255,"r":255},"outline":{"g":0,"b":0,"r":0,"a":0.0}},"data":[{"id":"dSource","rows":[[0,70.37037037037037],[1,29.62962962962963]],"fields":[{"id":"fVariable","categories":["Posted Twitter","Not posted Twitter"],"label":"Posted Twitter","format":{"numericPattern":"###"}},{"min":29.62962962962963,"id":"fVariable1","max":70.37037037037037,"label":"Y Axis"}]}],"titles":[{"backgroundStyle":{"fill":{"g":0,"b":0,"r":0,"a":0.0},"outline":{"g":0,"b":0,"r":0,"a":0.0}},"content":["Posted Twitter"],"style":{"font":{"weight":"bold","family":"'Helvetica Neue;Helvetica;Arial;SansSerif', sans-serif","size":"12pt"},"fill":{"g":100,"b":157,"r":0},"padding":3.0},"type":"title"}],"copyright":"(C) Copyright IBM Corp. 2011","version":"6.0","size":{"height":500.0,"width":625.0},"grammar":[{"elements":[{"position":[{"field":{"$ref":"fVariable1"}},{"field":{"$ref":"fVariable"}}],"style":{"fill":{"g":178,"b":239,"r":0},"outline":{"g":100,"b":157,"r":0},"size":"75.0%"},"data":{"$ref":"dSource"},"type":"interval"}],"coordinates":{"style":{"fill":{"g":255,"b":255,"r":255},"outline":{"g":100,"b":157,"r":0}},"dimensions":[{"axis":[{"title":["Percent"],"lineStyle":{"stroke":{"width":0.6666667},"fill":{"g":100,"b":157,"r":0}},"tickStyle":{"font":{"weight":"normal","family":"sans-serif","size":"8pt"},"fill":{"g":100,"b":157,"r":0}},"titleStyle":{"font":{"weight":"bold","family":"'Helvetica Neue;Helvetica;Arial;SansSerif', sans-serif","size":"11pt"},"fill":{"g":100,"b":157,"r":0},"padding":6.0},"markStyle":{"stroke":{"width":1.3333334},"fill":{"g":100,"b":157,"r":0}}}],"scale":{"spans":[{"tickDelta":20.0,"outRange":{"min":0.0,"max":1.0}}],"padding":{"right":"5%"}}},{"axis":[{"title":["Posted Twitter"],"lineStyle":{"stroke":{"width":0.6666667},"fill":{"g":100,"b":157,"r":0}},"tickStyle":{"font":{"weight":"normal","family":"sans-serif","size":"8pt"},"fill":{"g":100,"b":157,"r":0}},"titleStyle":{"font":{"weight":"bold","family":"'Helvetica Neue;Helvetica;Arial;SansSerif', sans-serif","size":"11pt"},"fill":{"g":100,"b":157,"r":0},"padding":6.0},"markStyle":{"stroke":{"width":1.3333334},"fill":{"g":100,"b":157,"r":0}}}],"scale":{"spans":[{"tickDelta":1.0}],"padding":{"left":"5%","right":"5%"}}}]}}]}

Bar Chart  
Bar Chart - Posted Pinterest - June 16, 2016

{"style":{"fill":{"g":255,"b":255,"r":255},"outline":{"g":0,"b":0,"r":0,"a":0.0}},"data":[{"id":"dSource","rows":[[0,37.03703703703704],[1,62.96296296296296]],"fields":[{"id":"fVariable","categories":["Posted Pinterest","Not posted Pinterest"],"label":"Posted Pinterest","format":{"numericPattern":"###"}},{"min":37.03703703703704,"id":"fVariable1","max":62.96296296296296,"label":"Y Axis"}]}],"titles":[{"backgroundStyle":{"fill":{"g":0,"b":0,"r":0,"a":0.0},"outline":{"g":0,"b":0,"r":0,"a":0.0}},"content":["Posted Pinterest"],"style":{"font":{"weight":"bold","family":"'Helvetica Neue;Helvetica;Arial;SansSerif', sans-serif","size":"12pt"},"fill":{"g":100,"b":157,"r":0},"padding":3.0},"type":"title"}],"copyright":"(C) Copyright IBM Corp. 2011","version":"6.0","size":{"height":500.0,"width":625.0},"grammar":[{"elements":[{"position":[{"field":{"$ref":"fVariable1"}},{"field":{"$ref":"fVariable"}}],"style":{"fill":{"g":178,"b":239,"r":0},"outline":{"g":100,"b":157,"r":0},"size":"75.0%"},"data":{"$ref":"dSource"},"type":"interval"}],"coordinates":{"style":{"fill":{"g":255,"b":255,"r":255},"outline":{"g":100,"b":157,"r":0}},"dimensions":[{"axis":[{"title":["Percent"],"lineStyle":{"stroke":{"width":0.6666667},"fill":{"g":100,"b":157,"r":0}},"tickStyle":{"font":{"weight":"normal","family":"sans-serif","size":"8pt"},"fill":{"g":100,"b":157,"r":0}},"titleStyle":{"font":{"weight":"bold","family":"'Helvetica Neue;Helvetica;Arial;SansSerif', sans-serif","size":"11pt"},"fill":{"g":100,"b":157,"r":0},"padding":6.0},"markStyle":{"stroke":{"width":1.3333334},"fill":{"g":100,"b":157,"r":0}}}],"scale":{"spans":[{"tickDelta":20.0,"outRange":{"min":0.0,"max":1.0}}],"padding":{"right":"5%"}}},{"axis":[{"title":["Posted Pinterest"],"lineStyle":{"stroke":{"width":0.6666667},"fill":{"g":100,"b":157,"r":0}},"tickStyle":{"font":{"weight":"normal","family":"sans-serif","size":"8pt"},"fill":{"g":100,"b":157,"r":0}},"titleStyle":{"font":{"weight":"bold","family":"'Helvetica Neue;Helvetica;Arial;SansSerif', sans-serif","size":"11pt"},"fill":{"g":100,"b":157,"r":0},"padding":6.0},"markStyle":{"stroke":{"width":1.3333334},"fill":{"g":100,"b":157,"r":0}}}],"scale":{"spans":[{"tickDelta":1.0}],"padding":{"left":"5%","right":"5%"}}}]}}]}

Bar Chart  
Bar Chart - Posted LinkedIN - June 16, 2016

{"style":{"fill":{"g":255,"b":255,"r":255},"outline":{"g":0,"b":0,"r":0,"a":0.0}},"data":[{"id":"dSource","rows":[[0,55.55555555555556],[1,44.44444444444444]],"fields":[{"id":"fVariable","categories":["Posted LinkedIn","Not posted LinkedIn"],"label":"Posted LinkedIN","format":{"numericPattern":"###"}},{"min":44.44444444444444,"id":"fVariable1","max":55.55555555555556,"label":"Y Axis"}]}],"titles":[{"backgroundStyle":{"fill":{"g":0,"b":0,"r":0,"a":0.0},"outline":{"g":0,"b":0,"r":0,"a":0.0}},"content":["Posted LinkedIN"],"style":{"font":{"weight":"bold","family":"'Helvetica Neue;Helvetica;Arial;SansSerif', sans-serif","size":"12pt"},"fill":{"g":100,"b":157,"r":0},"padding":3.0},"type":"title"}],"copyright":"(C) Copyright IBM Corp. 2011","version":"6.0","size":{"height":500.0,"width":625.0},"grammar":[{"elements":[{"position":[{"field":{"$ref":"fVariable1"}},{"field":{"$ref":"fVariable"}}],"style":{"fill":{"g":178,"b":239,"r":0},"outline":{"g":100,"b":157,"r":0},"size":"75.0%"},"data":{"$ref":"dSource"},"type":"interval"}],"coordinates":{"style":{"fill":{"g":255,"b":255,"r":255},"outline":{"g":100,"b":157,"r":0}},"dimensions":[{"axis":[{"title":["Percent"],"lineStyle":{"stroke":{"width":0.6666667},"fill":{"g":100,"b":157,"r":0}},"tickStyle":{"font":{"weight":"normal","family":"sans-serif","size":"8pt"},"fill":{"g":100,"b":157,"r":0}},"titleStyle":{"font":{"weight":"bold","family":"'Helvetica Neue;Helvetica;Arial;SansSerif', sans-serif","size":"11pt"},"fill":{"g":100,"b":157,"r":0},"padding":6.0},"markStyle":{"stroke":{"width":1.3333334},"fill":{"g":100,"b":157,"r":0}}}],"scale":{"spans":[{"tickDelta":10.0,"outRange":{"min":0.0,"max":1.0}}],"padding":{"right":"5%"}}},{"axis":[{"title":["Posted LinkedIN"],"lineStyle":{"stroke":{"width":0.6666667},"fill":{"g":100,"b":157,"r":0}},"tickStyle":{"font":{"weight":"normal","family":"sans-serif","size":"8pt"},"fill":{"g":100,"b":157,"r":0}},"titleStyle":{"font":{"weight":"bold","family":"'Helvetica Neue;Helvetica;Arial;SansSerif', sans-serif","size":"11pt"},"fill":{"g":100,"b":157,"r":0},"padding":6.0},"markStyle":{"stroke":{"width":1.3333334},"fill":{"g":100,"b":157,"r":0}}}],"scale":{"spans":[{"tickDelta":1.0}],"padding":{"left":"5%","right":"5%"}}}]}}]}

Bar Chart  
Bar Chart - Posted Foursquare - June 16, 2016

{"style":{"fill":{"g":255,"b":255,"r":255},"outline":{"g":0,"b":0,"r":0,"a":0.0}},"data":[{"id":"dSource","rows":[[0,22.22222222222222],[1,77.77777777777779]],"fields":[{"id":"fVariable","categories":["Posted Foursquare","Not posted Foursquare"],"label":"Posted Foursquare","format":{"numericPattern":"###"}},{"min":22.22222222222222,"id":"fVariable1","max":77.77777777777779,"label":"Y Axis"}]}],"titles":[{"backgroundStyle":{"fill":{"g":0,"b":0,"r":0,"a":0.0},"outline":{"g":0,"b":0,"r":0,"a":0.0}},"content":["Posted Foursquare"],"style":{"font":{"weight":"bold","family":"'Helvetica Neue;Helvetica;Arial;SansSerif', sans-serif","size":"12pt"},"fill":{"g":100,"b":157,"r":0},"padding":3.0},"type":"title"}],"copyright":"(C) Copyright IBM Corp. 2011","version":"6.0","size":{"height":500.0,"width":625.0},"grammar":[{"elements":[{"position":[{"field":{"$ref":"fVariable1"}},{"field":{"$ref":"fVariable"}}],"style":{"fill":{"g":178,"b":239,"r":0},"outline":{"g":100,"b":157,"r":0},"size":"75.0%"},"data":{"$ref":"dSource"},"type":"interval"}],"coordinates":{"style":{"fill":{"g":255,"b":255,"r":255},"outline":{"g":100,"b":157,"r":0}},"dimensions":[{"axis":[{"title":["Percent"],"lineStyle":{"stroke":{"width":0.6666667},"fill":{"g":100,"b":157,"r":0}},"tickStyle":{"font":{"weight":"normal","family":"sans-serif","size":"8pt"},"fill":{"g":100,"b":157,"r":0}},"titleStyle":{"font":{"weight":"bold","family":"'Helvetica Neue;Helvetica;Arial;SansSerif', sans-serif","size":"11pt"},"fill":{"g":100,"b":157,"r":0},"padding":6.0},"markStyle":{"stroke":{"width":1.3333334},"fill":{"g":100,"b":157,"r":0}}}],"scale":{"spans":[{"tickDelta":20.0,"outRange":{"min":0.0,"max":1.0}}],"padding":{"right":"5%"}}},{"axis":[{"title":["Posted Foursquare"],"lineStyle":{"stroke":{"width":0.6666667},"fill":{"g":100,"b":157,"r":0}},"tickStyle":{"font":{"weight":"normal","family":"sans-serif","size":"8pt"},"fill":{"g":100,"b":157,"r":0}},"titleStyle":{"font":{"weight":"bold","family":"'Helvetica Neue;Helvetica;Arial;SansSerif', sans-serif","size":"11pt"},"fill":{"g":100,"b":157,"r":0},"padding":6.0},"markStyle":{"stroke":{"width":1.3333334},"fill":{"g":100,"b":157,"r":0}}}],"scale":{"spans":[{"tickDelta":1.0}],"padding":{"left":"5%","right":"5%"}}}]}}]}

Bar Chart  
Bar Chart - Posted Tumblr - June 16, 2016

{"style":{"fill":{"g":255,"b":255,"r":255},"outline":{"g":0,"b":0,"r":0,"a":0.0}},"data":[{"id":"dSource","rows":[[0,18.51851851851852],[1,81.48148148148148]],"fields":[{"id":"fVariable","categories":["Posted Tumblr","Not posted Tumblr"],"label":"Posted Tumblr","format":{"numericPattern":"###"}},{"min":18.51851851851852,"id":"fVariable1","max":81.48148148148148,"label":"Y Axis"}]}],"titles":[{"backgroundStyle":{"fill":{"g":0,"b":0,"r":0,"a":0.0},"outline":{"g":0,"b":0,"r":0,"a":0.0}},"content":["Posted Tumblr"],"style":{"font":{"weight":"bold","family":"'Helvetica Neue;Helvetica;Arial;SansSerif', sans-serif","size":"12pt"},"fill":{"g":100,"b":157,"r":0},"padding":3.0},"type":"title"}],"copyright":"(C) Copyright IBM Corp. 2011","version":"6.0","size":{"height":500.0,"width":625.0},"grammar":[{"elements":[{"position":[{"field":{"$ref":"fVariable1"}},{"field":{"$ref":"fVariable"}}],"style":{"fill":{"g":178,"b":239,"r":0},"outline":{"g":100,"b":157,"r":0},"size":"75.0%"},"data":{"$ref":"dSource"},"type":"interval"}],"coordinates":{"style":{"fill":{"g":255,"b":255,"r":255},"outline":{"g":100,"b":157,"r":0}},"dimensions":[{"axis":[{"title":["Percent"],"lineStyle":{"stroke":{"width":0.6666667},"fill":{"g":100,"b":157,"r":0}},"tickStyle":{"font":{"weight":"normal","family":"sans-serif","size":"8pt"},"fill":{"g":100,"b":157,"r":0}},"titleStyle":{"font":{"weight":"bold","family":"'Helvetica Neue;Helvetica;Arial;SansSerif', sans-serif","size":"11pt"},"fill":{"g":100,"b":157,"r":0},"padding":6.0},"markStyle":{"stroke":{"width":1.3333334},"fill":{"g":100,"b":157,"r":0}}}],"scale":{"spans":[{"tickDelta":20.0,"outRange":{"min":0.0,"max":1.0}}],"padding":{"right":"5%"}}},{"axis":[{"title":["Posted Tumblr"],"lineStyle":{"stroke":{"width":0.6666667},"fill":{"g":100,"b":157,"r":0}},"tickStyle":{"font":{"weight":"normal","family":"sans-serif","size":"8pt"},"fill":{"g":100,"b":157,"r":0}},"titleStyle":{"font":{"weight":"bold","family":"'Helvetica Neue;Helvetica;Arial;SansSerif', sans-serif","size":"11pt"},"fill":{"g":100,"b":157,"r":0},"padding":6.0},"markStyle":{"stroke":{"width":1.3333334},"fill":{"g":100,"b":157,"r":0}}}],"scale":{"spans":[{"tickDelta":1.0}],"padding":{"left":"5%","right":"5%"}}}]}}]}

Bar Chart  
Bar Chart - Posted GooglePlus - June 16, 2016

{"style":{"fill":{"g":255,"b":255,"r":255},"outline":{"g":0,"b":0,"r":0,"a":0.0}},"data":[{"id":"dSource","rows":[[0,85.18518518518519],[1,14.81481481481481]],"fields":[{"id":"fVariable","categories":["Posted Google Plus","Not posted Google Plus"],"label":"Posted GooglePlus","format":{"numericPattern":"###"}},{"min":14.81481481481481,"id":"fVariable1","max":85.18518518518519,"label":"Y Axis"}]}],"titles":[{"backgroundStyle":{"fill":{"g":0,"b":0,"r":0,"a":0.0},"outline":{"g":0,"b":0,"r":0,"a":0.0}},"content":["Posted GooglePlus"],"style":{"font":{"weight":"bold","family":"'Helvetica Neue;Helvetica;Arial;SansSerif', sans-serif","size":"12pt"},"fill":{"g":100,"b":157,"r":0},"padding":3.0},"type":"title"}],"copyright":"(C) Copyright IBM Corp. 2011","version":"6.0","size":{"height":500.0,"width":625.0},"grammar":[{"elements":[{"position":[{"field":{"$ref":"fVariable1"}},{"field":{"$ref":"fVariable"}}],"style":{"fill":{"g":178,"b":239,"r":0},"outline":{"g":100,"b":157,"r":0},"size":"75.0%"},"data":{"$ref":"dSource"},"type":"interval"}],"coordinates":{"style":{"fill":{"g":255,"b":255,"r":255},"outline":{"g":100,"b":157,"r":0}},"dimensions":[{"axis":[{"title":["Percent"],"lineStyle":{"stroke":{"width":0.6666667},"fill":{"g":100,"b":157,"r":0}},"tickStyle":{"font":{"weight":"normal","family":"sans-serif","size":"8pt"},"fill":{"g":100,"b":157,"r":0}},"titleStyle":{"font":{"weight":"bold","family":"'Helvetica Neue;Helvetica;Arial;SansSerif', sans-serif","size":"11pt"},"fill":{"g":100,"b":157,"r":0},"padding":6.0},"markStyle":{"stroke":{"width":1.3333334},"fill":{"g":100,"b":157,"r":0}}}],"scale":{"spans":[{"tickDelta":20.0,"outRange":{"min":0.0,"max":1.0}}],"padding":{"right":"5%"}}},{"axis":[{"title":["Posted GooglePlus"],"lineStyle":{"stroke":{"width":0.6666667},"fill":{"g":100,"b":157,"r":0}},"tickStyle":{"font":{"weight":"normal","family":"sans-serif","size":"8pt"},"fill":{"g":100,"b":157,"r":0}},"titleStyle":{"font":{"weight":"bold","family":"'Helvetica Neue;Helvetica;Arial;SansSerif', sans-serif","size":"11pt"},"fill":{"g":100,"b":157,"r":0},"padding":6.0},"markStyle":{"stroke":{"width":1.3333334},"fill":{"g":100,"b":157,"r":0}}}],"scale":{"spans":[{"tickDelta":1.0}],"padding":{"left":"5%","right":"5%"}}}]}}]}

Bar Chart  
Bar Chart - Posted Youtube - June 16, 2016

{"style":{"fill":{"g":255,"b":255,"r":255},"outline":{"g":0,"b":0,"r":0,"a":0.0}},"data":[{"id":"dSource","rows":[[0,44.44444444444444],[1,55.55555555555556]],"fields":[{"id":"fVariable","categories":["Posted YouTube","Not posted Youtube"],"label":"Posted Youtube","format":{"numericPattern":"###"}},{"min":44.44444444444444,"id":"fVariable1","max":55.55555555555556,"label":"Y Axis"}]}],"titles":[{"backgroundStyle":{"fill":{"g":0,"b":0,"r":0,"a":0.0},"outline":{"g":0,"b":0,"r":0,"a":0.0}},"content":["Posted Youtube"],"style":{"font":{"weight":"bold","family":"'Helvetica Neue;Helvetica;Arial;SansSerif', sans-serif","size":"12pt"},"fill":{"g":100,"b":157,"r":0},"padding":3.0},"type":"title"}],"copyright":"(C) Copyright IBM Corp. 2011","version":"6.0","size":{"height":500.0,"width":625.0},"grammar":[{"elements":[{"position":[{"field":{"$ref":"fVariable1"}},{"field":{"$ref":"fVariable"}}],"style":{"fill":{"g":178,"b":239,"r":0},"outline":{"g":100,"b":157,"r":0},"size":"75.0%"},"data":{"$ref":"dSource"},"type":"interval"}],"coordinates":{"style":{"fill":{"g":255,"b":255,"r":255},"outline":{"g":100,"b":157,"r":0}},"dimensions":[{"axis":[{"title":["Percent"],"lineStyle":{"stroke":{"width":0.6666667},"fill":{"g":100,"b":157,"r":0}},"tickStyle":{"font":{"weight":"normal","family":"sans-serif","size":"8pt"},"fill":{"g":100,"b":157,"r":0}},"titleStyle":{"font":{"weight":"bold","family":"'Helvetica Neue;Helvetica;Arial;SansSerif', sans-serif","size":"11pt"},"fill":{"g":100,"b":157,"r":0},"padding":6.0},"markStyle":{"stroke":{"width":1.3333334},"fill":{"g":100,"b":157,"r":0}}}],"scale":{"spans":[{"tickDelta":10.0,"outRange":{"min":0.0,"max":1.0}}],"padding":{"right":"5%"}}},{"axis":[{"title":["Posted Youtube"],"lineStyle":{"stroke":{"width":0.6666667},"fill":{"g":100,"b":157,"r":0}},"tickStyle":{"font":{"weight":"normal","family":"sans-serif","size":"8pt"},"fill":{"g":100,"b":157,"r":0}},"titleStyle":{"font":{"weight":"bold","family":"'Helvetica Neue;Helvetica;Arial;SansSerif', sans-serif","size":"11pt"},"fill":{"g":100,"b":157,"r":0},"padding":6.0},"markStyle":{"stroke":{"width":1.3333334},"fill":{"g":100,"b":157,"r":0}}}],"scale":{"spans":[{"tickDelta":1.0}],"padding":{"left":"5%","right":"5%"}}}]}}]}

Bar Chart  
Bar Chart - Posted MySpace - June 16, 2016

{"style":{"fill":{"g":255,"b":255,"r":255},"outline":{"g":0,"b":0,"r":0,"a":0.0}},"data":[{"id":"dSource","rows":[[0,18.51851851851852],[1,81.48148148148148]],"fields":[{"id":"fVariable","categories":["Posted MySpace","Not posted MySpace"],"label":"Posted MySpace","format":{"numericPattern":"###"}},{"min":18.51851851851852,"id":"fVariable1","max":81.48148148148148,"label":"Y Axis"}]}],"titles":[{"backgroundStyle":{"fill":{"g":0,"b":0,"r":0,"a":0.0},"outline":{"g":0,"b":0,"r":0,"a":0.0}},"content":["Posted MySpace"],"style":{"font":{"weight":"bold","family":"'Helvetica Neue;Helvetica;Arial;SansSerif', sans-serif","size":"12pt"},"fill":{"g":100,"b":157,"r":0},"padding":3.0},"type":"title"}],"copyright":"(C) Copyright IBM Corp. 2011","version":"6.0","size":{"height":500.0,"width":625.0},"grammar":[{"elements":[{"position":[{"field":{"$ref":"fVariable1"}},{"field":{"$ref":"fVariable"}}],"style":{"fill":{"g":178,"b":239,"r":0},"outline":{"g":100,"b":157,"r":0},"size":"75.0%"},"data":{"$ref":"dSource"},"type":"interval"}],"coordinates":{"style":{"fill":{"g":255,"b":255,"r":255},"outline":{"g":100,"b":157,"r":0}},"dimensions":[{"axis":[{"title":["Percent"],"lineStyle":{"stroke":{"width":0.6666667},"fill":{"g":100,"b":157,"r":0}},"tickStyle":{"font":{"weight":"normal","family":"sans-serif","size":"8pt"},"fill":{"g":100,"b":157,"r":0}},"titleStyle":{"font":{"weight":"bold","family":"'Helvetica Neue;Helvetica;Arial;SansSerif', sans-serif","size":"11pt"},"fill":{"g":100,"b":157,"r":0},"padding":6.0},"markStyle":{"stroke":{"width":1.3333334},"fill":{"g":100,"b":157,"r":0}}}],"scale":{"spans":[{"tickDelta":20.0,"outRange":{"min":0.0,"max":1.0}}],"padding":{"right":"5%"}}},{"axis":[{"title":["Posted MySpace"],"lineStyle":{"stroke":{"width":0.6666667},"fill":{"g":100,"b":157,"r":0}},"tickStyle":{"font":{"weight":"normal","family":"sans-serif","size":"8pt"},"fill":{"g":100,"b":157,"r":0}},"titleStyle":{"font":{"weight":"bold","family":"'Helvetica Neue;Helvetica;Arial;SansSerif', sans-serif","size":"11pt"},"fill":{"g":100,"b":157,"r":0},"padding":6.0},"markStyle":{"stroke":{"width":1.3333334},"fill":{"g":100,"b":157,"r":0}}}],"scale":{"spans":[{"tickDelta":1.0}],"padding":{"left":"5%","right":"5%"}}}]}}]}

Bar Chart  
Bar Chart - Posted Blogs - June 16, 2016

{"style":{"fill":{"g":255,"b":255,"r":255},"outline":{"g":0,"b":0,"r":0,"a":0.0}},"data":[{"id":"dSource","rows":[[0,37.03703703703704],[1,62.96296296296296]],"fields":[{"id":"fVariable","categories":["Posted blogs","Not posted blogs"],"label":"Posted Blogs","format":{"numericPattern":"###"}},{"min":37.03703703703704,"id":"fVariable1","max":62.96296296296296,"label":"Y Axis"}]}],"titles":[{"backgroundStyle":{"fill":{"g":0,"b":0,"r":0,"a":0.0},"outline":{"g":0,"b":0,"r":0,"a":0.0}},"content":["Posted Blogs"],"style":{"font":{"weight":"bold","family":"'Helvetica Neue;Helvetica;Arial;SansSerif', sans-serif","size":"12pt"},"fill":{"g":100,"b":157,"r":0},"padding":3.0},"type":"title"}],"copyright":"(C) Copyright IBM Corp. 2011","version":"6.0","size":{"height":500.0,"width":625.0},"grammar":[{"elements":[{"position":[{"field":{"$ref":"fVariable1"}},{"field":{"$ref":"fVariable"}}],"style":{"fill":{"g":178,"b":239,"r":0},"outline":{"g":100,"b":157,"r":0},"size":"75.0%"},"data":{"$ref":"dSource"},"type":"interval"}],"coordinates":{"style":{"fill":{"g":255,"b":255,"r":255},"outline":{"g":100,"b":157,"r":0}},"dimensions":[{"axis":[{"title":["Percent"],"lineStyle":{"stroke":{"width":0.6666667},"fill":{"g":100,"b":157,"r":0}},"tickStyle":{"font":{"weight":"normal","family":"sans-serif","size":"8pt"},"fill":{"g":100,"b":157,"r":0}},"titleStyle":{"font":{"weight":"bold","family":"'Helvetica Neue;Helvetica;Arial;SansSerif', sans-serif","size":"11pt"},"fill":{"g":100,"b":157,"r":0},"padding":6.0},"markStyle":{"stroke":{"width":1.3333334},"fill":{"g":100,"b":157,"r":0}}}],"scale":{"spans":[{"tickDelta":20.0,"outRange":{"min":0.0,"max":1.0}}],"padding":{"right":"5%"}}},{"axis":[{"title":["Posted Blogs"],"lineStyle":{"stroke":{"width":0.6666667},"fill":{"g":100,"b":157,"r":0}},"tickStyle":{"font":{"weight":"normal","family":"sans-serif","size":"8pt"},"fill":{"g":100,"b":157,"r":0}},"titleStyle":{"font":{"weight":"bold","family":"'Helvetica Neue;Helvetica;Arial;SansSerif', sans-serif","size":"11pt"},"fill":{"g":100,"b":157,"r":0},"padding":6.0},"markStyle":{"stroke":{"width":1.3333334},"fill":{"g":100,"b":157,"r":0}}}],"scale":{"spans":[{"tickDelta":1.0}],"padding":{"left":"5%","right":"5%"}}}]}}]}

Bar Chart  
Bar Chart - Aggregate of number of social media sites posted - June 16, 2016

{"style":{"fill":{"g":255,"b":255,"r":255},"outline":{"g":0,"b":0,"r":0,"a":0.0}},"data":[{"id":"dSource","rows":[[0,3.703703703703703],[1,22.22222222222222],[2,29.62962962962963],[3,7.407407407407407],[4,22.22222222222222],[5,11.11111111111111],[6,3.703703703703703]],"fields":[{"id":"fVariable","categories":["2.00","3.00","4.00","5.00","6.00","7.00","8.00"],"label":"Aggregate of number of social media sites posted","format":{"numericPattern":"###"}},{"min":3.703703703703703,"id":"fVariable1","max":29.62962962962963,"label":"Y Axis"}]}],"titles":[{"backgroundStyle":{"fill":{"g":0,"b":0,"r":0,"a":0.0},"outline":{"g":0,"b":0,"r":0,"a":0.0}},"content":["Aggregate of number of social media sites posted"],"style":{"font":{"weight":"bold","family":"'Helvetica Neue;Helvetica;Arial;SansSerif', sans-serif","size":"12pt"},"fill":{"g":100,"b":157,"r":0},"padding":3.0},"type":"title"}],"copyright":"(C) Copyright IBM Corp. 2011","version":"6.0","size":{"height":500.0,"width":625.0},"grammar":[{"elements":[{"position":[{"field":{"$ref":"fVariable1"}},{"field":{"$ref":"fVariable"}}],"style":{"fill":{"g":178,"b":239,"r":0},"outline":{"g":100,"b":157,"r":0},"size":"75.0%"},"data":{"$ref":"dSource"},"type":"interval"}],"coordinates":{"style":{"fill":{"g":255,"b":255,"r":255},"outline":{"g":100,"b":157,"r":0}},"dimensions":[{"axis":[{"title":["Percent"],"lineStyle":{"stroke":{"width":0.6666667},"fill":{"g":100,"b":157,"r":0}},"tickStyle":{"font":{"weight":"normal","family":"sans-serif","size":"8pt"},"fill":{"g":100,"b":157,"r":0}},"titleStyle":{"font":{"weight":"bold","family":"'Helvetica Neue;Helvetica;Arial;SansSerif', sans-serif","size":"11pt"},"fill":{"g":100,"b":157,"r":0},"padding":6.0},"markStyle":{"stroke":{"width":1.3333334},"fill":{"g":100,"b":157,"r":0}}}],"scale":{"spans":[{"tickDelta":10.0,"outRange":{"min":0.0,"max":1.0}}],"padding":{"right":"5%"}}},{"axis":[{"title":["Aggregate of number of social media sites posted"],"lineStyle":{"stroke":{"width":0.6666667},"fill":{"g":100,"b":157,"r":0}},"tickStyle":{"font":{"weight":"normal","family":"sans-serif","size":"8pt"},"fill":{"g":100,"b":157,"r":0}},"titleStyle":{"font":{"weight":"bold","family":"'Helvetica Neue;Helvetica;Arial;SansSerif', sans-serif","size":"11pt"},"fill":{"g":100,"b":157,"r":0},"padding":6.0},"markStyle":{"stroke":{"width":1.3333334},"fill":{"g":100,"b":157,"r":0}}}],"scale":{"spans":[{"tickDelta":1.0}],"padding":{"left":"5%","right":"5%"}}}]}}]}

Bar Chart  
Bar Chart - Frequency of visits to social media sites - June 16, 2016

{"style":{"fill":{"g":255,"b":255,"r":255},"outline":{"g":0,"b":0,"r":0,"a":0.0}},"data":[{"id":"dSource","rows":[[0,7.407407407407407],[1,7.407407407407407],[2,11.11111111111111],[3,11.11111111111111],[4,25.92592592592592],[5,37.03703703703704]],"fields":[{"id":"fVariable","categories":["Never","Practically never","Once in a while","Fairly often","Very often","Almost always"],"label":"Frequency of visits to social media sites","format":{"numericPattern":"###"}},{"min":7.407407407407407,"id":"fVariable1","max":37.03703703703704,"label":"Y Axis"}]}],"titles":[{"backgroundStyle":{"fill":{"g":0,"b":0,"r":0,"a":0.0},"outline":{"g":0,"b":0,"r":0,"a":0.0}},"content":["Frequency of visits to social media sites"],"style":{"font":{"weight":"bold","family":"'Helvetica Neue;Helvetica;Arial;SansSerif', sans-serif","size":"12pt"},"fill":{"g":100,"b":157,"r":0},"padding":3.0},"type":"title"}],"copyright":"(C) Copyright IBM Corp. 2011","version":"6.0","size":{"height":500.0,"width":625.0},"grammar":[{"elements":[{"position":[{"field":{"$ref":"fVariable1"}},{"field":{"$ref":"fVariable"}}],"style":{"fill":{"g":178,"b":239,"r":0},"outline":{"g":100,"b":157,"r":0},"size":"75.0%"},"data":{"$ref":"dSource"},"type":"interval"}],"coordinates":{"style":{"fill":{"g":255,"b":255,"r":255},"outline":{"g":100,"b":157,"r":0}},"dimensions":[{"axis":[{"title":["Percent"],"lineStyle":{"stroke":{"width":0.6666667},"fill":{"g":100,"b":157,"r":0}},"tickStyle":{"font":{"weight":"normal","family":"sans-serif","size":"8pt"},"fill":{"g":100,"b":157,"r":0}},"titleStyle":{"font":{"weight":"bold","family":"'Helvetica Neue;Helvetica;Arial;SansSerif', sans-serif","size":"11pt"},"fill":{"g":100,"b":157,"r":0},"padding":6.0},"markStyle":{"stroke":{"width":1.3333334},"fill":{"g":100,"b":157,"r":0}}}],"scale":{"spans":[{"tickDelta":10.0,"outRange":{"min":0.0,"max":1.0}}],"padding":{"right":"5%"}}},{"axis":[{"title":["Frequency of visits to social media sites"],"lineStyle":{"stroke":{"width":0.6666667},"fill":{"g":100,"b":157,"r":0}},"tickStyle":{"font":{"weight":"normal","family":"sans-serif","size":"8pt"},"fill":{"g":100,"b":157,"r":0}},"titleStyle":{"font":{"weight":"bold","family":"'Helvetica Neue;Helvetica;Arial;SansSerif', sans-serif","size":"11pt"},"fill":{"g":100,"b":157,"r":0},"padding":6.0},"markStyle":{"stroke":{"width":1.3333334},"fill":{"g":100,"b":157,"r":0}}}],"scale":{"spans":[{"tickDelta":1.0}],"padding":{"left":"5%","right":"5%"}}}]}}]}

Bar Chart  
Bar Chart - Frequency of posting to social media sites - June 16, 2016

{"style":{"fill":{"g":255,"b":255,"r":255},"outline":{"g":0,"b":0,"r":0,"a":0.0}},"data":[{"id":"dSource","rows":[[0,7.407407407407407],[1,22.22222222222222],[2,18.51851851851852],[3,33.33333333333333],[4,11.11111111111111],[5,7.407407407407407]],"fields":[{"id":"fVariable","categories":["Never","Practically never","Once in a while","Fairly often","Very often","Almost always"],"label":"Frequency of posting to social media sites","format":{"numericPattern":"###"}},{"min":7.407407407407407,"id":"fVariable1","max":33.33333333333333,"label":"Y Axis"}]}],"titles":[{"backgroundStyle":{"fill":{"g":0,"b":0,"r":0,"a":0.0},"outline":{"g":0,"b":0,"r":0,"a":0.0}},"content":["Frequency of posting to social media sites"],"style":{"font":{"weight":"bold","family":"'Helvetica Neue;Helvetica;Arial;SansSerif', sans-serif","size":"12pt"},"fill":{"g":100,"b":157,"r":0},"padding":3.0},"type":"title"}],"copyright":"(C) Copyright IBM Corp. 2011","version":"6.0","size":{"height":500.0,"width":625.0},"grammar":[{"elements":[{"position":[{"field":{"$ref":"fVariable1"}},{"field":{"$ref":"fVariable"}}],"style":{"fill":{"g":178,"b":239,"r":0},"outline":{"g":100,"b":157,"r":0},"size":"75.0%"},"data":{"$ref":"dSource"},"type":"interval"}],"coordinates":{"style":{"fill":{"g":255,"b":255,"r":255},"outline":{"g":100,"b":157,"r":0}},"dimensions":[{"axis":[{"title":["Percent"],"lineStyle":{"stroke":{"width":0.6666667},"fill":{"g":100,"b":157,"r":0}},"tickStyle":{"font":{"weight":"normal","family":"sans-serif","size":"8pt"},"fill":{"g":100,"b":157,"r":0}},"titleStyle":{"font":{"weight":"bold","family":"'Helvetica Neue;Helvetica;Arial;SansSerif', sans-serif","size":"11pt"},"fill":{"g":100,"b":157,"r":0},"padding":6.0},"markStyle":{"stroke":{"width":1.3333334},"fill":{"g":100,"b":157,"r":0}}}],"scale":{"spans":[{"tickDelta":10.0,"outRange":{"min":0.0,"max":1.0}}],"padding":{"right":"5%"}}},{"axis":[{"title":["Frequency of posting to social media sites"],"lineStyle":{"stroke":{"width":0.6666667},"fill":{"g":100,"b":157,"r":0}},"tickStyle":{"font":{"weight":"normal","family":"sans-serif","size":"8pt"},"fill":{"g":100,"b":157,"r":0}},"titleStyle":{"font":{"weight":"bold","family":"'Helvetica Neue;Helvetica;Arial;SansSerif', sans-serif","size":"11pt"},"fill":{"g":100,"b":157,"r":0},"padding":6.0},"markStyle":{"stroke":{"width":1.3333334},"fill":{"g":100,"b":157,"r":0}}}],"scale":{"spans":[{"tickDelta":1.0}],"padding":{"left":"5%","right":"5%"}}}]}}]}

Bar Chart  
Bar Chart - Frequency social media is integrated into academic activities - June 16, 2016

{"style":{"fill":{"g":255,"b":255,"r":255},"outline":{"g":0,"b":0,"r":0,"a":0.0}},"data":[{"id":"dSource","rows":[[0,7.407407407407407],[1,14.81481481481481],[2,29.62962962962963],[3,22.22222222222222],[4,18.51851851851852],[5,7.407407407407407]],"fields":[{"id":"fVariable","categories":["Never","Practically never","Once in a while","Fairly often","Very often","Almost always"],"label":"Frequency social media is integrated into academic activities","format":{"numericPattern":"###"}},{"min":7.407407407407407,"id":"fVariable1","max":29.62962962962963,"label":"Y Axis"}]}],"titles":[{"backgroundStyle":{"fill":{"g":0,"b":0,"r":0,"a":0.0},"outline":{"g":0,"b":0,"r":0,"a":0.0}},"content":["Frequency social media is integrated into academic activities"],"style":{"font":{"weight":"bold","family":"'Helvetica Neue;Helvetica;Arial;SansSerif', sans-serif","size":"12pt"},"fill":{"g":100,"b":157,"r":0},"padding":3.0},"type":"title"}],"copyright":"(C) Copyright IBM Corp. 2011","version":"6.0","size":{"height":500.0,"width":625.0},"grammar":[{"elements":[{"position":[{"field":{"$ref":"fVariable1"}},{"field":{"$ref":"fVariable"}}],"style":{"fill":{"g":178,"b":239,"r":0},"outline":{"g":100,"b":157,"r":0},"size":"75.0%"},"data":{"$ref":"dSource"},"type":"interval"}],"coordinates":{"style":{"fill":{"g":255,"b":255,"r":255},"outline":{"g":100,"b":157,"r":0}},"dimensions":[{"axis":[{"title":["Percent"],"lineStyle":{"stroke":{"width":0.6666667},"fill":{"g":100,"b":157,"r":0}},"tickStyle":{"font":{"weight":"normal","family":"sans-serif","size":"8pt"},"fill":{"g":100,"b":157,"r":0}},"titleStyle":{"font":{"weight":"bold","family":"'Helvetica Neue;Helvetica;Arial;SansSerif', sans-serif","size":"11pt"},"fill":{"g":100,"b":157,"r":0},"padding":6.0},"markStyle":{"stroke":{"width":1.3333334},"fill":{"g":100,"b":157,"r":0}}}],"scale":{"spans":[{"tickDelta":10.0,"outRange":{"min":0.0,"max":1.0}}],"padding":{"right":"5%"}}},{"axis":[{"title":["Frequency social media is integrated into academic activities"],"lineStyle":{"stroke":{"width":0.6666667},"fill":{"g":100,"b":157,"r":0}},"tickStyle":{"font":{"weight":"normal","family":"sans-serif","size":"8pt"},"fill":{"g":100,"b":157,"r":0}},"titleStyle":{"font":{"weight":"bold","family":"'Helvetica Neue;Helvetica;Arial;SansSerif', sans-serif","size":"11pt"},"fill":{"g":100,"b":157,"r":0},"padding":6.0},"markStyle":{"stroke":{"width":1.3333334},"fill":{"g":100,"b":157,"r":0}}}],"scale":{"spans":[{"tickDelta":1.0}],"padding":{"left":"5%","right":"5%"}}}]}}]}

Bar Chart  
Bar Chart - How many hours per week do you spend on social media sites - June 16, 2016

{"style":{"fill":{"g":255,"b":255,"r":255},"outline":{"g":0,"b":0,"r":0,"a":0.0}},"data":[{"id":"dSource","rows":[[0,14.81481481481481],[1,7.407407407407407],[2,18.51851851851852],[3,37.03703703703704],[4,22.22222222222222]],"fields":[{"id":"fVariable","categories":["None","Less than 1 hour","1 to 3 hours","5 t0 10 hours","More than 10 hours"],"label":"How many hours per week do you spend on social media sites","format":{"numericPattern":"###"}},{"min":7.407407407407407,"id":"fVariable1","max":37.03703703703704,"label":"Y Axis"}]}],"titles":[{"backgroundStyle":{"fill":{"g":0,"b":0,"r":0,"a":0.0},"outline":{"g":0,"b":0,"r":0,"a":0.0}},"content":["How many hours per week do you spend on social media sites"],"style":{"font":{"weight":"bold","family":"'Helvetica Neue;Helvetica;Arial;SansSerif', sans-serif","size":"12pt"},"fill":{"g":100,"b":157,"r":0},"padding":3.0},"type":"title"}],"copyright":"(C) Copyright IBM Corp. 2011","version":"6.0","size":{"height":500.0,"width":625.0},"grammar":[{"elements":[{"position":[{"field":{"$ref":"fVariable1"}},{"field":{"$ref":"fVariable"}}],"style":{"fill":{"g":178,"b":239,"r":0},"outline":{"g":100,"b":157,"r":0},"size":"75.0%"},"data":{"$ref":"dSource"},"type":"interval"}],"coordinates":{"style":{"fill":{"g":255,"b":255,"r":255},"outline":{"g":100,"b":157,"r":0}},"dimensions":[{"axis":[{"title":["Percent"],"lineStyle":{"stroke":{"width":0.6666667},"fill":{"g":100,"b":157,"r":0}},"tickStyle":{"font":{"weight":"normal","family":"sans-serif","size":"8pt"},"fill":{"g":100,"b":157,"r":0}},"titleStyle":{"font":{"weight":"bold","family":"'Helvetica Neue;Helvetica;Arial;SansSerif', sans-serif","size":"11pt"},"fill":{"g":100,"b":157,"r":0},"padding":6.0},"markStyle":{"stroke":{"width":1.3333334},"fill":{"g":100,"b":157,"r":0}}}],"scale":{"spans":[{"tickDelta":10.0,"outRange":{"min":0.0,"max":1.0}}],"padding":{"right":"5%"}}},{"axis":[{"title":["How many hours per week do you spend on social media sites"],"lineStyle":{"stroke":{"width":0.6666667},"fill":{"g":100,"b":157,"r":0}},"tickStyle":{"font":{"weight":"normal","family":"sans-serif","size":"8pt"},"fill":{"g":100,"b":157,"r":0}},"titleStyle":{"font":{"weight":"bold","family":"'Helvetica Neue;Helvetica;Arial;SansSerif', sans-serif","size":"11pt"},"fill":{"g":100,"b":157,"r":0},"padding":6.0},"markStyle":{"stroke":{"width":1.3333334},"fill":{"g":100,"b":157,"r":0}}}],"scale":{"spans":[{"tickDelta":1.0}],"padding":{"left":"5%","right":"5%"}}}]}}]}

Bar Chart  
Bar Chart - Published original online projects - June 16, 2016

{"style":{"fill":{"g":255,"b":255,"r":255},"outline":{"g":0,"b":0,"r":0,"a":0.0}},"data":[{"id":"dSource","rows":[[0,51.85185185185185],[1,18.51851851851852],[2,29.62962962962963]],"fields":[{"id":"fVariable","categories":["yes","No","Uncertain"],"label":"Published original online projects","format":{"numericPattern":"###"}},{"min":18.51851851851852,"id":"fVariable1","max":51.85185185185185,"label":"Y Axis"}]}],"titles":[{"backgroundStyle":{"fill":{"g":0,"b":0,"r":0,"a":0.0},"outline":{"g":0,"b":0,"r":0,"a":0.0}},"content":["Published original online projects"],"style":{"font":{"weight":"bold","family":"'Helvetica Neue;Helvetica;Arial;SansSerif', sans-serif","size":"12pt"},"fill":{"g":100,"b":157,"r":0},"padding":3.0},"type":"title"}],"copyright":"(C) Copyright IBM Corp. 2011","version":"6.0","size":{"height":500.0,"width":625.0},"grammar":[{"elements":[{"position":[{"field":{"$ref":"fVariable1"}},{"field":{"$ref":"fVariable"}}],"style":{"fill":{"g":178,"b":239,"r":0},"outline":{"g":100,"b":157,"r":0},"size":"75.0%"},"data":{"$ref":"dSource"},"type":"interval"}],"coordinates":{"style":{"fill":{"g":255,"b":255,"r":255},"outline":{"g":100,"b":157,"r":0}},"dimensions":[{"axis":[{"title":["Percent"],"lineStyle":{"stroke":{"width":0.6666667},"fill":{"g":100,"b":157,"r":0}},"tickStyle":{"font":{"weight":"normal","family":"sans-serif","size":"8pt"},"fill":{"g":100,"b":157,"r":0}},"titleStyle":{"font":{"weight":"bold","family":"'Helvetica Neue;Helvetica;Arial;SansSerif', sans-serif","size":"11pt"},"fill":{"g":100,"b":157,"r":0},"padding":6.0},"markStyle":{"stroke":{"width":1.3333334},"fill":{"g":100,"b":157,"r":0}}}],"scale":{"spans":[{"tickDelta":10.0,"outRange":{"min":0.0,"max":1.0}}],"padding":{"right":"5%"}}},{"axis":[{"title":["Published original online projects"],"lineStyle":{"stroke":{"width":0.6666667},"fill":{"g":100,"b":157,"r":0}},"tickStyle":{"font":{"weight":"normal","family":"sans-serif","size":"8pt"},"fill":{"g":100,"b":157,"r":0}},"titleStyle":{"font":{"weight":"bold","family":"'Helvetica Neue;Helvetica;Arial;SansSerif', sans-serif","size":"11pt"},"fill":{"g":100,"b":157,"r":0},"padding":6.0},"markStyle":{"stroke":{"width":1.3333334},"fill":{"g":100,"b":157,"r":0}}}],"scale":{"spans":[{"tickDelta":1.0}],"padding":{"left":"5%","right":"5%"}}}]}}]}

Bar Chart  
Bar Chart - Level of perceived social media expertise - June 16, 2016

{"style":{"fill":{"g":255,"b":255,"r":255},"outline":{"g":0,"b":0,"r":0,"a":0.0}},"data":[{"id":"dSource","rows":[[0,3.703703703703703],[1,22.22222222222222],[2,11.11111111111111],[3,29.62962962962963],[4,29.62962962962963],[5,3.703703703703703]],"fields":[{"id":"fVariable","categories":["Unfamiliar with no experience","Newcomer","Beginner","Average","Advanced","Expert"],"label":"Level of perceived social media expertise","format":{"numericPattern":"###"}},{"min":3.703703703703703,"id":"fVariable1","max":29.62962962962963,"label":"Y Axis"}]}],"titles":[{"backgroundStyle":{"fill":{"g":0,"b":0,"r":0,"a":0.0},"outline":{"g":0,"b":0,"r":0,"a":0.0}},"content":["Level of perceived social media expertise"],"style":{"font":{"weight":"bold","family":"'Helvetica Neue;Helvetica;Arial;SansSerif', sans-serif","size":"12pt"},"fill":{"g":100,"b":157,"r":0},"padding":3.0},"type":"title"}],"copyright":"(C) Copyright IBM Corp. 2011","version":"6.0","size":{"height":500.0,"width":625.0},"grammar":[{"elements":[{"position":[{"field":{"$ref":"fVariable1"}},{"field":{"$ref":"fVariable"}}],"style":{"fill":{"g":178,"b":239,"r":0},"outline":{"g":100,"b":157,"r":0},"size":"75.0%"},"data":{"$ref":"dSource"},"type":"interval"}],"coordinates":{"style":{"fill":{"g":255,"b":255,"r":255},"outline":{"g":100,"b":157,"r":0}},"dimensions":[{"axis":[{"title":["Percent"],"lineStyle":{"stroke":{"width":0.6666667},"fill":{"g":100,"b":157,"r":0}},"tickStyle":{"font":{"weight":"normal","family":"sans-serif","size":"8pt"},"fill":{"g":100,"b":157,"r":0}},"titleStyle":{"font":{"weight":"bold","family":"'Helvetica Neue;Helvetica;Arial;SansSerif', sans-serif","size":"11pt"},"fill":{"g":100,"b":157,"r":0},"padding":6.0},"markStyle":{"stroke":{"width":1.3333334},"fill":{"g":100,"b":157,"r":0}}}],"scale":{"spans":[{"tickDelta":10.0,"outRange":{"min":0.0,"max":1.0}}],"padding":{"right":"5%"}}},{"axis":[{"title":["Level of perceived social media expertise"],"lineStyle":{"stroke":{"width":0.6666667},"fill":{"g":100,"b":157,"r":0}},"tickStyle":{"font":{"weight":"normal","family":"sans-serif","size":"8pt"},"fill":{"g":100,"b":157,"r":0}},"titleStyle":{"font":{"weight":"bold","family":"'Helvetica Neue;Helvetica;Arial;SansSerif', sans-serif","size":"11pt"},"fill":{"g":100,"b":157,"r":0},"padding":6.0},"markStyle":{"stroke":{"width":1.3333334},"fill":{"g":100,"b":157,"r":0}}}],"scale":{"spans":[{"tickDelta":1.0}],"padding":{"left":"5%","right":"5%"}}}]}}]}

Bar Chart  
Bar Chart - Stage in the process of social media practice - June 16, 2016

{"style":{"fill":{"g":255,"b":255,"r":255},"outline":{"g":0,"b":0,"r":0,"a":0.0}},"data":[{"id":"dSource","rows":[[0,11.11111111111111],[1,18.51851851851852],[2,7.407407407407407],[3,33.33333333333333],[4,18.51851851851852],[5,11.11111111111111]],"fields":[{"id":"fVariable","categories":["Awareness","Learning","Understanding","Familiarity","Adaptation","Creative Application"],"label":"Stage in the process of social media practice","format":{"numericPattern":"###"}},{"min":7.407407407407407,"id":"fVariable1","max":33.33333333333333,"label":"Y Axis"}]}],"titles":[{"backgroundStyle":{"fill":{"g":0,"b":0,"r":0,"a":0.0},"outline":{"g":0,"b":0,"r":0,"a":0.0}},"content":["Stage in the process of social media practice"],"style":{"font":{"weight":"bold","family":"'Helvetica Neue;Helvetica;Arial;SansSerif', sans-serif","size":"12pt"},"fill":{"g":100,"b":157,"r":0},"padding":3.0},"type":"title"}],"copyright":"(C) Copyright IBM Corp. 2011","version":"6.0","size":{"height":500.0,"width":625.0},"grammar":[{"elements":[{"position":[{"field":{"$ref":"fVariable1"}},{"field":{"$ref":"fVariable"}}],"style":{"fill":{"g":178,"b":239,"r":0},"outline":{"g":100,"b":157,"r":0},"size":"75.0%"},"data":{"$ref":"dSource"},"type":"interval"}],"coordinates":{"style":{"fill":{"g":255,"b":255,"r":255},"outline":{"g":100,"b":157,"r":0}},"dimensions":[{"axis":[{"title":["Percent"],"lineStyle":{"stroke":{"width":0.6666667},"fill":{"g":100,"b":157,"r":0}},"tickStyle":{"font":{"weight":"normal","family":"sans-serif","size":"8pt"},"fill":{"g":100,"b":157,"r":0}},"titleStyle":{"font":{"weight":"bold","family":"'Helvetica Neue;Helvetica;Arial;SansSerif', sans-serif","size":"11pt"},"fill":{"g":100,"b":157,"r":0},"padding":6.0},"markStyle":{"stroke":{"width":1.3333334},"fill":{"g":100,"b":157,"r":0}}}],"scale":{"spans":[{"tickDelta":10.0,"outRange":{"min":0.0,"max":1.0}}],"padding":{"right":"5%"}}},{"axis":[{"title":["Stage in the process of social media practice"],"lineStyle":{"stroke":{"width":0.6666667},"fill":{"g":100,"b":157,"r":0}},"tickStyle":{"font":{"weight":"normal","family":"sans-serif","size":"8pt"},"fill":{"g":100,"b":157,"r":0}},"titleStyle":{"font":{"weight":"bold","family":"'Helvetica Neue;Helvetica;Arial;SansSerif', sans-serif","size":"11pt"},"fill":{"g":100,"b":157,"r":0},"padding":6.0},"markStyle":{"stroke":{"width":1.3333334},"fill":{"g":100,"b":157,"r":0}}}],"scale":{"spans":[{"tickDelta":1.0}],"padding":{"left":"5%","right":"5%"}}}]}}]}

Bar Chart  
Bar Chart - Understand website design and publishing - June 16, 2016

{"style":{"fill":{"g":255,"b":255,"r":255},"outline":{"g":0,"b":0,"r":0,"a":0.0}},"data":[{"id":"dSource","rows":[[0,44.44444444444444],[1,37.03703703703704],[2,18.51851851851852]],"fields":[{"id":"fVariable","categories":["Yes","No","Uncertain"],"label":"Understand website design and publishing","format":{"numericPattern":"###"}},{"min":18.51851851851852,"id":"fVariable1","max":44.44444444444444,"label":"Y Axis"}]}],"titles":[{"backgroundStyle":{"fill":{"g":0,"b":0,"r":0,"a":0.0},"outline":{"g":0,"b":0,"r":0,"a":0.0}},"content":["Understand website design and publishing"],"style":{"font":{"weight":"bold","family":"'Helvetica Neue;Helvetica;Arial;SansSerif', sans-serif","size":"12pt"},"fill":{"g":100,"b":157,"r":0},"padding":3.0},"type":"title"}],"copyright":"(C) Copyright IBM Corp. 2011","version":"6.0","size":{"height":500.0,"width":625.0},"grammar":[{"elements":[{"position":[{"field":{"$ref":"fVariable1"}},{"field":{"$ref":"fVariable"}}],"style":{"fill":{"g":178,"b":239,"r":0},"outline":{"g":100,"b":157,"r":0},"size":"75.0%"},"data":{"$ref":"dSource"},"type":"interval"}],"coordinates":{"style":{"fill":{"g":255,"b":255,"r":255},"outline":{"g":100,"b":157,"r":0}},"dimensions":[{"axis":[{"title":["Percent"],"lineStyle":{"stroke":{"width":0.6666667},"fill":{"g":100,"b":157,"r":0}},"tickStyle":{"font":{"weight":"normal","family":"sans-serif","size":"8pt"},"fill":{"g":100,"b":157,"r":0}},"titleStyle":{"font":{"weight":"bold","family":"'Helvetica Neue;Helvetica;Arial;SansSerif', sans-serif","size":"11pt"},"fill":{"g":100,"b":157,"r":0},"padding":6.0},"markStyle":{"stroke":{"width":1.3333334},"fill":{"g":100,"b":157,"r":0}}}],"scale":{"spans":[{"tickDelta":10.0,"outRange":{"min":0.0,"max":1.0}}],"padding":{"right":"5%"}}},{"axis":[{"title":["Understand website design and publishing"],"lineStyle":{"stroke":{"width":0.6666667},"fill":{"g":100,"b":157,"r":0}},"tickStyle":{"font":{"weight":"normal","family":"sans-serif","size":"8pt"},"fill":{"g":100,"b":157,"r":0}},"titleStyle":{"font":{"weight":"bold","family":"'Helvetica Neue;Helvetica;Arial;SansSerif', sans-serif","size":"11pt"},"fill":{"g":100,"b":157,"r":0},"padding":6.0},"markStyle":{"stroke":{"width":1.3333334},"fill":{"g":100,"b":157,"r":0}}}],"scale":{"spans":[{"tickDelta":1.0}],"padding":{"left":"5%","right":"5%"}}}]}}]}

Bar Chart  
Bar Chart - Level of interest in using social media - June 16, 2016

{"style":{"fill":{"g":255,"b":255,"r":255},"outline":{"g":0,"b":0,"r":0,"a":0.0}},"data":[{"id":"dSource","rows":[[0,7.692307692307693],[1,7.692307692307693],[2,26.92307692307692],[3,19.23076923076923],[4,38.46153846153847]],"fields":[{"id":"fVariable","categories":["Strongly disagree","Disagree","No opinion","Agree","Strongly agree"],"label":"Level of interest in using social media","format":{"numericPattern":"###"}},{"min":7.692307692307693,"id":"fVariable1","max":38.46153846153847,"label":"Y Axis"}]}],"titles":[{"backgroundStyle":{"fill":{"g":0,"b":0,"r":0,"a":0.0},"outline":{"g":0,"b":0,"r":0,"a":0.0}},"content":["Level of interest in using social media"],"style":{"font":{"weight":"bold","family":"'Helvetica Neue;Helvetica;Arial;SansSerif', sans-serif","size":"12pt"},"fill":{"g":100,"b":157,"r":0},"padding":3.0},"type":"title"}],"copyright":"(C) Copyright IBM Corp. 2011","version":"6.0","size":{"height":500.0,"width":625.0},"grammar":[{"elements":[{"position":[{"field":{"$ref":"fVariable1"}},{"field":{"$ref":"fVariable"}}],"style":{"fill":{"g":178,"b":239,"r":0},"outline":{"g":100,"b":157,"r":0},"size":"75.0%"},"data":{"$ref":"dSource"},"type":"interval"}],"coordinates":{"style":{"fill":{"g":255,"b":255,"r":255},"outline":{"g":100,"b":157,"r":0}},"dimensions":[{"axis":[{"title":["Percent"],"lineStyle":{"stroke":{"width":0.6666667},"fill":{"g":100,"b":157,"r":0}},"tickStyle":{"font":{"weight":"normal","family":"sans-serif","size":"8pt"},"fill":{"g":100,"b":157,"r":0}},"titleStyle":{"font":{"weight":"bold","family":"'Helvetica Neue;Helvetica;Arial;SansSerif', sans-serif","size":"11pt"},"fill":{"g":100,"b":157,"r":0},"padding":6.0},"markStyle":{"stroke":{"width":1.3333334},"fill":{"g":100,"b":157,"r":0}}}],"scale":{"spans":[{"tickDelta":10.0,"outRange":{"min":0.0,"max":1.0}}],"padding":{"right":"5%"}}},{"axis":[{"title":["Level of interest in using social media"],"lineStyle":{"stroke":{"width":0.6666667},"fill":{"g":100,"b":157,"r":0}},"tickStyle":{"font":{"weight":"normal","family":"sans-serif","size":"8pt"},"fill":{"g":100,"b":157,"r":0}},"titleStyle":{"font":{"weight":"bold","family":"'Helvetica Neue;Helvetica;Arial;SansSerif', sans-serif","size":"11pt"},"fill":{"g":100,"b":157,"r":0},"padding":6.0},"markStyle":{"stroke":{"width":1.3333334},"fill":{"g":100,"b":157,"r":0}}}],"scale":{"spans":[{"tickDelta":1.0}],"padding":{"left":"5%","right":"5%"}}}]}}]}

Bar Chart  
Bar Chart - Level of importance of social media - June 16, 2016

{"style":{"fill":{"g":255,"b":255,"r":255},"outline":{"g":0,"b":0,"r":0,"a":0.0}},"data":[{"id":"dSource","rows":[[0,3.846153846153846],[1,23.07692307692308],[2,19.23076923076923],[3,53.84615384615385]],"fields":[{"id":"fVariable","categories":["Disagree","No opinion","Agree","Strongly agree"],"label":"Level of importance of social media","format":{"numericPattern":"###"}},{"min":3.846153846153846,"id":"fVariable1","max":53.84615384615385,"label":"Y Axis"}]}],"titles":[{"backgroundStyle":{"fill":{"g":0,"b":0,"r":0,"a":0.0},"outline":{"g":0,"b":0,"r":0,"a":0.0}},"content":["Level of importance of social media"],"style":{"font":{"weight":"bold","family":"'Helvetica Neue;Helvetica;Arial;SansSerif', sans-serif","size":"12pt"},"fill":{"g":100,"b":157,"r":0},"padding":3.0},"type":"title"}],"copyright":"(C) Copyright IBM Corp. 2011","version":"6.0","size":{"height":500.0,"width":625.0},"grammar":[{"elements":[{"position":[{"field":{"$ref":"fVariable1"}},{"field":{"$ref":"fVariable"}}],"style":{"fill":{"g":178,"b":239,"r":0},"outline":{"g":100,"b":157,"r":0},"size":"75.0%"},"data":{"$ref":"dSource"},"type":"interval"}],"coordinates":{"style":{"fill":{"g":255,"b":255,"r":255},"outline":{"g":100,"b":157,"r":0}},"dimensions":[{"axis":[{"title":["Percent"],"lineStyle":{"stroke":{"width":0.6666667},"fill":{"g":100,"b":157,"r":0}},"tickStyle":{"font":{"weight":"normal","family":"sans-serif","size":"8pt"},"fill":{"g":100,"b":157,"r":0}},"titleStyle":{"font":{"weight":"bold","family":"'Helvetica Neue;Helvetica;Arial;SansSerif', sans-serif","size":"11pt"},"fill":{"g":100,"b":157,"r":0},"padding":6.0},"markStyle":{"stroke":{"width":1.3333334},"fill":{"g":100,"b":157,"r":0}}}],"scale":{"spans":[{"tickDelta":10.0,"outRange":{"min":0.0,"max":1.0}}],"padding":{"right":"5%"}}},{"axis":[{"title":["Level of importance of social media"],"lineStyle":{"stroke":{"width":0.6666667},"fill":{"g":100,"b":157,"r":0}},"tickStyle":{"font":{"weight":"normal","family":"sans-serif","size":"8pt"},"fill":{"g":100,"b":157,"r":0}},"titleStyle":{"font":{"weight":"bold","family":"'Helvetica Neue;Helvetica;Arial;SansSerif', sans-serif","size":"11pt"},"fill":{"g":100,"b":157,"r":0},"padding":6.0},"markStyle":{"stroke":{"width":1.3333334},"fill":{"g":100,"b":157,"r":0}}}],"scale":{"spans":[{"tickDelta":1.0}],"padding":{"left":"5%","right":"5%"}}}]}}]}

Bar Chart  
Bar Chart - Belief that social media is harmful to your reputation - June 16, 2016

{"style":{"fill":{"g":255,"b":255,"r":255},"outline":{"g":0,"b":0,"r":0,"a":0.0}},"data":[{"id":"dSource","rows":[[0,7.407407407407407],[1,48.14814814814815],[2,37.03703703703704],[3,7.407407407407407]],"fields":[{"id":"fVariable","categories":["Strongly disagree","Disagree","No opinion","Agree"],"label":"Belief that social media is harmful to your reputation","format":{"numericPattern":"###"}},{"min":7.407407407407407,"id":"fVariable1","max":48.14814814814815,"label":"Y Axis"}]}],"titles":[{"backgroundStyle":{"fill":{"g":0,"b":0,"r":0,"a":0.0},"outline":{"g":0,"b":0,"r":0,"a":0.0}},"content":["Belief that social media is harmful to your reputation"],"style":{"font":{"weight":"bold","family":"'Helvetica Neue;Helvetica;Arial;SansSerif', sans-serif","size":"12pt"},"fill":{"g":100,"b":157,"r":0},"padding":3.0},"type":"title"}],"copyright":"(C) Copyright IBM Corp. 2011","version":"6.0","size":{"height":500.0,"width":625.0},"grammar":[{"elements":[{"position":[{"field":{"$ref":"fVariable1"}},{"field":{"$ref":"fVariable"}}],"style":{"fill":{"g":178,"b":239,"r":0},"outline":{"g":100,"b":157,"r":0},"size":"75.0%"},"data":{"$ref":"dSource"},"type":"interval"}],"coordinates":{"style":{"fill":{"g":255,"b":255,"r":255},"outline":{"g":100,"b":157,"r":0}},"dimensions":[{"axis":[{"title":["Percent"],"lineStyle":{"stroke":{"width":0.6666667},"fill":{"g":100,"b":157,"r":0}},"tickStyle":{"font":{"weight":"normal","family":"sans-serif","size":"8pt"},"fill":{"g":100,"b":157,"r":0}},"titleStyle":{"font":{"weight":"bold","family":"'Helvetica Neue;Helvetica;Arial;SansSerif', sans-serif","size":"11pt"},"fill":{"g":100,"b":157,"r":0},"padding":6.0},"markStyle":{"stroke":{"width":1.3333334},"fill":{"g":100,"b":157,"r":0}}}],"scale":{"spans":[{"tickDelta":10.0,"outRange":{"min":0.0,"max":1.0}}],"padding":{"right":"5%"}}},{"axis":[{"title":["Belief that social media is harmful to your reputation"],"lineStyle":{"stroke":{"width":0.6666667},"fill":{"g":100,"b":157,"r":0}},"tickStyle":{"font":{"weight":"normal","family":"sans-serif","size":"8pt"},"fill":{"g":100,"b":157,"r":0}},"titleStyle":{"font":{"weight":"bold","family":"'Helvetica Neue;Helvetica;Arial;SansSerif', sans-serif","size":"11pt"},"fill":{"g":100,"b":157,"r":0},"padding":6.0},"markStyle":{"stroke":{"width":1.3333334},"fill":{"g":100,"b":157,"r":0}}}],"scale":{"spans":[{"tickDelta":1.0}],"padding":{"left":"5%","right":"5%"}}}]}}]}

Bar Chart  
Bar Chart - Belief that social media is used to waste time - June 16, 2016

{"style":{"fill":{"g":255,"b":255,"r":255},"outline":{"g":0,"b":0,"r":0,"a":0.0}},"data":[{"id":"dSource","rows":[[0,7.407407407407407],[1,48.14814814814815],[2,11.11111111111111],[3,25.92592592592592],[4,7.407407407407407]],"fields":[{"id":"fVariable","categories":["Strongly disagree","Disagree","No opinion","Agree","Strongly agree"],"label":"Belief that social media is used to waste time","format":{"numericPattern":"###"}},{"min":7.407407407407407,"id":"fVariable1","max":48.14814814814815,"label":"Y Axis"}]}],"titles":[{"backgroundStyle":{"fill":{"g":0,"b":0,"r":0,"a":0.0},"outline":{"g":0,"b":0,"r":0,"a":0.0}},"content":["Belief that social media is used to waste time"],"style":{"font":{"weight":"bold","family":"'Helvetica Neue;Helvetica;Arial;SansSerif', sans-serif","size":"12pt"},"fill":{"g":100,"b":157,"r":0},"padding":3.0},"type":"title"}],"copyright":"(C) Copyright IBM Corp. 2011","version":"6.0","size":{"height":500.0,"width":625.0},"grammar":[{"elements":[{"position":[{"field":{"$ref":"fVariable1"}},{"field":{"$ref":"fVariable"}}],"style":{"fill":{"g":178,"b":239,"r":0},"outline":{"g":100,"b":157,"r":0},"size":"75.0%"},"data":{"$ref":"dSource"},"type":"interval"}],"coordinates":{"style":{"fill":{"g":255,"b":255,"r":255},"outline":{"g":100,"b":157,"r":0}},"dimensions":[{"axis":[{"title":["Percent"],"lineStyle":{"stroke":{"width":0.6666667},"fill":{"g":100,"b":157,"r":0}},"tickStyle":{"font":{"weight":"normal","family":"sans-serif","size":"8pt"},"fill":{"g":100,"b":157,"r":0}},"titleStyle":{"font":{"weight":"bold","family":"'Helvetica Neue;Helvetica;Arial;SansSerif', sans-serif","size":"11pt"},"fill":{"g":100,"b":157,"r":0},"padding":6.0},"markStyle":{"stroke":{"width":1.3333334},"fill":{"g":100,"b":157,"r":0}}}],"scale":{"spans":[{"tickDelta":10.0,"outRange":{"min":0.0,"max":1.0}}],"padding":{"right":"5%"}}},{"axis":[{"title":["Belief that social media is used to waste time"],"lineStyle":{"stroke":{"width":0.6666667},"fill":{"g":100,"b":157,"r":0}},"tickStyle":{"font":{"weight":"normal","family":"sans-serif","size":"8pt"},"fill":{"g":100,"b":157,"r":0}},"titleStyle":{"font":{"weight":"bold","family":"'Helvetica Neue;Helvetica;Arial;SansSerif', sans-serif","size":"11pt"},"fill":{"g":100,"b":157,"r":0},"padding":6.0},"markStyle":{"stroke":{"width":1.3333334},"fill":{"g":100,"b":157,"r":0}}}],"scale":{"spans":[{"tickDelta":1.0}],"padding":{"left":"5%","right":"5%"}}}]}}]}

Bar Chart  
Bar Chart - Belief that you have the ability to manage social media effectively - June 16, 2016

{"style":{"fill":{"g":255,"b":255,"r":255},"outline":{"g":0,"b":0,"r":0,"a":0.0}},"data":[{"id":"dSource","rows":[[0,14.81481481481481],[1,7.407407407407407],[2,22.22222222222222],[3,37.03703703703704],[4,18.51851851851852]],"fields":[{"id":"fVariable","categories":["Strongly disagree","Disagree","No opinion","Agree","Strongly agree"],"label":"Belief that you have the ability to manage social media effectively","format":{"numericPattern":"###"}},{"min":7.407407407407407,"id":"fVariable1","max":37.03703703703704,"label":"Y Axis"}]}],"titles":[{"backgroundStyle":{"fill":{"g":0,"b":0,"r":0,"a":0.0},"outline":{"g":0,"b":0,"r":0,"a":0.0}},"content":["Belief that you have the ability to manage social media effectively"],"style":{"font":{"weight":"bold","family":"'Helvetica Neue;Helvetica;Arial;SansSerif', sans-serif","size":"12pt"},"fill":{"g":100,"b":157,"r":0},"padding":3.0},"type":"title"}],"copyright":"(C) Copyright IBM Corp. 2011","version":"6.0","size":{"height":500.0,"width":625.0},"grammar":[{"elements":[{"position":[{"field":{"$ref":"fVariable1"}},{"field":{"$ref":"fVariable"}}],"style":{"fill":{"g":178,"b":239,"r":0},"outline":{"g":100,"b":157,"r":0},"size":"75.0%"},"data":{"$ref":"dSource"},"type":"interval"}],"coordinates":{"style":{"fill":{"g":255,"b":255,"r":255},"outline":{"g":100,"b":157,"r":0}},"dimensions":[{"axis":[{"title":["Percent"],"lineStyle":{"stroke":{"width":0.6666667},"fill":{"g":100,"b":157,"r":0}},"tickStyle":{"font":{"weight":"normal","family":"sans-serif","size":"8pt"},"fill":{"g":100,"b":157,"r":0}},"titleStyle":{"font":{"weight":"bold","family":"'Helvetica Neue;Helvetica;Arial;SansSerif', sans-serif","size":"11pt"},"fill":{"g":100,"b":157,"r":0},"padding":6.0},"markStyle":{"stroke":{"width":1.3333334},"fill":{"g":100,"b":157,"r":0}}}],"scale":{"spans":[{"tickDelta":10.0,"outRange":{"min":0.0,"max":1.0}}],"padding":{"right":"5%"}}},{"axis":[{"title":["Belief that you have the ability to manage social media effectively"],"lineStyle":{"stroke":{"width":0.6666667},"fill":{"g":100,"b":157,"r":0}},"tickStyle":{"font":{"weight":"normal","family":"sans-serif","size":"8pt"},"fill":{"g":100,"b":157,"r":0}},"titleStyle":{"font":{"weight":"bold","family":"'Helvetica Neue;Helvetica;Arial;SansSerif', sans-serif","size":"11pt"},"fill":{"g":100,"b":157,"r":0},"padding":6.0},"markStyle":{"stroke":{"width":1.3333334},"fill":{"g":100,"b":157,"r":0}}}],"scale":{"spans":[{"tickDelta":1.0}],"padding":{"left":"5%","right":"5%"}}}]}}]}

Bar Chart  
Bar Chart - Belief that social media promotes social reputation - June 16, 2016

{"style":{"fill":{"g":255,"b":255,"r":255},"outline":{"g":0,"b":0,"r":0,"a":0.0}},"data":[{"id":"dSource","rows":[[0,7.407407407407407],[1,25.92592592592592],[2,37.03703703703704],[3,29.62962962962963]],"fields":[{"id":"fVariable","categories":["Disagree","No opinion","Agree","Strongly agree"],"label":"Belief that social media promotes social reputation","format":{"numericPattern":"###"}},{"min":7.407407407407407,"id":"fVariable1","max":37.03703703703704,"label":"Y Axis"}]}],"titles":[{"backgroundStyle":{"fill":{"g":0,"b":0,"r":0,"a":0.0},"outline":{"g":0,"b":0,"r":0,"a":0.0}},"content":["Belief that social media promotes social reputation"],"style":{"font":{"weight":"bold","family":"'Helvetica Neue;Helvetica;Arial;SansSerif', sans-serif","size":"12pt"},"fill":{"g":100,"b":157,"r":0},"padding":3.0},"type":"title"}],"copyright":"(C) Copyright IBM Corp. 2011","version":"6.0","size":{"height":500.0,"width":625.0},"grammar":[{"elements":[{"position":[{"field":{"$ref":"fVariable1"}},{"field":{"$ref":"fVariable"}}],"style":{"fill":{"g":178,"b":239,"r":0},"outline":{"g":100,"b":157,"r":0},"size":"75.0%"},"data":{"$ref":"dSource"},"type":"interval"}],"coordinates":{"style":{"fill":{"g":255,"b":255,"r":255},"outline":{"g":100,"b":157,"r":0}},"dimensions":[{"axis":[{"title":["Percent"],"lineStyle":{"stroke":{"width":0.6666667},"fill":{"g":100,"b":157,"r":0}},"tickStyle":{"font":{"weight":"normal","family":"sans-serif","size":"8pt"},"fill":{"g":100,"b":157,"r":0}},"titleStyle":{"font":{"weight":"bold","family":"'Helvetica Neue;Helvetica;Arial;SansSerif', sans-serif","size":"11pt"},"fill":{"g":100,"b":157,"r":0},"padding":6.0},"markStyle":{"stroke":{"width":1.3333334},"fill":{"g":100,"b":157,"r":0}}}],"scale":{"spans":[{"tickDelta":10.0,"outRange":{"min":0.0,"max":1.0}}],"padding":{"right":"5%"}}},{"axis":[{"title":["Belief that social media promotes social reputation"],"lineStyle":{"stroke":{"width":0.6666667},"fill":{"g":100,"b":157,"r":0}},"tickStyle":{"font":{"weight":"normal","family":"sans-serif","size":"8pt"},"fill":{"g":100,"b":157,"r":0}},"titleStyle":{"font":{"weight":"bold","family":"'Helvetica Neue;Helvetica;Arial;SansSerif', sans-serif","size":"11pt"},"fill":{"g":100,"b":157,"r":0},"padding":6.0},"markStyle":{"stroke":{"width":1.3333334},"fill":{"g":100,"b":157,"r":0}}}],"scale":{"spans":[{"tickDelta":1.0}],"padding":{"left":"5%","right":"5%"}}}]}}]}

Bar Chart  
Bar Chart - Belief that social media is intended for personal use - June 16, 2016

{"style":{"fill":{"g":255,"b":255,"r":255},"outline":{"g":0,"b":0,"r":0,"a":0.0}},"data":[{"id":"dSource","rows":[[0,3.703703703703703],[1,37.03703703703704],[2,29.62962962962963],[3,22.22222222222222],[4,7.407407407407407]],"fields":[{"id":"fVariable","categories":["Strongly disagree","Disagree","No opinoon","Agree","Strongly agree"],"label":"Belief that social media is intended for personal use","format":{"numericPattern":"###"}},{"min":3.703703703703703,"id":"fVariable1","max":37.03703703703704,"label":"Y Axis"}]}],"titles":[{"backgroundStyle":{"fill":{"g":0,"b":0,"r":0,"a":0.0},"outline":{"g":0,"b":0,"r":0,"a":0.0}},"content":["Belief that social media is intended for personal use"],"style":{"font":{"weight":"bold","family":"'Helvetica Neue;Helvetica;Arial;SansSerif', sans-serif","size":"12pt"},"fill":{"g":100,"b":157,"r":0},"padding":3.0},"type":"title"}],"copyright":"(C) Copyright IBM Corp. 2011","version":"6.0","size":{"height":500.0,"width":625.0},"grammar":[{"elements":[{"position":[{"field":{"$ref":"fVariable1"}},{"field":{"$ref":"fVariable"}}],"style":{"fill":{"g":178,"b":239,"r":0},"outline":{"g":100,"b":157,"r":0},"size":"75.0%"},"data":{"$ref":"dSource"},"type":"interval"}],"coordinates":{"style":{"fill":{"g":255,"b":255,"r":255},"outline":{"g":100,"b":157,"r":0}},"dimensions":[{"axis":[{"title":["Percent"],"lineStyle":{"stroke":{"width":0.6666667},"fill":{"g":100,"b":157,"r":0}},"tickStyle":{"font":{"weight":"normal","family":"sans-serif","size":"8pt"},"fill":{"g":100,"b":157,"r":0}},"titleStyle":{"font":{"weight":"bold","family":"'Helvetica Neue;Helvetica;Arial;SansSerif', sans-serif","size":"11pt"},"fill":{"g":100,"b":157,"r":0},"padding":6.0},"markStyle":{"stroke":{"width":1.3333334},"fill":{"g":100,"b":157,"r":0}}}],"scale":{"spans":[{"tickDelta":10.0,"outRange":{"min":0.0,"max":1.0}}],"padding":{"right":"5%"}}},{"axis":[{"title":["Belief that social media is intended for personal use"],"lineStyle":{"stroke":{"width":0.6666667},"fill":{"g":100,"b":157,"r":0}},"tickStyle":{"font":{"weight":"normal","family":"sans-serif","size":"8pt"},"fill":{"g":100,"b":157,"r":0}},"titleStyle":{"font":{"weight":"bold","family":"'Helvetica Neue;Helvetica;Arial;SansSerif', sans-serif","size":"11pt"},"fill":{"g":100,"b":157,"r":0},"padding":6.0},"markStyle":{"stroke":{"width":1.3333334},"fill":{"g":100,"b":157,"r":0}}}],"scale":{"spans":[{"tickDelta":1.0}],"padding":{"left":"5%","right":"5%"}}}]}}]}

Bar Chart  
Bar Chart - Belief that social media develops communication skills - June 16, 2016

{"style":{"fill":{"g":255,"b":255,"r":255},"outline":{"g":0,"b":0,"r":0,"a":0.0}},"data":[{"id":"dSource","rows":[[0,7.407407407407407],[1,11.11111111111111],[2,14.81481481481481],[3,48.14814814814815],[4,18.51851851851852]],"fields":[{"id":"fVariable","categories":["Strongly disagree","Disagree","No opinion","Agree","Strongly agree"],"label":"Belief that social media develops communication skills","format":{"numericPattern":"###"}},{"min":7.407407407407407,"id":"fVariable1","max":48.14814814814815,"label":"Y Axis"}]}],"titles":[{"backgroundStyle":{"fill":{"g":0,"b":0,"r":0,"a":0.0},"outline":{"g":0,"b":0,"r":0,"a":0.0}},"content":["Belief that social media develops communication skills"],"style":{"font":{"weight":"bold","family":"'Helvetica Neue;Helvetica;Arial;SansSerif', sans-serif","size":"12pt"},"fill":{"g":100,"b":157,"r":0},"padding":3.0},"type":"title"}],"copyright":"(C) Copyright IBM Corp. 2011","version":"6.0","size":{"height":500.0,"width":625.0},"grammar":[{"elements":[{"position":[{"field":{"$ref":"fVariable1"}},{"field":{"$ref":"fVariable"}}],"style":{"fill":{"g":178,"b":239,"r":0},"outline":{"g":100,"b":157,"r":0},"size":"75.0%"},"data":{"$ref":"dSource"},"type":"interval"}],"coordinates":{"style":{"fill":{"g":255,"b":255,"r":255},"outline":{"g":100,"b":157,"r":0}},"dimensions":[{"axis":[{"title":["Percent"],"lineStyle":{"stroke":{"width":0.6666667},"fill":{"g":100,"b":157,"r":0}},"tickStyle":{"font":{"weight":"normal","family":"sans-serif","size":"8pt"},"fill":{"g":100,"b":157,"r":0}},"titleStyle":{"font":{"weight":"bold","family":"'Helvetica Neue;Helvetica;Arial;SansSerif', sans-serif","size":"11pt"},"fill":{"g":100,"b":157,"r":0},"padding":6.0},"markStyle":{"stroke":{"width":1.3333334},"fill":{"g":100,"b":157,"r":0}}}],"scale":{"spans":[{"tickDelta":10.0,"outRange":{"min":0.0,"max":1.0}}],"padding":{"right":"5%"}}},{"axis":[{"title":["Belief that social media develops communication skills"],"lineStyle":{"stroke":{"width":0.6666667},"fill":{"g":100,"b":157,"r":0}},"tickStyle":{"font":{"weight":"normal","family":"sans-serif","size":"8pt"},"fill":{"g":100,"b":157,"r":0}},"titleStyle":{"font":{"weight":"bold","family":"'Helvetica Neue;Helvetica;Arial;SansSerif', sans-serif","size":"11pt"},"fill":{"g":100,"b":157,"r":0},"padding":6.0},"markStyle":{"stroke":{"width":1.3333334},"fill":{"g":100,"b":157,"r":0}}}],"scale":{"spans":[{"tickDelta":1.0}],"padding":{"left":"5%","right":"5%"}}}]}}]}

Bar Chart  
Bar Chart - Belief that social media is a valuable communication skill - June 16, 2016

{"style":{"fill":{"g":255,"b":255,"r":255},"outline":{"g":0,"b":0,"r":0,"a":0.0}},"data":[{"id":"dSource","rows":[[0,25.92592592592592],[1,51.85185185185185],[2,22.22222222222222]],"fields":[{"id":"fVariable","categories":["No opinion","Agree","Strongly agree"],"label":"Belief that social media is a valuable communication skill","format":{"numericPattern":"###"}},{"min":22.22222222222222,"id":"fVariable1","max":51.85185185185185,"label":"Y Axis"}]}],"titles":[{"backgroundStyle":{"fill":{"g":0,"b":0,"r":0,"a":0.0},"outline":{"g":0,"b":0,"r":0,"a":0.0}},"content":["Belief that social media is a valuable communication skill"],"style":{"font":{"weight":"bold","family":"'Helvetica Neue;Helvetica;Arial;SansSerif', sans-serif","size":"12pt"},"fill":{"g":100,"b":157,"r":0},"padding":3.0},"type":"title"}],"copyright":"(C) Copyright IBM Corp. 2011","version":"6.0","size":{"height":500.0,"width":625.0},"grammar":[{"elements":[{"position":[{"field":{"$ref":"fVariable1"}},{"field":{"$ref":"fVariable"}}],"style":{"fill":{"g":178,"b":239,"r":0},"outline":{"g":100,"b":157,"r":0},"size":"75.0%"},"data":{"$ref":"dSource"},"type":"interval"}],"coordinates":{"style":{"fill":{"g":255,"b":255,"r":255},"outline":{"g":100,"b":157,"r":0}},"dimensions":[{"axis":[{"title":["Percent"],"lineStyle":{"stroke":{"width":0.6666667},"fill":{"g":100,"b":157,"r":0}},"tickStyle":{"font":{"weight":"normal","family":"sans-serif","size":"8pt"},"fill":{"g":100,"b":157,"r":0}},"titleStyle":{"font":{"weight":"bold","family":"'Helvetica Neue;Helvetica;Arial;SansSerif', sans-serif","size":"11pt"},"fill":{"g":100,"b":157,"r":0},"padding":6.0},"markStyle":{"stroke":{"width":1.3333334},"fill":{"g":100,"b":157,"r":0}}}],"scale":{"spans":[{"tickDelta":10.0,"outRange":{"min":0.0,"max":1.0}}],"padding":{"right":"5%"}}},{"axis":[{"title":["Belief that social media is a valuable communication skill"],"lineStyle":{"stroke":{"width":0.6666667},"fill":{"g":100,"b":157,"r":0}},"tickStyle":{"font":{"weight":"normal","family":"sans-serif","size":"8pt"},"fill":{"g":100,"b":157,"r":0}},"titleStyle":{"font":{"weight":"bold","family":"'Helvetica Neue;Helvetica;Arial;SansSerif', sans-serif","size":"11pt"},"fill":{"g":100,"b":157,"r":0},"padding":6.0},"markStyle":{"stroke":{"width":1.3333334},"fill":{"g":100,"b":157,"r":0}}}],"scale":{"spans":[{"tickDelta":1.0}],"padding":{"left":"5%","right":"5%"}}}]}}]}

Bar Chart  
Bar Chart - Belief that the costs of social media outweigh the benefits to professional reputation -(more) June 16, 2016(less)

{"style":{"fill":{"g":255,"b":255,"r":255},"outline":{"g":0,"b":0,"r":0,"a":0.0}},"data":[{"id":"dSource","rows":[[0,11.11111111111111],[1,37.03703703703704],[2,25.92592592592592],[3,18.51851851851852],[4,7.407407407407407]],"fields":[{"id":"fVariable","categories":["Strongly disagree","Disagree","No opinion","Agree","Strongly agree"],"label":"Belief that the costs of social media outweigh the benefits to professional reputation","format":{"numericPattern":"###"}},{"min":7.407407407407407,"id":"fVariable1","max":37.03703703703704,"label":"Y Axis"}]}],"titles":[{"backgroundStyle":{"fill":{"g":0,"b":0,"r":0,"a":0.0},"outline":{"g":0,"b":0,"r":0,"a":0.0}},"content":["Belief that the costs of social media outweigh the benefits to professional reputation"],"style":{"font":{"weight":"bold","family":"'Helvetica Neue;Helvetica;Arial;SansSerif', sans-serif","size":"12pt"},"fill":{"g":100,"b":157,"r":0},"padding":3.0},"type":"title"}],"copyright":"(C) Copyright IBM Corp. 2011","version":"6.0","size":{"height":500.0,"width":625.0},"grammar":[{"elements":[{"position":[{"field":{"$ref":"fVariable1"}},{"field":{"$ref":"fVariable"}}],"style":{"fill":{"g":178,"b":239,"r":0},"outline":{"g":100,"b":157,"r":0},"size":"75.0%"},"data":{"$ref":"dSource"},"type":"interval"}],"coordinates":{"style":{"fill":{"g":255,"b":255,"r":255},"outline":{"g":100,"b":157,"r":0}},"dimensions":[{"axis":[{"title":["Percent"],"lineStyle":{"stroke":{"width":0.6666667},"fill":{"g":100,"b":157,"r":0}},"tickStyle":{"font":{"weight":"normal","family":"sans-serif","size":"8pt"},"fill":{"g":100,"b":157,"r":0}},"titleStyle":{"font":{"weight":"bold","family":"'Helvetica Neue;Helvetica;Arial;SansSerif', sans-serif","size":"11pt"},"fill":{"g":100,"b":157,"r":0},"padding":6.0},"markStyle":{"stroke":{"width":1.3333334},"fill":{"g":100,"b":157,"r":0}}}],"scale":{"spans":[{"tickDelta":10.0,"outRange":{"min":0.0,"max":1.0}}],"padding":{"right":"5%"}}},{"axis":[{"title":["Belief that the costs of social media outweigh the benefits to professional reputation"],"lineStyle":{"stroke":{"width":0.6666667},"fill":{"g":100,"b":157,"r":0}},"tickStyle":{"font":{"weight":"normal","family":"sans-serif","size":"8pt"},"fill":{"g":100,"b":157,"r":0}},"titleStyle":{"font":{"weight":"bold","family":"'Helvetica Neue;Helvetica;Arial;SansSerif', sans-serif","size":"11pt"},"fill":{"g":100,"b":157,"r":0},"padding":6.0},"markStyle":{"stroke":{"width":1.3333334},"fill":{"g":100,"b":157,"r":0}}}],"scale":{"spans":[{"tickDelta":1.0}],"padding":{"left":"5%","right":"5%"}}}]}}]}

Bar Chart  
Bar Chart - Belief that social media is an effective tool for all students - June 16, 2016

{"style":{"fill":{"g":255,"b":255,"r":255},"outline":{"g":0,"b":0,"r":0,"a":0.0}},"data":[{"id":"dSource","rows":[[0,3.703703703703703],[1,14.81481481481481],[2,29.62962962962963],[3,33.33333333333333],[4,18.51851851851852]],"fields":[{"id":"fVariable","categories":["Strongly disagree","Disagree","No opinion","Agree","Strongly agree"],"label":"Belief that social media is an effective tool for all students","format":{"numericPattern":"###"}},{"min":3.703703703703703,"id":"fVariable1","max":33.33333333333333,"label":"Y Axis"}]}],"titles":[{"backgroundStyle":{"fill":{"g":0,"b":0,"r":0,"a":0.0},"outline":{"g":0,"b":0,"r":0,"a":0.0}},"content":["Belief that social media is an effective tool for all students"],"style":{"font":{"weight":"bold","family":"'Helvetica Neue;Helvetica;Arial;SansSerif', sans-serif","size":"12pt"},"fill":{"g":100,"b":157,"r":0},"padding":3.0},"type":"title"}],"copyright":"(C) Copyright IBM Corp. 2011","version":"6.0","size":{"height":500.0,"width":625.0},"grammar":[{"elements":[{"position":[{"field":{"$ref":"fVariable1"}},{"field":{"$ref":"fVariable"}}],"style":{"fill":{"g":178,"b":239,"r":0},"outline":{"g":100,"b":157,"r":0},"size":"75.0%"},"data":{"$ref":"dSource"},"type":"interval"}],"coordinates":{"style":{"fill":{"g":255,"b":255,"r":255},"outline":{"g":100,"b":157,"r":0}},"dimensions":[{"axis":[{"title":["Percent"],"lineStyle":{"stroke":{"width":0.6666667},"fill":{"g":100,"b":157,"r":0}},"tickStyle":{"font":{"weight":"normal","family":"sans-serif","size":"8pt"},"fill":{"g":100,"b":157,"r":0}},"titleStyle":{"font":{"weight":"bold","family":"'Helvetica Neue;Helvetica;Arial;SansSerif', sans-serif","size":"11pt"},"fill":{"g":100,"b":157,"r":0},"padding":6.0},"markStyle":{"stroke":{"width":1.3333334},"fill":{"g":100,"b":157,"r":0}}}],"scale":{"spans":[{"tickDelta":10.0,"outRange":{"min":0.0,"max":1.0}}],"padding":{"right":"5%"}}},{"axis":[{"title":["Belief that social media is an effective tool for all students"],"lineStyle":{"stroke":{"width":0.6666667},"fill":{"g":100,"b":157,"r":0}},"tickStyle":{"font":{"weight":"normal","family":"sans-serif","size":"8pt"},"fill":{"g":100,"b":157,"r":0}},"titleStyle":{"font":{"weight":"bold","family":"'Helvetica Neue;Helvetica;Arial;SansSerif', sans-serif","size":"11pt"},"fill":{"g":100,"b":157,"r":0},"padding":6.0},"markStyle":{"stroke":{"width":1.3333334},"fill":{"g":100,"b":157,"r":0}}}],"scale":{"spans":[{"tickDelta":1.0}],"padding":{"left":"5%","right":"5%"}}}]}}]}

Bar Chart  
Bar Chart - Belief that social media enhances professional development - June 16, 2016

{"style":{"fill":{"g":255,"b":255,"r":255},"outline":{"g":0,"b":0,"r":0,"a":0.0}},"data":[{"id":"dSource","rows":[[0,3.703703703703703],[1,7.407407407407407],[2,37.03703703703704],[3,29.62962962962963],[4,22.22222222222222]],"fields":[{"id":"fVariable","categories":["Strongly disagree","Disagree","No opinion","Agree","Strongly agree"],"label":"Belief that social media enhances professional development","format":{"numericPattern":"###"}},{"min":3.703703703703703,"id":"fVariable1","max":37.03703703703704,"label":"Y Axis"}]}],"titles":[{"backgroundStyle":{"fill":{"g":0,"b":0,"r":0,"a":0.0},"outline":{"g":0,"b":0,"r":0,"a":0.0}},"content":["Belief that social media enhances professional development"],"style":{"font":{"weight":"bold","family":"'Helvetica Neue;Helvetica;Arial;SansSerif', sans-serif","size":"12pt"},"fill":{"g":100,"b":157,"r":0},"padding":3.0},"type":"title"}],"copyright":"(C) Copyright IBM Corp. 2011","version":"6.0","size":{"height":500.0,"width":625.0},"grammar":[{"elements":[{"position":[{"field":{"$ref":"fVariable1"}},{"field":{"$ref":"fVariable"}}],"style":{"fill":{"g":178,"b":239,"r":0},"outline":{"g":100,"b":157,"r":0},"size":"75.0%"},"data":{"$ref":"dSource"},"type":"interval"}],"coordinates":{"style":{"fill":{"g":255,"b":255,"r":255},"outline":{"g":100,"b":157,"r":0}},"dimensions":[{"axis":[{"title":["Percent"],"lineStyle":{"stroke":{"width":0.6666667},"fill":{"g":100,"b":157,"r":0}},"tickStyle":{"font":{"weight":"normal","family":"sans-serif","size":"8pt"},"fill":{"g":100,"b":157,"r":0}},"titleStyle":{"font":{"weight":"bold","family":"'Helvetica Neue;Helvetica;Arial;SansSerif', sans-serif","size":"11pt"},"fill":{"g":100,"b":157,"r":0},"padding":6.0},"markStyle":{"stroke":{"width":1.3333334},"fill":{"g":100,"b":157,"r":0}}}],"scale":{"spans":[{"tickDelta":10.0,"outRange":{"min":0.0,"max":1.0}}],"padding":{"right":"5%"}}},{"axis":[{"title":["Belief that social media enhances professional development"],"lineStyle":{"stroke":{"width":0.6666667},"fill":{"g":100,"b":157,"r":0}},"tickStyle":{"font":{"weight":"normal","family":"sans-serif","size":"8pt"},"fill":{"g":100,"b":157,"r":0}},"titleStyle":{"font":{"weight":"bold","family":"'Helvetica Neue;Helvetica;Arial;SansSerif', sans-serif","size":"11pt"},"fill":{"g":100,"b":157,"r":0},"padding":6.0},"markStyle":{"stroke":{"width":1.3333334},"fill":{"g":100,"b":157,"r":0}}}],"scale":{"spans":[{"tickDelta":1.0}],"padding":{"left":"5%","right":"5%"}}}]}}]}

Bar Chart  
Bar Chart - Belief that social media promotes the development of interpersonal skills - June 16, 201(more)6(less)

{"style":{"fill":{"g":255,"b":255,"r":255},"outline":{"g":0,"b":0,"r":0,"a":0.0}},"data":[{"id":"dSource","rows":[[0,7.407407407407407],[1,7.407407407407407],[2,48.14814814814815],[3,25.92592592592592],[4,11.11111111111111]],"fields":[{"id":"fVariable","categories":["Strongly disagree","Disagree","No opinion","Agree","Strongly agree"],"label":"Belief that social media promotes the development of interpersonal skills","format":{"numericPattern":"###"}},{"min":7.407407407407407,"id":"fVariable1","max":48.14814814814815,"label":"Y Axis"}]}],"titles":[{"backgroundStyle":{"fill":{"g":0,"b":0,"r":0,"a":0.0},"outline":{"g":0,"b":0,"r":0,"a":0.0}},"content":["Belief that social media promotes the development of interpersonal skills"],"style":{"font":{"weight":"bold","family":"'Helvetica Neue;Helvetica;Arial;SansSerif', sans-serif","size":"12pt"},"fill":{"g":100,"b":157,"r":0},"padding":3.0},"type":"title"}],"copyright":"(C) Copyright IBM Corp. 2011","version":"6.0","size":{"height":500.0,"width":625.0},"grammar":[{"elements":[{"position":[{"field":{"$ref":"fVariable1"}},{"field":{"$ref":"fVariable"}}],"style":{"fill":{"g":178,"b":239,"r":0},"outline":{"g":100,"b":157,"r":0},"size":"75.0%"},"data":{"$ref":"dSource"},"type":"interval"}],"coordinates":{"style":{"fill":{"g":255,"b":255,"r":255},"outline":{"g":100,"b":157,"r":0}},"dimensions":[{"axis":[{"title":["Percent"],"lineStyle":{"stroke":{"width":0.6666667},"fill":{"g":100,"b":157,"r":0}},"tickStyle":{"font":{"weight":"normal","family":"sans-serif","size":"8pt"},"fill":{"g":100,"b":157,"r":0}},"titleStyle":{"font":{"weight":"bold","family":"'Helvetica Neue;Helvetica;Arial;SansSerif', sans-serif","size":"11pt"},"fill":{"g":100,"b":157,"r":0},"padding":6.0},"markStyle":{"stroke":{"width":1.3333334},"fill":{"g":100,"b":157,"r":0}}}],"scale":{"spans":[{"tickDelta":10.0,"outRange":{"min":0.0,"max":1.0}}],"padding":{"right":"5%"}}},{"axis":[{"title":["Belief that social media promotes the development of interpersonal skills"],"lineStyle":{"stroke":{"width":0.6666667},"fill":{"g":100,"b":157,"r":0}},"tickStyle":{"font":{"weight":"normal","family":"sans-serif","size":"8pt"},"fill":{"g":100,"b":157,"r":0}},"titleStyle":{"font":{"weight":"bold","family":"'Helvetica Neue;Helvetica;Arial;SansSerif', sans-serif","size":"11pt"},"fill":{"g":100,"b":157,"r":0},"padding":6.0},"markStyle":{"stroke":{"width":1.3333334},"fill":{"g":100,"b":157,"r":0}}}],"scale":{"spans":[{"tickDelta":1.0}],"padding":{"left":"5%","right":"5%"}}}]}}]}

Bar Chart  
Bar Chart - Belief that social media increase stress and anxiety - June 16, 2016

{"style":{"fill":{"g":255,"b":255,"r":255},"outline":{"g":0,"b":0,"r":0,"a":0.0}},"data":[{"id":"dSource","rows":[[0,22.22222222222222],[1,48.14814814814815],[2,22.22222222222222],[3,7.407407407407407]],"fields":[{"id":"fVariable","categories":["Disagree","No opinion","Agree","Strongly agree"],"label":"Belief that social media increase stress and anxiety","format":{"numericPattern":"###"}},{"min":7.407407407407407,"id":"fVariable1","max":48.14814814814815,"label":"Y Axis"}]}],"titles":[{"backgroundStyle":{"fill":{"g":0,"b":0,"r":0,"a":0.0},"outline":{"g":0,"b":0,"r":0,"a":0.0}},"content":["Belief that social media increase stress and anxiety"],"style":{"font":{"weight":"bold","family":"'Helvetica Neue;Helvetica;Arial;SansSerif', sans-serif","size":"12pt"},"fill":{"g":100,"b":157,"r":0},"padding":3.0},"type":"title"}],"copyright":"(C) Copyright IBM Corp. 2011","version":"6.0","size":{"height":500.0,"width":625.0},"grammar":[{"elements":[{"position":[{"field":{"$ref":"fVariable1"}},{"field":{"$ref":"fVariable"}}],"style":{"fill":{"g":178,"b":239,"r":0},"outline":{"g":100,"b":157,"r":0},"size":"75.0%"},"data":{"$ref":"dSource"},"type":"interval"}],"coordinates":{"style":{"fill":{"g":255,"b":255,"r":255},"outline":{"g":100,"b":157,"r":0}},"dimensions":[{"axis":[{"title":["Percent"],"lineStyle":{"stroke":{"width":0.6666667},"fill":{"g":100,"b":157,"r":0}},"tickStyle":{"font":{"weight":"normal","family":"sans-serif","size":"8pt"},"fill":{"g":100,"b":157,"r":0}},"titleStyle":{"font":{"weight":"bold","family":"'Helvetica Neue;Helvetica;Arial;SansSerif', sans-serif","size":"11pt"},"fill":{"g":100,"b":157,"r":0},"padding":6.0},"markStyle":{"stroke":{"width":1.3333334},"fill":{"g":100,"b":157,"r":0}}}],"scale":{"spans":[{"tickDelta":10.0,"outRange":{"min":0.0,"max":1.0}}],"padding":{"right":"5%"}}},{"axis":[{"title":["Belief that social media increase stress and anxiety"],"lineStyle":{"stroke":{"width":0.6666667},"fill":{"g":100,"b":157,"r":0}},"tickStyle":{"font":{"weight":"normal","family":"sans-serif","size":"8pt"},"fill":{"g":100,"b":157,"r":0}},"titleStyle":{"font":{"weight":"bold","family":"'Helvetica Neue;Helvetica;Arial;SansSerif', sans-serif","size":"11pt"},"fill":{"g":100,"b":157,"r":0},"padding":6.0},"markStyle":{"stroke":{"width":1.3333334},"fill":{"g":100,"b":157,"r":0}}}],"scale":{"spans":[{"tickDelta":1.0}],"padding":{"left":"5%","right":"5%"}}}]}}]}

Log  
Log - Log - June 16, 2016

CORRELATIONS  
  /VARIABLES=AggregFamSites AggregJoinedSites AggregPostSites  
    Onaveragehowmanyhoursperweekdoyouspendusingsocialme  
    FrequentlysocialmediaisintegratedinAcademicactivities Howfrequentlydoyoupostorshareinformation  
    Selectthelevelthatbestdescribesyou Howfrequentlydoyouvisitsocialmediasites  
    Choosethestagethatbestdescribeswhereyouareintheproces  
    Onascaleof1to5with5beingthehighestlevelofinterest Onascaleof1to5with5beingthehighestlevelofimportan  
    Socialmediaisharmfultoyourprofessionalreputation Socialmediaismostlyusedtowastetime  
    SocialmediaiseffectivebecauseIbelieveIcanimplementits Socialmediapromotessocialreputation  
    Socialmediaismostlyintendedforpersonaluse Socialmediapromotesthedevelopmentofcommunicationskills  
    Socialmediaisavaluableprofessionaltool Socialmediaistoocostlyintermsofrisktoprofessionalrepu  
    Socialmediaisaneffectivetoolforstudentsofallabilities Socialmediaenhancesmyprofessionaldevelopment  
    Socialmediapromotesthedevelopmentofinterpersonalskills Socialmediaincreasesstressandanxiety  
  /PRINT=TWOTAIL NOSIG  
  /MISSING=PAIRWISE.

Correlations

CorrelationsCorrelations, table, 1 levels of column headers and 2 levels of row headers, table with 25 columns and 73 rows

|  |  |  |  |  |  |  |  |  |  |  |  |  |  |  |  |  |  |  |  |  |  |  |  |  |  |
| --- | --- | --- | --- | --- | --- | --- | --- | --- | --- | --- | --- | --- | --- | --- | --- | --- | --- | --- | --- | --- | --- | --- | --- | --- | --- |
|  | | Aggregate of number of social media sites familiar | Aggregate of number of social media sites joined | Aggregate of number of social media sites posted | How many hours per week do you spend on social media sites | Frequency social media is integrated into academic activities | Frequency of posting to social media sites | Level of perceived social media expertise | Frequency of visits to social media sites | Stage in the process of social media practice | Level of interest in using social media | Level of importance of social media | Belief that social media is harmful to your reputation | Belief that social media is used to waste time | Belief that you have the ability to manage social media effectively | Belief that social media promotes social reputation | Belief that social media is intended for personal use | Belief that social media develops communication skills | Belief that social media is a valuable communication skill | Belief that the costs of social media outweigh the benefits to professional reputation | Belief that social media is an effective tool for all students | Belief that social media enhances professional development | Belief that social media promotes the development of interpersonal skills | Belief that social media increase stress and anxiety |  |
| Aggregate of number of social media sites familiar | Pearson Correlation | 1 | .581\*\* | .555\*\* | .507\*\* | .247 | .380 | .674\*\* | .429\* | .616\*\* | .439\* | .284 | .008 | -.153 | .395\* | .109 | -.028 | .056 | .218 | -.050 | .432\* | .276 | .089 | -.426\* |  |
|  | Sig. (2-tailed) |  | .001 | .003 | .007 | .214 | .051 | .000 | .026 | .001 | .025 | .159 | .967 | .445 | .041 | .587 | .889 | .782 | .275 | .803 | .025 | .164 | .660 | .027 |  |
|  | N | 27 | 27 | 27 | 27 | 27 | 27 | 27 | 27 | 27 | 26 | 26 | 27 | 27 | 27 | 27 | 27 | 27 | 27 | 27 | 27 | 27 | 27 | 27 |  |
| Aggregate of number of social media sites joined | Pearson Correlation | .581\*\* | 1 | .793\*\* | .456\* | .429\* | .701\*\* | .697\*\* | .373 | .599\*\* | .551\*\* | .560\*\* | -.161 | -.572\*\* | .470\* | .270 | -.245 | .455\* | .352 | -.330 | .441\* | .538\*\* | .345 | -.397\* |  |
|  | Sig. (2-tailed) | .001 |  | .000 | .017 | .026 | .000 | .000 | .055 | .001 | .004 | .003 | .423 | .002 | .013 | .173 | .219 | .017 | .071 | .093 | .021 | .004 | .078 | .040 |  |
|  | N | 27 | 27 | 27 | 27 | 27 | 27 | 27 | 27 | 27 | 26 | 26 | 27 | 27 | 27 | 27 | 27 | 27 | 27 | 27 | 27 | 27 | 27 | 27 |  |
| Aggregate of number of social media sites posted | Pearson Correlation | .555\*\* | .793\*\* | 1 | .426\* | .480\* | .625\*\* | .651\*\* | .330 | .564\*\* | .485\* | .459\* | -.208 | -.603\*\* | .352 | .366 | -.107 | .352 | .333 | -.280 | .442\* | .479\* | .286 | -.359 |  |
|  | Sig. (2-tailed) | .003 | .000 |  | .027 | .011 | .000 | .000 | .093 | .002 | .012 | .018 | .299 | .001 | .072 | .061 | .594 | .072 | .090 | .157 | .021 | .011 | .148 | .066 |  |
|  | N | 27 | 27 | 27 | 27 | 27 | 27 | 27 | 27 | 27 | 26 | 26 | 27 | 27 | 27 | 27 | 27 | 27 | 27 | 27 | 27 | 27 | 27 | 27 |  |
| How many hours per week do you spend on social media sites | Pearson Correlation | .507\*\* | .456\* | .426\* | 1 | .541\*\* | .654\*\* | .684\*\* | .879\*\* | .602\*\* | .368 | .307 | -.319 | -.456\* | .408\* | .410\* | -.197 | .496\*\* | .221 | -.404\* | .349 | .408\* | -.003 | -.624\*\* |  |
|  | Sig. (2-tailed) | .007 | .017 | .027 |  | .004 | .000 | .000 | .000 | .001 | .064 | .127 | .105 | .017 | .035 | .034 | .324 | .009 | .267 | .037 | .074 | .035 | .988 | .001 |  |
|  | N | 27 | 27 | 27 | 27 | 27 | 27 | 27 | 27 | 27 | 26 | 26 | 27 | 27 | 27 | 27 | 27 | 27 | 27 | 27 | 27 | 27 | 27 | 27 |  |
| Frequency social media is integrated into academic activities | Pearson Correlation | .247 | .429\* | .480\* | .541\*\* | 1 | .644\*\* | .576\*\* | .600\*\* | .457\* | .463\* | .439\* | -.308 | -.460\* | .276 | .498\*\* | -.243 | .407\* | .418\* | -.308 | .343 | .341 | .202 | -.458\* |  |
|  | Sig. (2-tailed) | .214 | .026 | .011 | .004 |  | .000 | .002 | .001 | .017 | .017 | .025 | .119 | .016 | .164 | .008 | .221 | .035 | .030 | .119 | .080 | .082 | .311 | .016 |  |
|  | N | 27 | 27 | 27 | 27 | 27 | 27 | 27 | 27 | 27 | 26 | 26 | 27 | 27 | 27 | 27 | 27 | 27 | 27 | 27 | 27 | 27 | 27 | 27 |  |
| Frequency of posting to social media sites | Pearson Correlation | .380 | .701\*\* | .625\*\* | .654\*\* | .644\*\* | 1 | .749\*\* | .680\*\* | .729\*\* | .694\*\* | .679\*\* | -.483\* | -.648\*\* | .559\*\* | .519\*\* | -.413\* | .623\*\* | .455\* | -.378 | .562\*\* | .685\*\* | .307 | -.607\*\* |  |
|  | Sig. (2-tailed) | .051 | .000 | .000 | .000 | .000 |  | .000 | .000 | .000 | .000 | .000 | .011 | .000 | .002 | .006 | .032 | .001 | .017 | .052 | .002 | .000 | .119 | .001 |  |
|  | N | 27 | 27 | 27 | 27 | 27 | 27 | 27 | 27 | 27 | 26 | 26 | 27 | 27 | 27 | 27 | 27 | 27 | 27 | 27 | 27 | 27 | 27 | 27 |  |
| Level of perceived social media expertise | Pearson Correlation | .674\*\* | .697\*\* | .651\*\* | .684\*\* | .576\*\* | .749\*\* | 1 | .610\*\* | .845\*\* | .649\*\* | .606\*\* | -.404\* | -.447\* | .466\* | .314 | -.129 | .346 | .317 | -.285 | .583\*\* | .603\*\* | .172 | -.565\*\* |  |
|  | Sig. (2-tailed) | .000 | .000 | .000 | .000 | .002 | .000 |  | .001 | .000 | .000 | .001 | .037 | .019 | .014 | .110 | .522 | .077 | .107 | .150 | .001 | .001 | .390 | .002 |  |
|  | N | 27 | 27 | 27 | 27 | 27 | 27 | 27 | 27 | 27 | 26 | 26 | 27 | 27 | 27 | 27 | 27 | 27 | 27 | 27 | 27 | 27 | 27 | 27 |  |
| Frequency of visits to social media sites | Pearson Correlation | .429\* | .373 | .330 | .879\*\* | .600\*\* | .680\*\* | .610\*\* | 1 | .568\*\* | .387 | .419\* | -.448\* | -.530\*\* | .413\* | .521\*\* | -.319 | .589\*\* | .419\* | -.552\*\* | .354 | .468\* | .101 | -.632\*\* |  |
|  | Sig. (2-tailed) | .026 | .055 | .093 | .000 | .001 | .000 | .001 |  | .002 | .051 | .033 | .019 | .004 | .032 | .005 | .105 | .001 | .029 | .003 | .070 | .014 | .616 | .000 |  |
|  | N | 27 | 27 | 27 | 27 | 27 | 27 | 27 | 27 | 27 | 26 | 26 | 27 | 27 | 27 | 27 | 27 | 27 | 27 | 27 | 27 | 27 | 27 | 27 |  |
| Stage in the process of social media practice | Pearson Correlation | .616\*\* | .599\*\* | .564\*\* | .602\*\* | .457\* | .729\*\* | .845\*\* | .568\*\* | 1 | .659\*\* | .408\* | -.217 | -.500\*\* | .451\* | .476\* | -.210 | .322 | .233 | -.277 | .704\*\* | .544\*\* | .257 | -.705\*\* |  |
|  | Sig. (2-tailed) | .001 | .001 | .002 | .001 | .017 | .000 | .000 | .002 |  | .000 | .038 | .277 | .008 | .018 | .012 | .293 | .102 | .242 | .162 | .000 | .003 | .195 | .000 |  |
|  | N | 27 | 27 | 27 | 27 | 27 | 27 | 27 | 27 | 27 | 26 | 26 | 27 | 27 | 27 | 27 | 27 | 27 | 27 | 27 | 27 | 27 | 27 | 27 |  |
| Level of interest in using social media | Pearson Correlation | .439\* | .551\*\* | .485\* | .368 | .463\* | .694\*\* | .649\*\* | .387 | .659\*\* | 1 | .774\*\* | -.319 | -.376 | .533\*\* | .402\* | -.296 | .400\* | .335 | -.153 | .600\*\* | .567\*\* | .128 | -.635\*\* |  |
|  | Sig. (2-tailed) | .025 | .004 | .012 | .064 | .017 | .000 | .000 | .051 | .000 |  | .000 | .113 | .058 | .005 | .042 | .142 | .043 | .094 | .455 | .001 | .002 | .533 | .000 |  |
|  | N | 26 | 26 | 26 | 26 | 26 | 26 | 26 | 26 | 26 | 26 | 26 | 26 | 26 | 26 | 26 | 26 | 26 | 26 | 26 | 26 | 26 | 26 | 26 |  |
| Level of importance of social media | Pearson Correlation | .284 | .560\*\* | .459\* | .307 | .439\* | .679\*\* | .606\*\* | .419\* | .408\* | .774\*\* | 1 | -.540\*\* | -.379 | .465\* | .224 | -.216 | .486\* | .422\* | -.170 | .314 | .567\*\* | .173 | -.522\*\* |  |
|  | Sig. (2-tailed) | .159 | .003 | .018 | .127 | .025 | .000 | .001 | .033 | .038 | .000 |  | .004 | .056 | .017 | .272 | .289 | .012 | .032 | .407 | .119 | .003 | .398 | .006 |  |
|  | N | 26 | 26 | 26 | 26 | 26 | 26 | 26 | 26 | 26 | 26 | 26 | 26 | 26 | 26 | 26 | 26 | 26 | 26 | 26 | 26 | 26 | 26 | 26 |  |
| Belief that social media is harmful to your reputation | Pearson Correlation | .008 | -.161 | -.208 | -.319 | -.308 | -.483\* | -.404\* | -.448\* | -.217 | -.319 | -.540\*\* | 1 | .340 | -.331 | -.256 | .192 | -.494\*\* | -.548\*\* | .458\* | -.413\* | -.641\*\* | -.256 | .310 |  |
|  | Sig. (2-tailed) | .967 | .423 | .299 | .105 | .119 | .011 | .037 | .019 | .277 | .113 | .004 |  | .083 | .091 | .198 | .336 | .009 | .003 | .016 | .032 | .000 | .198 | .116 |  |
|  | N | 27 | 27 | 27 | 27 | 27 | 27 | 27 | 27 | 27 | 26 | 26 | 27 | 27 | 27 | 27 | 27 | 27 | 27 | 27 | 27 | 27 | 27 | 27 |  |
| Belief that social media is used to waste time | Pearson Correlation | -.153 | -.572\*\* | -.603\*\* | -.456\* | -.460\* | -.648\*\* | -.447\* | -.530\*\* | -.500\*\* | -.376 | -.379 | .340 | 1 | -.377 | -.666\*\* | .597\*\* | -.736\*\* | -.577\*\* | .367 | -.402\* | -.714\*\* | -.536\*\* | .497\*\* |  |
|  | Sig. (2-tailed) | .445 | .002 | .001 | .017 | .016 | .000 | .019 | .004 | .008 | .058 | .056 | .083 |  | .052 | .000 | .001 | .000 | .002 | .060 | .038 | .000 | .004 | .008 |  |
|  | N | 27 | 27 | 27 | 27 | 27 | 27 | 27 | 27 | 27 | 26 | 26 | 27 | 27 | 27 | 27 | 27 | 27 | 27 | 27 | 27 | 27 | 27 | 27 |  |
| Belief that you have the ability to manage social media effectively | Pearson Correlation | .395\* | .470\* | .352 | .408\* | .276 | .559\*\* | .466\* | .413\* | .451\* | .533\*\* | .465\* | -.331 | -.377 | 1 | .224 | -.491\*\* | .360 | .349 | -.063 | .737\*\* | .621\*\* | .358 | -.596\*\* |  |
|  | Sig. (2-tailed) | .041 | .013 | .072 | .035 | .164 | .002 | .014 | .032 | .018 | .005 | .017 | .091 | .052 |  | .260 | .009 | .065 | .074 | .756 | .000 | .001 | .067 | .001 |  |
|  | N | 27 | 27 | 27 | 27 | 27 | 27 | 27 | 27 | 27 | 26 | 26 | 27 | 27 | 27 | 27 | 27 | 27 | 27 | 27 | 27 | 27 | 27 | 27 |  |
| Belief that social media promotes social reputation | Pearson Correlation | .109 | .270 | .366 | .410\* | .498\*\* | .519\*\* | .314 | .521\*\* | .476\* | .402\* | .224 | -.256 | -.666\*\* | .224 | 1 | -.447\* | .707\*\* | .577\*\* | -.284 | .396\* | .345 | .354 | -.456\* |  |
|  | Sig. (2-tailed) | .587 | .173 | .061 | .034 | .008 | .006 | .110 | .005 | .012 | .042 | .272 | .198 | .000 | .260 |  | .020 | .000 | .002 | .152 | .041 | .078 | .070 | .017 |  |
|  | N | 27 | 27 | 27 | 27 | 27 | 27 | 27 | 27 | 27 | 26 | 26 | 27 | 27 | 27 | 27 | 27 | 27 | 27 | 27 | 27 | 27 | 27 | 27 |  |
| Belief that social media is intended for personal use | Pearson Correlation | -.028 | -.245 | -.107 | -.197 | -.243 | -.413\* | -.129 | -.319 | -.210 | -.296 | -.216 | .192 | .597\*\* | -.491\*\* | -.447\* | 1 | -.575\*\* | -.530\*\* | .147 | -.411\* | -.561\*\* | -.526\*\* | .271 |  |
|  | Sig. (2-tailed) | .889 | .219 | .594 | .324 | .221 | .032 | .522 | .105 | .293 | .142 | .289 | .336 | .001 | .009 | .020 |  | .002 | .004 | .463 | .033 | .002 | .005 | .172 |  |
|  | N | 27 | 27 | 27 | 27 | 27 | 27 | 27 | 27 | 27 | 26 | 26 | 27 | 27 | 27 | 27 | 27 | 27 | 27 | 27 | 27 | 27 | 27 | 27 |  |
| Belief that social media develops communication skills | Pearson Correlation | .056 | .455\* | .352 | .496\*\* | .407\* | .623\*\* | .346 | .589\*\* | .322 | .400\* | .486\* | -.494\*\* | -.736\*\* | .360 | .707\*\* | -.575\*\* | 1 | .690\*\* | -.380 | .285 | .654\*\* | .550\*\* | -.439\* |  |
|  | Sig. (2-tailed) | .782 | .017 | .072 | .009 | .035 | .001 | .077 | .001 | .102 | .043 | .012 | .009 | .000 | .065 | .000 | .002 |  | .000 | .051 | .149 | .000 | .003 | .022 |  |
|  | N | 27 | 27 | 27 | 27 | 27 | 27 | 27 | 27 | 27 | 26 | 26 | 27 | 27 | 27 | 27 | 27 | 27 | 27 | 27 | 27 | 27 | 27 | 27 |  |
| Belief that social media is a valuable communication skill | Pearson Correlation | .218 | .352 | .333 | .221 | .418\* | .455\* | .317 | .419\* | .233 | .335 | .422\* | -.548\*\* | -.577\*\* | .349 | .577\*\* | -.530\*\* | .690\*\* | 1 | -.350 | .325 | .655\*\* | .493\*\* | -.306 |  |
|  | Sig. (2-tailed) | .275 | .071 | .090 | .267 | .030 | .017 | .107 | .029 | .242 | .094 | .032 | .003 | .002 | .074 | .002 | .004 | .000 |  | .074 | .098 | .000 | .009 | .121 |  |
|  | N | 27 | 27 | 27 | 27 | 27 | 27 | 27 | 27 | 27 | 26 | 26 | 27 | 27 | 27 | 27 | 27 | 27 | 27 | 27 | 27 | 27 | 27 | 27 |  |
| Belief that the costs of social media outweigh the benefits to professional reputation | Pearson Correlation | -.050 | -.330 | -.280 | -.404\* | -.308 | -.378 | -.285 | -.552\*\* | -.277 | -.153 | -.170 | .458\* | .367 | -.063 | -.284 | .147 | -.380 | -.350 | 1 | -.208 | -.353 | -.006 | .198 |  |
|  | Sig. (2-tailed) | .803 | .093 | .157 | .037 | .119 | .052 | .150 | .003 | .162 | .455 | .407 | .016 | .060 | .756 | .152 | .463 | .051 | .074 |  | .299 | .071 | .976 | .321 |  |
|  | N | 27 | 27 | 27 | 27 | 27 | 27 | 27 | 27 | 27 | 26 | 26 | 27 | 27 | 27 | 27 | 27 | 27 | 27 | 27 | 27 | 27 | 27 | 27 |  |
| Belief that social media is an effective tool for all students | Pearson Correlation | .432\* | .441\* | .442\* | .349 | .343 | .562\*\* | .583\*\* | .354 | .704\*\* | .600\*\* | .314 | -.413\* | -.402\* | .737\*\* | .396\* | -.411\* | .285 | .325 | -.208 | 1 | .652\*\* | .437\* | -.570\*\* |  |
|  | Sig. (2-tailed) | .025 | .021 | .021 | .074 | .080 | .002 | .001 | .070 | .000 | .001 | .119 | .032 | .038 | .000 | .041 | .033 | .149 | .098 | .299 |  | .000 | .023 | .002 |  |
|  | N | 27 | 27 | 27 | 27 | 27 | 27 | 27 | 27 | 27 | 26 | 26 | 27 | 27 | 27 | 27 | 27 | 27 | 27 | 27 | 27 | 27 | 27 | 27 |  |
| Belief that social media enhances professional development | Pearson Correlation | .276 | .538\*\* | .479\* | .408\* | .341 | .685\*\* | .603\*\* | .468\* | .544\*\* | .567\*\* | .567\*\* | -.641\*\* | -.714\*\* | .621\*\* | .345 | -.561\*\* | .654\*\* | .655\*\* | -.353 | .652\*\* | 1 | .533\*\* | -.526\*\* |  |
|  | Sig. (2-tailed) | .164 | .004 | .011 | .035 | .082 | .000 | .001 | .014 | .003 | .002 | .003 | .000 | .000 | .001 | .078 | .002 | .000 | .000 | .071 | .000 |  | .004 | .005 |  |
|  | N | 27 | 27 | 27 | 27 | 27 | 27 | 27 | 27 | 27 | 26 | 26 | 27 | 27 | 27 | 27 | 27 | 27 | 27 | 27 | 27 | 27 | 27 | 27 |  |
| Belief that social media promotes the development of interpersonal skills | Pearson Correlation | .089 | .345 | .286 | -.003 | .202 | .307 | .172 | .101 | .257 | .128 | .173 | -.256 | -.536\*\* | .358 | .354 | -.526\*\* | .550\*\* | .493\*\* | -.006 | .437\* | .533\*\* | 1 | -.132 |  |
|  | Sig. (2-tailed) | .660 | .078 | .148 | .988 | .311 | .119 | .390 | .616 | .195 | .533 | .398 | .198 | .004 | .067 | .070 | .005 | .003 | .009 | .976 | .023 | .004 |  | .511 |  |
|  | N | 27 | 27 | 27 | 27 | 27 | 27 | 27 | 27 | 27 | 26 | 26 | 27 | 27 | 27 | 27 | 27 | 27 | 27 | 27 | 27 | 27 | 27 | 27 |  |
| Belief that social media increase stress and anxiety | Pearson Correlation | -.426\* | -.397\* | -.359 | -.624\*\* | -.458\* | -.607\*\* | -.565\*\* | -.632\*\* | -.705\*\* | -.635\*\* | -.522\*\* | .310 | .497\*\* | -.596\*\* | -.456\* | .271 | -.439\* | -.306 | .198 | -.570\*\* | -.526\*\* | -.132 | 1 |  |
|  | Sig. (2-tailed) | .027 | .040 | .066 | .001 | .016 | .001 | .002 | .000 | .000 | .000 | .006 | .116 | .008 | .001 | .017 | .172 | .022 | .121 | .321 | .002 | .005 | .511 |  |  |
|  | N | 27 | 27 | 27 | 27 | 27 | 27 | 27 | 27 | 27 | 26 | 26 | 27 | 27 | 27 | 27 | 27 | 27 | 27 | 27 | 27 | 27 | 27 | 27 |  |
|  |  |  |  |  |  |  |  |  |  |  |  |  |  |  |  |  |  |  |  |  |  |  |  |  |  |
| --- | --- | --- | --- | --- | --- | --- | --- | --- | --- | --- | --- | --- | --- | --- | --- | --- | --- | --- | --- | --- | --- | --- | --- | --- | --- |
| \*\*. Correlation is significant at the 0.01 level (2-tailed). | | | | | | | | | | | | | | | | | | | | | | | | |  |
| \*. Correlation is significant at the 0.05 level (2-tailed). | | | | | | | | | | | | | | | | | | | | | | | | |  |
|  |  |  |  |  |  |  |  |  |  |  |  |  |  |  |  |  |  |  |  |  |  |  |  |  |  |

Log

CORRELATIONS  
  /VARIABLES=Selectthelevelthatbestdescribesyou  
    Choosethestagethatbestdescribeswhereyouareintheproces  
    Onascaleof1to5with5beingthehighestlevelofinterest Onascaleof1to5with5beingthehighestlevelofimportan  
    Socialmediaisharmfultoyourprofessionalreputation Socialmediaismostlyusedtowastetime  
    SocialmediaiseffectivebecauseIbelieveIcanimplementits Socialmediapromotessocialreputation  
    Socialmediaismostlyintendedforpersonaluse Socialmediapromotesthedevelopmentofcommunicationskills  
    Socialmediaisavaluableprofessionaltool Socialmediaistoocostlyintermsofrisktoprofessionalrepu  
    Socialmediaisaneffectivetoolforstudentsofallabilities Socialmediaenhancesmyprofessionaldevelopment  
    Socialmediapromotesthedevelopmentofinterpersonalskills Socialmediaincreasesstressandanxiety  
  /PRINT=TWOTAIL NOSIG  
  /MISSING=PAIRWISE.

Correlations

CorrelationsCorrelations, table, 1 levels of column headers and 2 levels of row headers, table with 18 columns and 52 rows

|  |  |  |  |  |  |  |  |  |  |  |  |  |  |  |  |  |  |  |
| --- | --- | --- | --- | --- | --- | --- | --- | --- | --- | --- | --- | --- | --- | --- | --- | --- | --- | --- |
|  | | Level of perceived social media expertise | Stage in the process of social media practice | Level of interest in using social media | Level of importance of social media | Belief that social media is harmful to your reputation | Belief that social media is used to waste time | Belief that you have the ability to manage social media effectively | Belief that social media promotes social reputation | Belief that social media is intended for personal use | Belief that social media develops communication skills | Belief that social media is a valuable communication skill | Belief that the costs of social media outweigh the benefits to professional reputation | Belief that social media is an effective tool for all students | Belief that social media enhances professional development | Belief that social media promotes the development of interpersonal skills | Belief that social media increase stress and anxiety |  |
| Level of perceived social media expertise | Pearson Correlation | 1 | .845\*\* | .649\*\* | .606\*\* | -.404\* | -.447\* | .466\* | .314 | -.129 | .346 | .317 | -.285 | .583\*\* | .603\*\* | .172 | -.565\*\* |  |
|  | Sig. (2-tailed) |  | .000 | .000 | .001 | .037 | .019 | .014 | .110 | .522 | .077 | .107 | .150 | .001 | .001 | .390 | .002 |  |
|  | N | 27 | 27 | 26 | 26 | 27 | 27 | 27 | 27 | 27 | 27 | 27 | 27 | 27 | 27 | 27 | 27 |  |
| Stage in the process of social media practice | Pearson Correlation | .845\*\* | 1 | .659\*\* | .408\* | -.217 | -.500\*\* | .451\* | .476\* | -.210 | .322 | .233 | -.277 | .704\*\* | .544\*\* | .257 | -.705\*\* |  |
|  | Sig. (2-tailed) | .000 |  | .000 | .038 | .277 | .008 | .018 | .012 | .293 | .102 | .242 | .162 | .000 | .003 | .195 | .000 |  |
|  | N | 27 | 27 | 26 | 26 | 27 | 27 | 27 | 27 | 27 | 27 | 27 | 27 | 27 | 27 | 27 | 27 |  |
| Level of interest in using social media | Pearson Correlation | .649\*\* | .659\*\* | 1 | .774\*\* | -.319 | -.376 | .533\*\* | .402\* | -.296 | .400\* | .335 | -.153 | .600\*\* | .567\*\* | .128 | -.635\*\* |  |
|  | Sig. (2-tailed) | .000 | .000 |  | .000 | .113 | .058 | .005 | .042 | .142 | .043 | .094 | .455 | .001 | .002 | .533 | .000 |  |
|  | N | 26 | 26 | 26 | 26 | 26 | 26 | 26 | 26 | 26 | 26 | 26 | 26 | 26 | 26 | 26 | 26 |  |
| Level of importance of social media | Pearson Correlation | .606\*\* | .408\* | .774\*\* | 1 | -.540\*\* | -.379 | .465\* | .224 | -.216 | .486\* | .422\* | -.170 | .314 | .567\*\* | .173 | -.522\*\* |  |
|  | Sig. (2-tailed) | .001 | .038 | .000 |  | .004 | .056 | .017 | .272 | .289 | .012 | .032 | .407 | .119 | .003 | .398 | .006 |  |
|  | N | 26 | 26 | 26 | 26 | 26 | 26 | 26 | 26 | 26 | 26 | 26 | 26 | 26 | 26 | 26 | 26 |  |
| Belief that social media is harmful to your reputation | Pearson Correlation | -.404\* | -.217 | -.319 | -.540\*\* | 1 | .340 | -.331 | -.256 | .192 | -.494\*\* | -.548\*\* | .458\* | -.413\* | -.641\*\* | -.256 | .310 |  |
|  | Sig. (2-tailed) | .037 | .277 | .113 | .004 |  | .083 | .091 | .198 | .336 | .009 | .003 | .016 | .032 | .000 | .198 | .116 |  |
|  | N | 27 | 27 | 26 | 26 | 27 | 27 | 27 | 27 | 27 | 27 | 27 | 27 | 27 | 27 | 27 | 27 |  |
| Belief that social media is used to waste time | Pearson Correlation | -.447\* | -.500\*\* | -.376 | -.379 | .340 | 1 | -.377 | -.666\*\* | .597\*\* | -.736\*\* | -.577\*\* | .367 | -.402\* | -.714\*\* | -.536\*\* | .497\*\* |  |
|  | Sig. (2-tailed) | .019 | .008 | .058 | .056 | .083 |  | .052 | .000 | .001 | .000 | .002 | .060 | .038 | .000 | .004 | .008 |  |
|  | N | 27 | 27 | 26 | 26 | 27 | 27 | 27 | 27 | 27 | 27 | 27 | 27 | 27 | 27 | 27 | 27 |  |
| Belief that you have the ability to manage social media effectively | Pearson Correlation | .466\* | .451\* | .533\*\* | .465\* | -.331 | -.377 | 1 | .224 | -.491\*\* | .360 | .349 | -.063 | .737\*\* | .621\*\* | .358 | -.596\*\* |  |
|  | Sig. (2-tailed) | .014 | .018 | .005 | .017 | .091 | .052 |  | .260 | .009 | .065 | .074 | .756 | .000 | .001 | .067 | .001 |  |
|  | N | 27 | 27 | 26 | 26 | 27 | 27 | 27 | 27 | 27 | 27 | 27 | 27 | 27 | 27 | 27 | 27 |  |
| Belief that social media promotes social reputation | Pearson Correlation | .314 | .476\* | .402\* | .224 | -.256 | -.666\*\* | .224 | 1 | -.447\* | .707\*\* | .577\*\* | -.284 | .396\* | .345 | .354 | -.456\* |  |
|  | Sig. (2-tailed) | .110 | .012 | .042 | .272 | .198 | .000 | .260 |  | .020 | .000 | .002 | .152 | .041 | .078 | .070 | .017 |  |
|  | N | 27 | 27 | 26 | 26 | 27 | 27 | 27 | 27 | 27 | 27 | 27 | 27 | 27 | 27 | 27 | 27 |  |
| Belief that social media is intended for personal use | Pearson Correlation | -.129 | -.210 | -.296 | -.216 | .192 | .597\*\* | -.491\*\* | -.447\* | 1 | -.575\*\* | -.530\*\* | .147 | -.411\* | -.561\*\* | -.526\*\* | .271 |  |
|  | Sig. (2-tailed) | .522 | .293 | .142 | .289 | .336 | .001 | .009 | .020 |  | .002 | .004 | .463 | .033 | .002 | .005 | .172 |  |
|  | N | 27 | 27 | 26 | 26 | 27 | 27 | 27 | 27 | 27 | 27 | 27 | 27 | 27 | 27 | 27 | 27 |  |
| Belief that social media develops communication skills | Pearson Correlation | .346 | .322 | .400\* | .486\* | -.494\*\* | -.736\*\* | .360 | .707\*\* | -.575\*\* | 1 | .690\*\* | -.380 | .285 | .654\*\* | .550\*\* | -.439\* |  |
|  | Sig. (2-tailed) | .077 | .102 | .043 | .012 | .009 | .000 | .065 | .000 | .002 |  | .000 | .051 | .149 | .000 | .003 | .022 |  |
|  | N | 27 | 27 | 26 | 26 | 27 | 27 | 27 | 27 | 27 | 27 | 27 | 27 | 27 | 27 | 27 | 27 |  |
| Belief that social media is a valuable communication skill | Pearson Correlation | .317 | .233 | .335 | .422\* | -.548\*\* | -.577\*\* | .349 | .577\*\* | -.530\*\* | .690\*\* | 1 | -.350 | .325 | .655\*\* | .493\*\* | -.306 |  |
|  | Sig. (2-tailed) | .107 | .242 | .094 | .032 | .003 | .002 | .074 | .002 | .004 | .000 |  | .074 | .098 | .000 | .009 | .121 |  |
|  | N | 27 | 27 | 26 | 26 | 27 | 27 | 27 | 27 | 27 | 27 | 27 | 27 | 27 | 27 | 27 | 27 |  |
| Belief that the costs of social media outweigh the benefits to professional reputation | Pearson Correlation | -.285 | -.277 | -.153 | -.170 | .458\* | .367 | -.063 | -.284 | .147 | -.380 | -.350 | 1 | -.208 | -.353 | -.006 | .198 |  |
|  | Sig. (2-tailed) | .150 | .162 | .455 | .407 | .016 | .060 | .756 | .152 | .463 | .051 | .074 |  | .299 | .071 | .976 | .321 |  |
|  | N | 27 | 27 | 26 | 26 | 27 | 27 | 27 | 27 | 27 | 27 | 27 | 27 | 27 | 27 | 27 | 27 |  |
| Belief that social media is an effective tool for all students | Pearson Correlation | .583\*\* | .704\*\* | .600\*\* | .314 | -.413\* | -.402\* | .737\*\* | .396\* | -.411\* | .285 | .325 | -.208 | 1 | .652\*\* | .437\* | -.570\*\* |  |
|  | Sig. (2-tailed) | .001 | .000 | .001 | .119 | .032 | .038 | .000 | .041 | .033 | .149 | .098 | .299 |  | .000 | .023 | .002 |  |
|  | N | 27 | 27 | 26 | 26 | 27 | 27 | 27 | 27 | 27 | 27 | 27 | 27 | 27 | 27 | 27 | 27 |  |
| Belief that social media enhances professional development | Pearson Correlation | .603\*\* | .544\*\* | .567\*\* | .567\*\* | -.641\*\* | -.714\*\* | .621\*\* | .345 | -.561\*\* | .654\*\* | .655\*\* | -.353 | .652\*\* | 1 | .533\*\* | -.526\*\* |  |
|  | Sig. (2-tailed) | .001 | .003 | .002 | .003 | .000 | .000 | .001 | .078 | .002 | .000 | .000 | .071 | .000 |  | .004 | .005 |  |
|  | N | 27 | 27 | 26 | 26 | 27 | 27 | 27 | 27 | 27 | 27 | 27 | 27 | 27 | 27 | 27 | 27 |  |
| Belief that social media promotes the development of interpersonal skills | Pearson Correlation | .172 | .257 | .128 | .173 | -.256 | -.536\*\* | .358 | .354 | -.526\*\* | .550\*\* | .493\*\* | -.006 | .437\* | .533\*\* | 1 | -.132 |  |
|  | Sig. (2-tailed) | .390 | .195 | .533 | .398 | .198 | .004 | .067 | .070 | .005 | .003 | .009 | .976 | .023 | .004 |  | .511 |  |
|  | N | 27 | 27 | 26 | 26 | 27 | 27 | 27 | 27 | 27 | 27 | 27 | 27 | 27 | 27 | 27 | 27 |  |
| Belief that social media increase stress and anxiety | Pearson Correlation | -.565\*\* | -.705\*\* | -.635\*\* | -.522\*\* | .310 | .497\*\* | -.596\*\* | -.456\* | .271 | -.439\* | -.306 | .198 | -.570\*\* | -.526\*\* | -.132 | 1 |  |
|  | Sig. (2-tailed) | .002 | .000 | .000 | .006 | .116 | .008 | .001 | .017 | .172 | .022 | .121 | .321 | .002 | .005 | .511 |  |  |
|  | N | 27 | 27 | 26 | 26 | 27 | 27 | 27 | 27 | 27 | 27 | 27 | 27 | 27 | 27 | 27 | 27 |  |
|  |  |  |  |  |  |  |  |  |  |  |  |  |  |  |  |  |  |  |
| --- | --- | --- | --- | --- | --- | --- | --- | --- | --- | --- | --- | --- | --- | --- | --- | --- | --- | --- |
| \*\*. Correlation is significant at the 0.01 level (2-tailed). | | | | | | | | | | | | | | | | | |  |
| \*. Correlation is significant at the 0.05 level (2-tailed). | | | | | | | | | | | | | | | | | |  |
|  |  |  |  |  |  |  |  |  |  |  |  |  |  |  |  |  |  |  |

IBM SPSS Web Report

X

ABOUT

:   Created Using: IBM SPSS Statistics 23
:   Creation Date: Jun 16, 2016
:   Document Version: OriginalSaved Copy
:   Saved Date:  Jun 16, 2016

Navigation Controls

:   Contents - Opens and closes the list of charts and tables in the Web Report
:   Next & Previous - Display the next or previous table or chart in the Web Report
:   Help - Opens Help

Toolbar Buttons

|  |  |
| --- | --- |
|  | Undo - Undoes the last change in the document. |
|  | Edit - Open the Editor tool for tables and charts. Certain editing options are only available when you are connected to an Internet server. |
|  | Save - Creates a new copy of the Web Report with the saved changes. |
|  | Print - Prints the current object when in Object View and all objects in Page View. |
|  | Page View - Switches the Web Report to display all the tables and charts on a single page. |
|  | Object View - Switches the Web Report so that each table or chart is displayed one at a time. |

Connecting to a Server

:   The status of the Web Report's connection to an Internet server appears in the top right corner of the Web Report.
:   An Internet connection is not required to open a Web Report. With a saved copy of the Web Report you can view all of the charts and tables, and have some limited editing ability, when not connected to the Internet.
:   Connecting a Web Report to an Internet server will enable far greater editing capabilities for tables and for charts.

- If the author specified an Internet server when they created the Web Report, the Web Report will attempt to connect to the server automatically when it is opened.
- If the Web Report does not connect to a server, click on the server Status Message to open tools to retry the connection, try a different server, or enter a new server address.
- For information about adding the enhanced controls to your Internet Server, go to http://www.ibm.com/developerworks/spssdevcentral.
- If you specify a new server connection, the preferred format is http://xxx.xxx.xxx.xxx:xxxx.

Editing Tables

|  |  |
| --- | --- |
| Some of this functionality is only available when connected to an Internet server. | |
|  | Create a chart - Create a chart from the selected cells in the table. |
|  | Pivot and Sort - Transpose, sort, and pivot the table. |
|  | Background color - The background color of the selected cells. |
|  | Text Color and Style - Font color, style, and size. |
|  | Number Format - Font color, style, and size. |

Editing Charts

|  |  |
| --- | --- |
| All of this functionality is only available when connected to an Internet server. | |
|  | Chart Size - Change the height and width of the chart |
|  | Background color - The background color of the selected object. |
|  | Border and Line Style - The color and thickness of the line or border. |
|  | Text Color and Style - Font color, style, and size. |
|  | Number Format - Font color, style, and size. |
|  | Axis Properties - Change the scale and display axis titles and ticks. |
