# Supplementary material for: Data to inform a social media component for professional development and practices: A design-based research study
Source: Data Brief. 2016 Dec 27;10:544–7. doi: 10.1016/j.dib.2016.12.039 (PMC5219639; doi:10.1016/j.dib.2016.12.039)
Supplement: Supplementary file 6 — Supplementary material [file mmc6.pptx]

## Slide 1
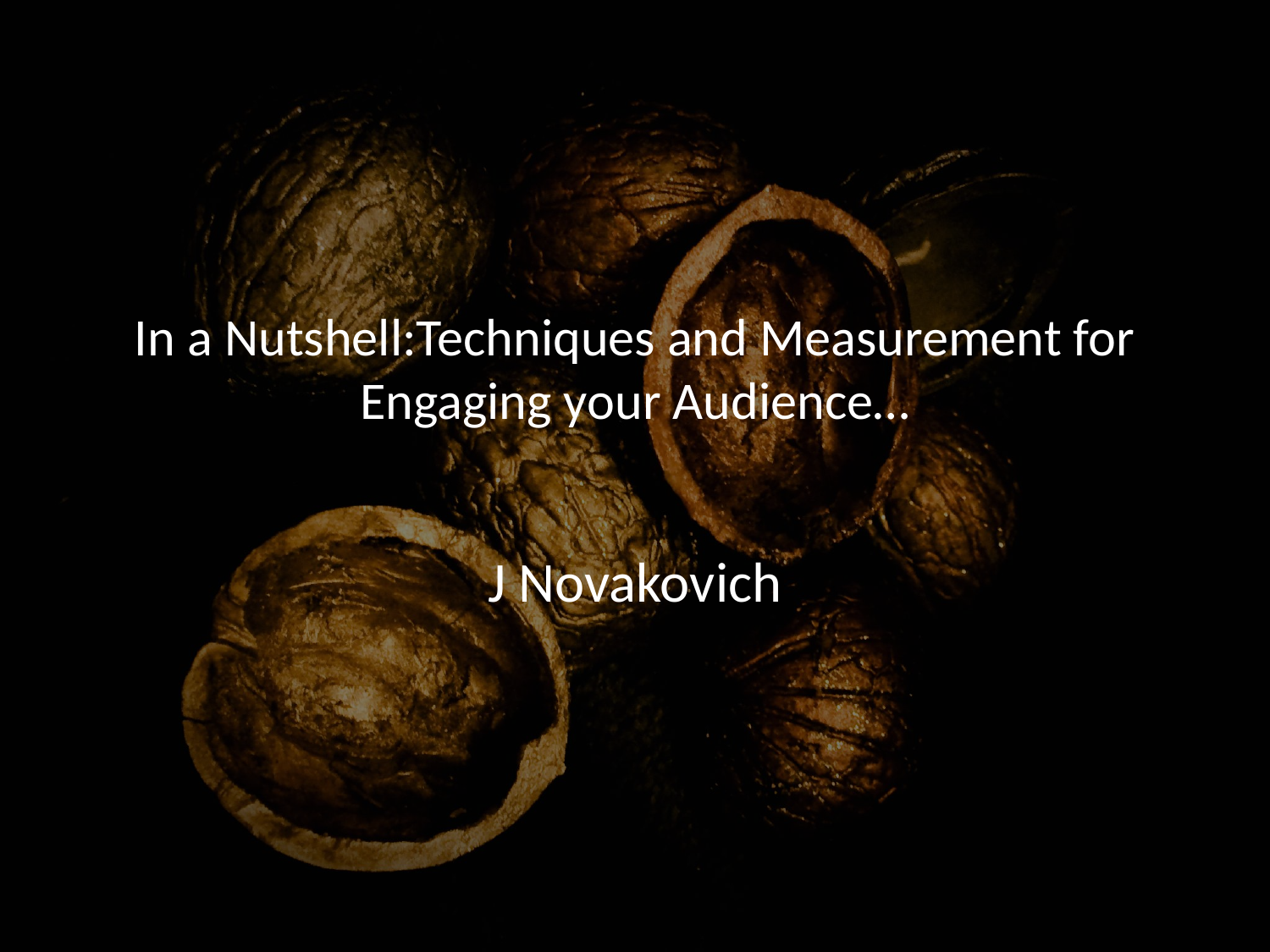

# In a Nutshell:Techniques and Measurement for Engaging your Audience…
J Novakovich

## Slide 2
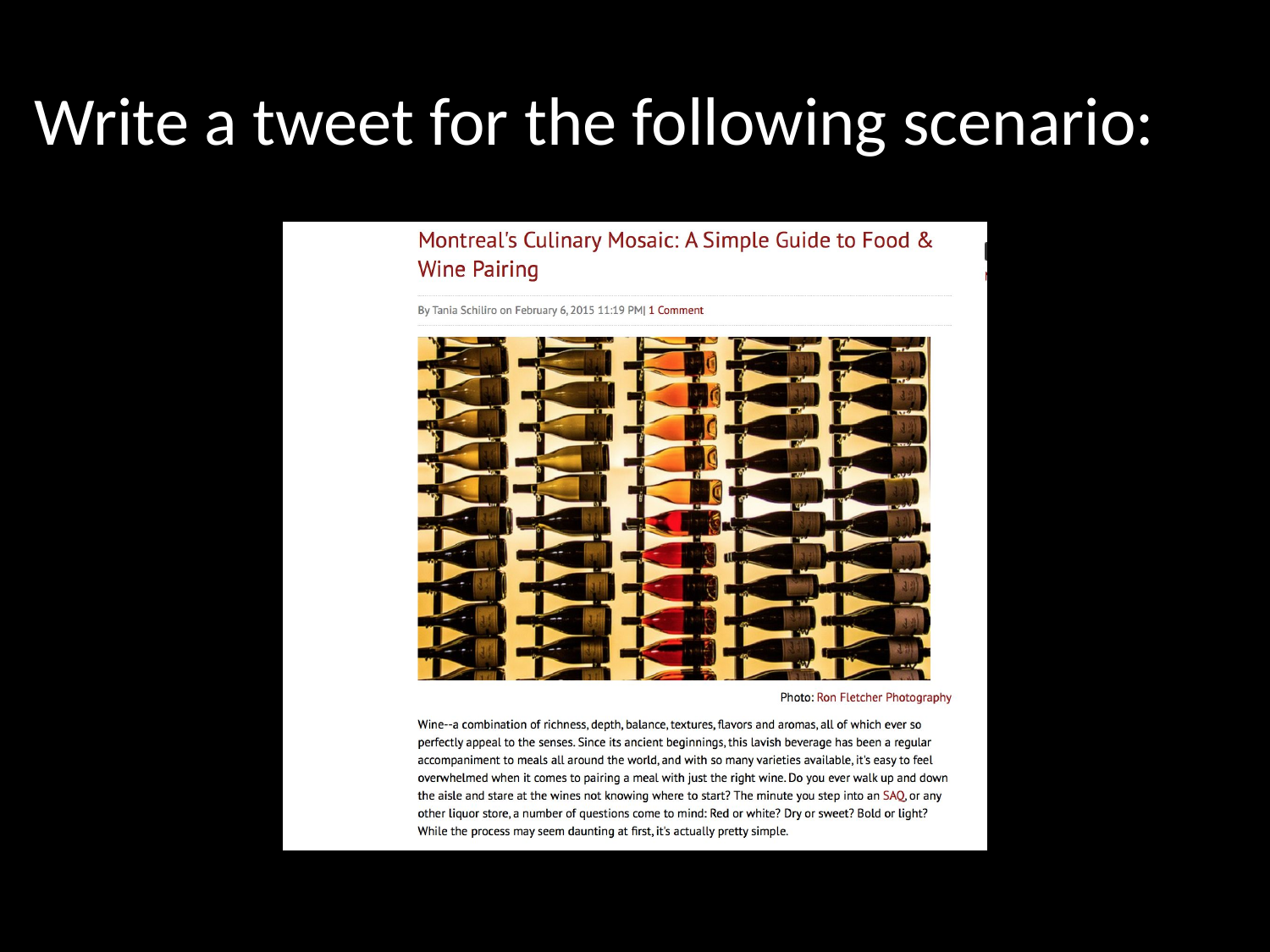

# Write a tweet for the following scenario:

## Slide 3
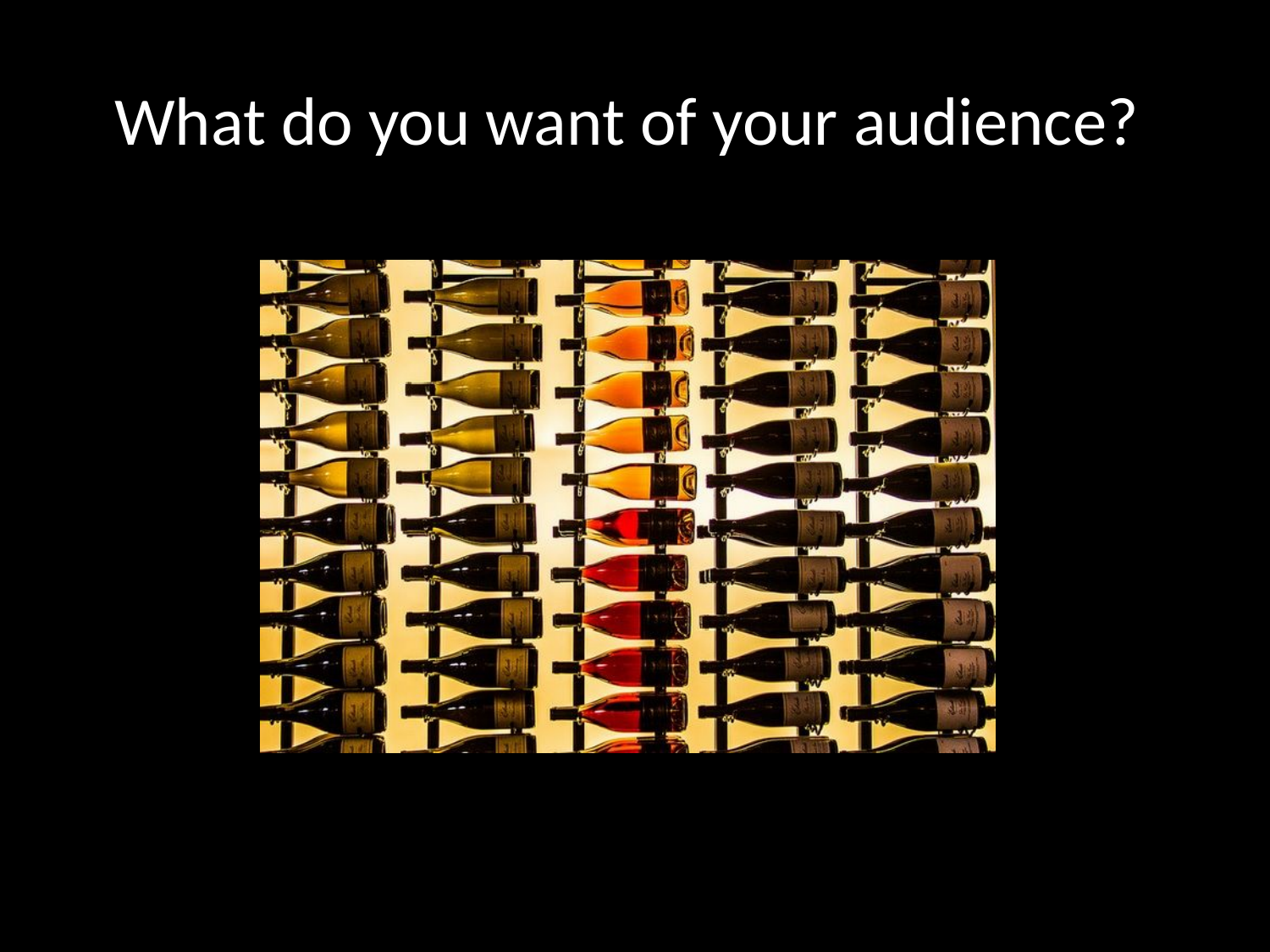

# What do you want of your audience?

## Slide 4
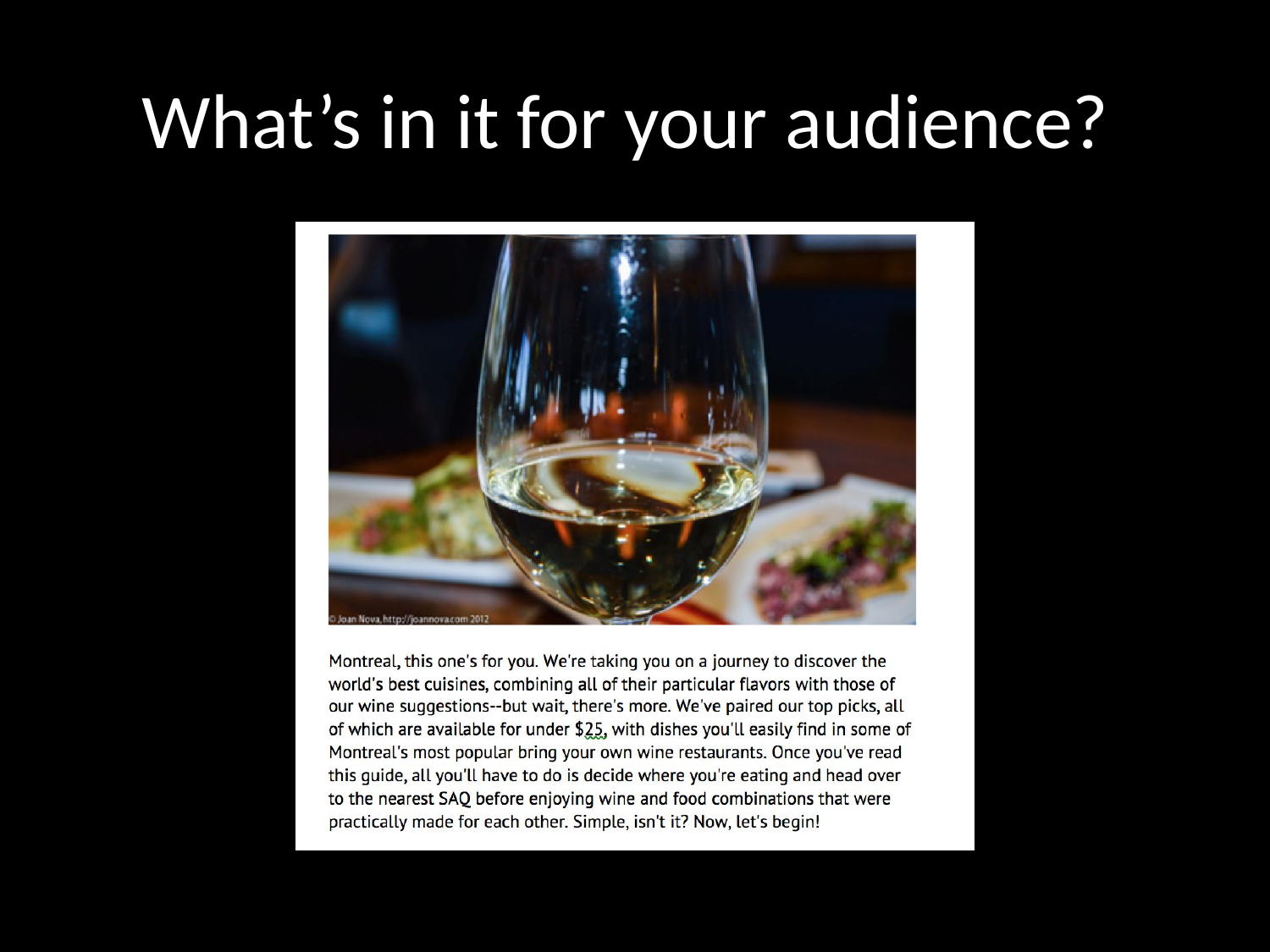

# What’s in it for your audience?

## Slide 5
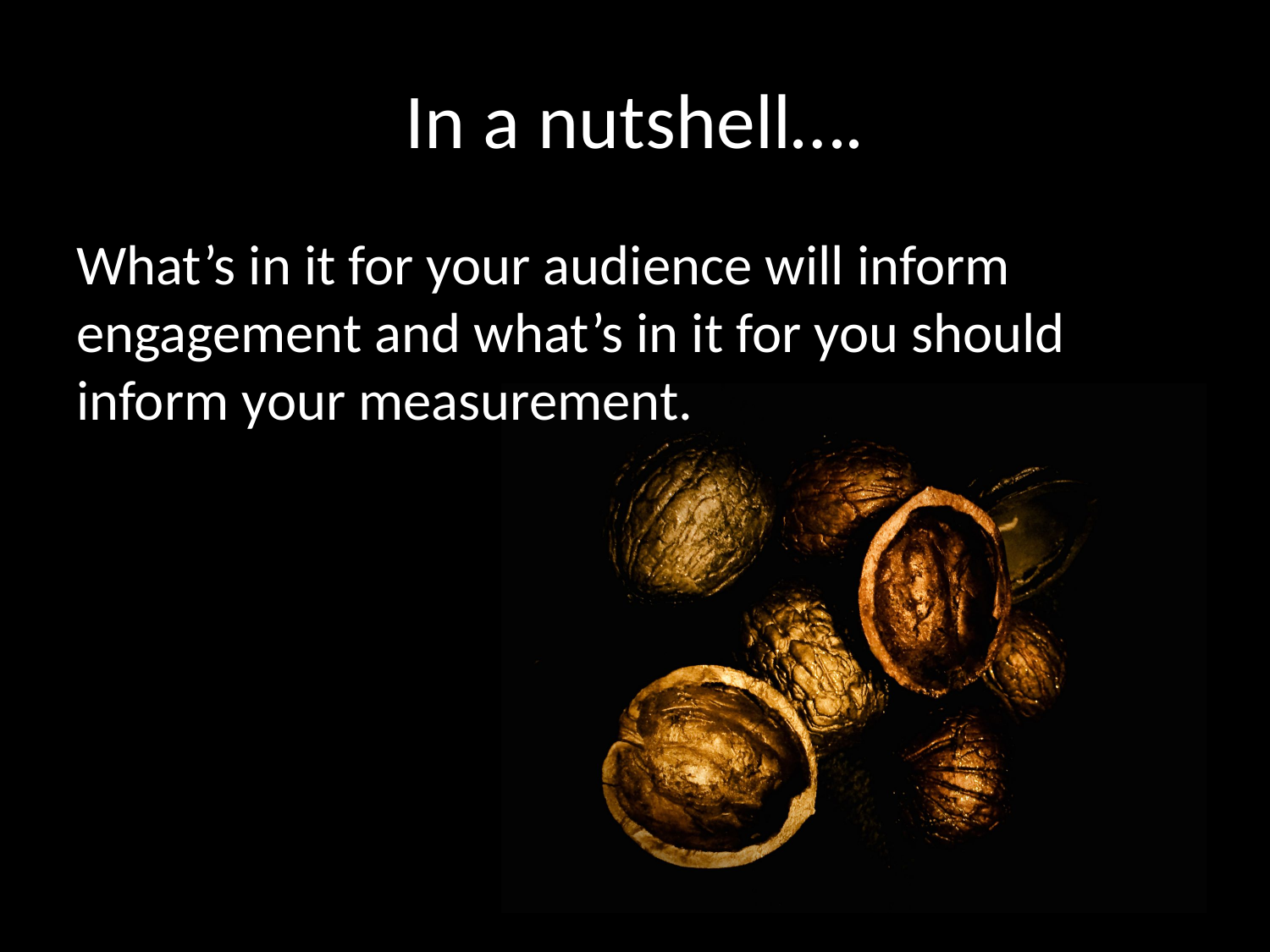

# In a nutshell….
What’s in it for your audience will inform engagement and what’s in it for you should inform your measurement.

## Slide 6
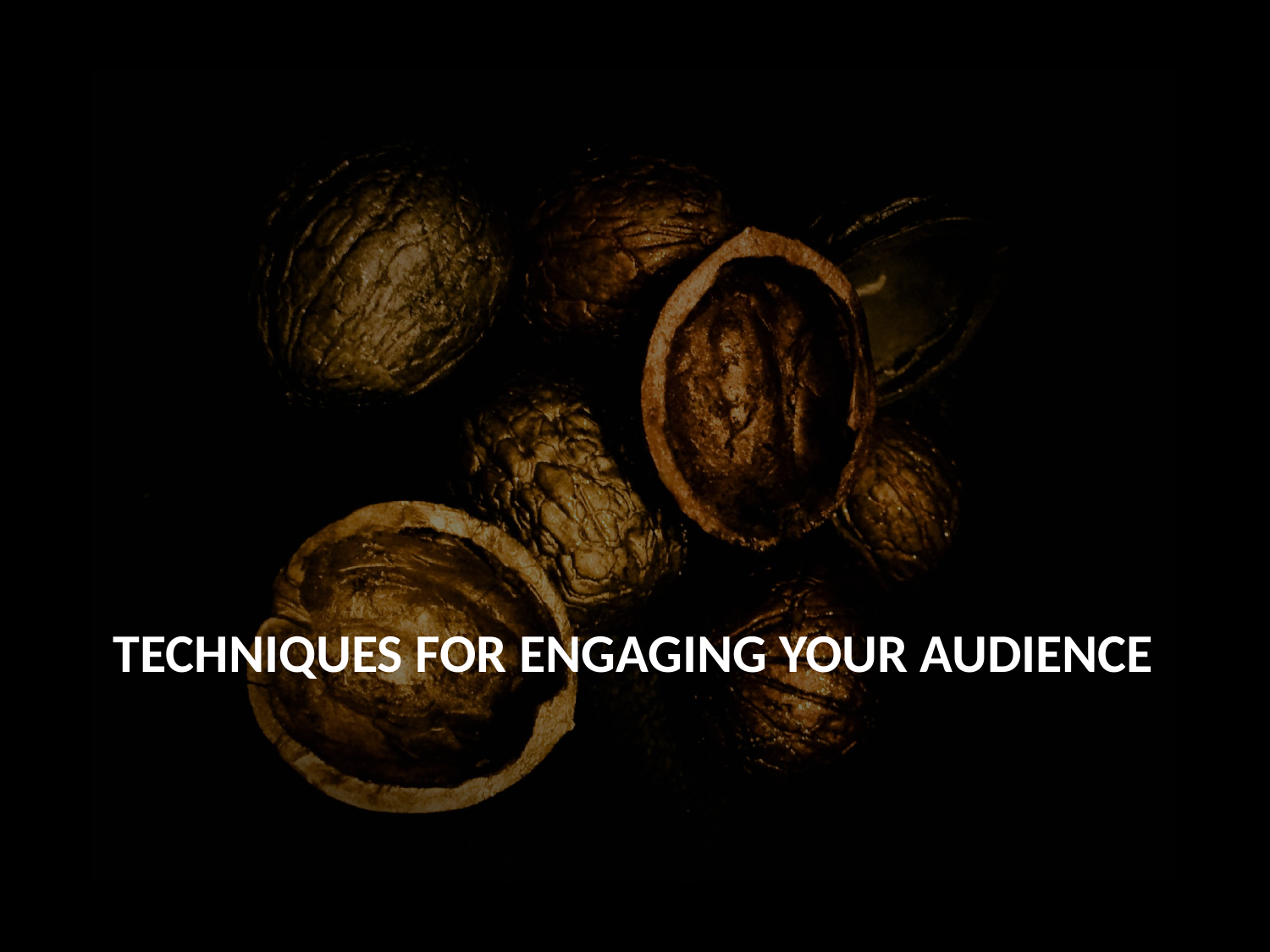

# Techniques for engaging your audience

## Slide 7
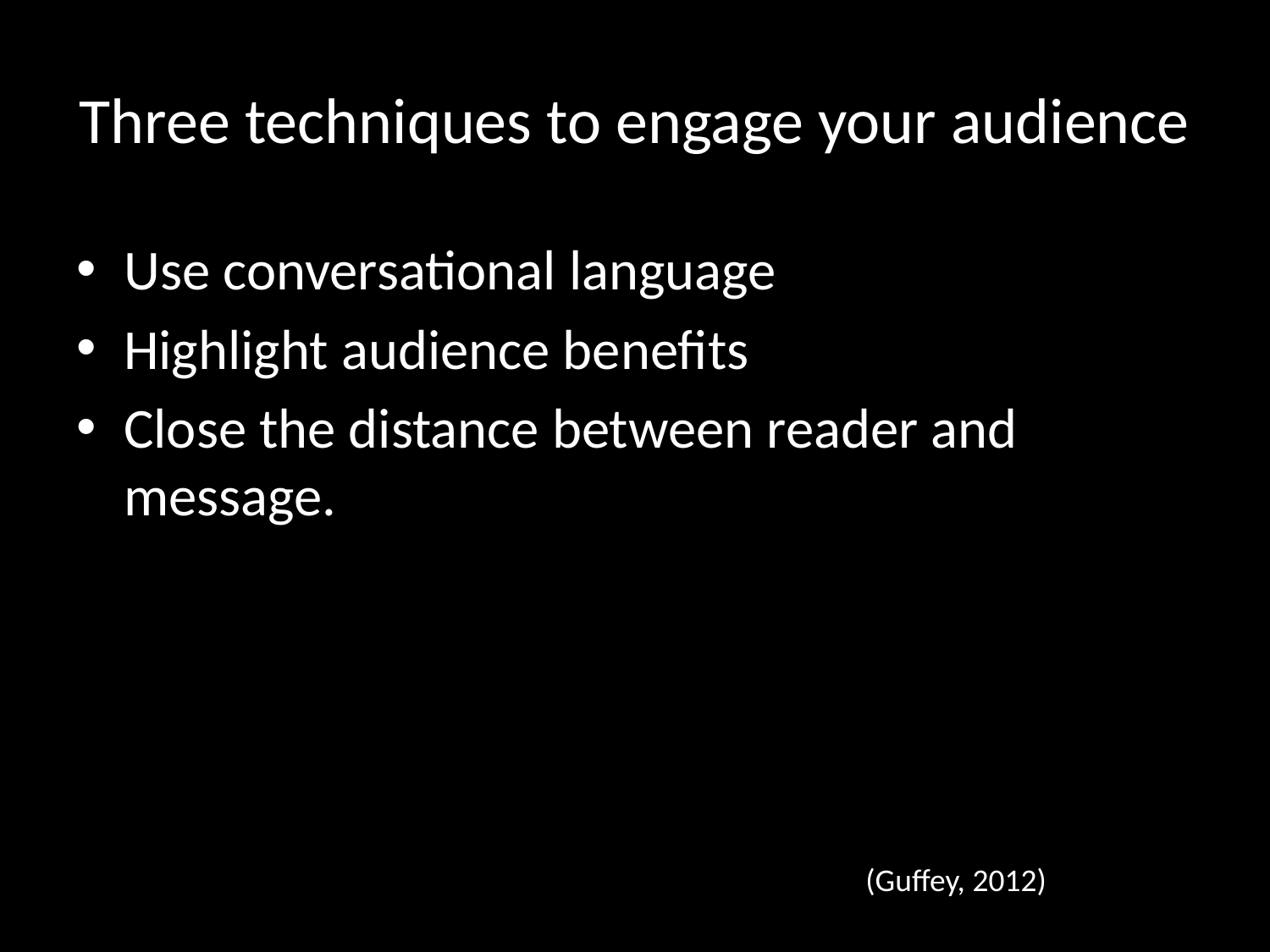

# Three techniques to engage your audience
Use conversational language
Highlight audience benefits
Close the distance between reader and message.
(Guffey, 2012)

## Slide 8
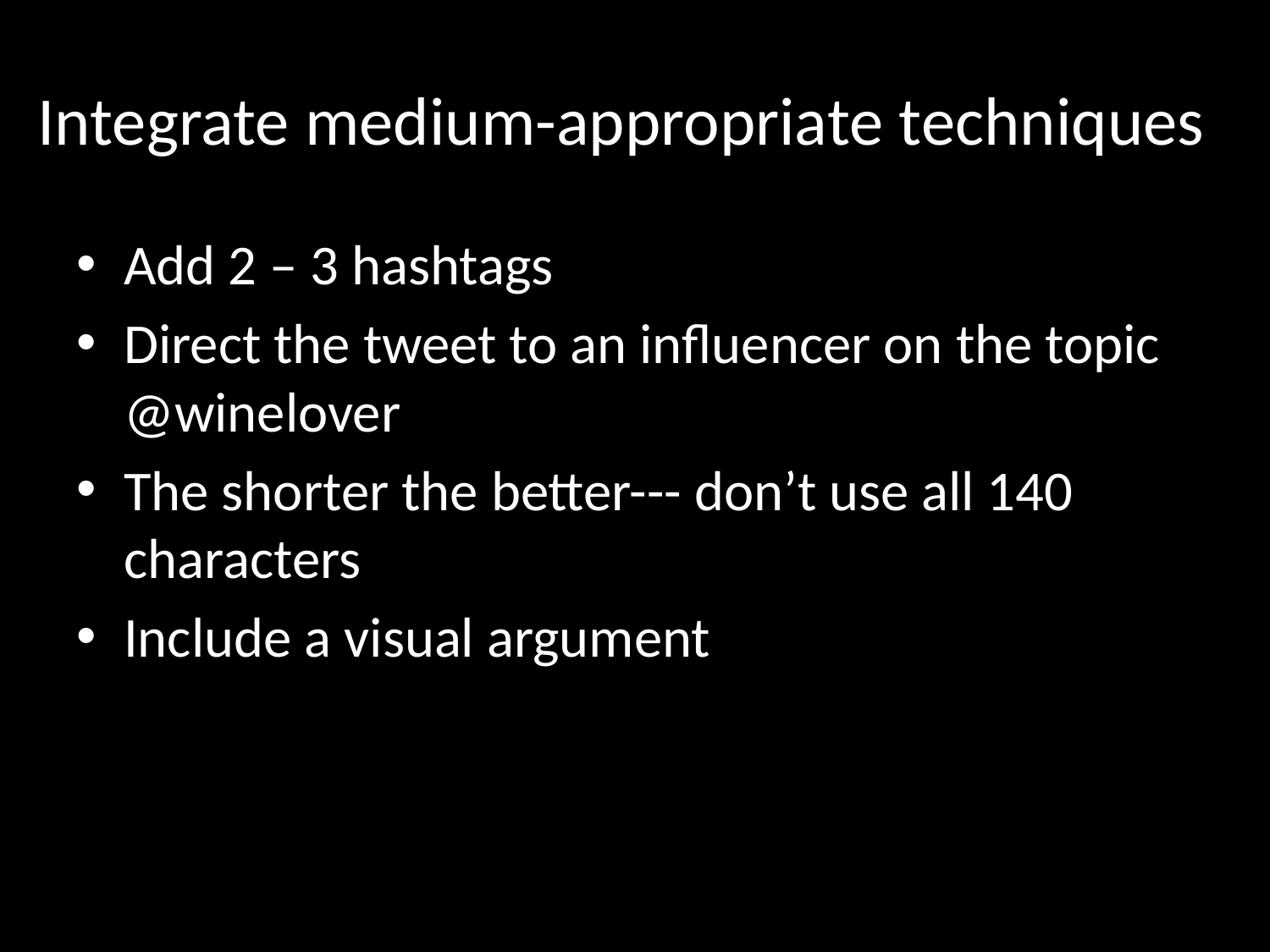

# Integrate medium-appropriate techniques
Add 2 – 3 hashtags
Direct the tweet to an influencer on the topic @winelover
The shorter the better--- don’t use all 140 characters
Include a visual argument

## Slide 9
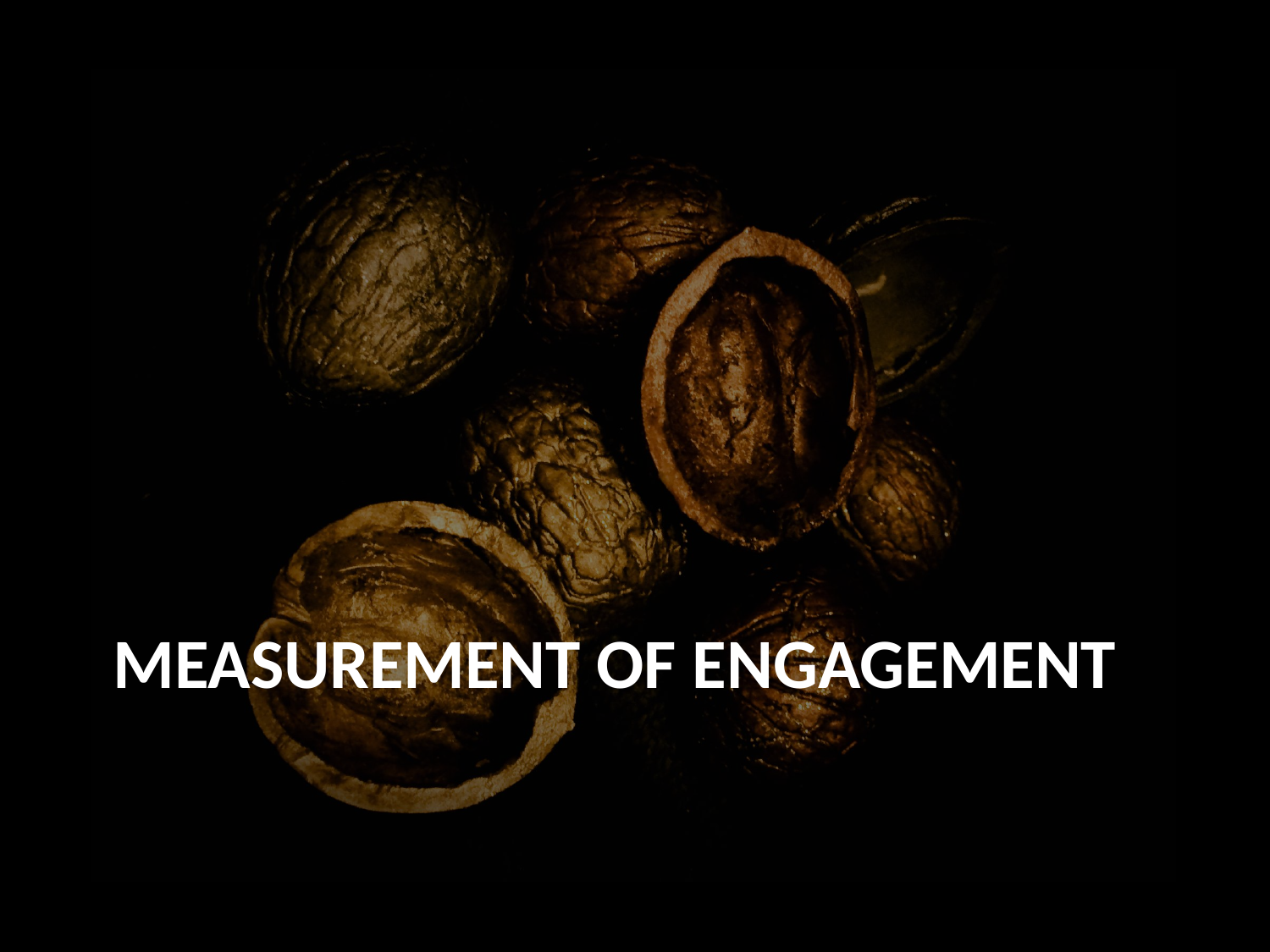

# Measurement of engagement

## Slide 10
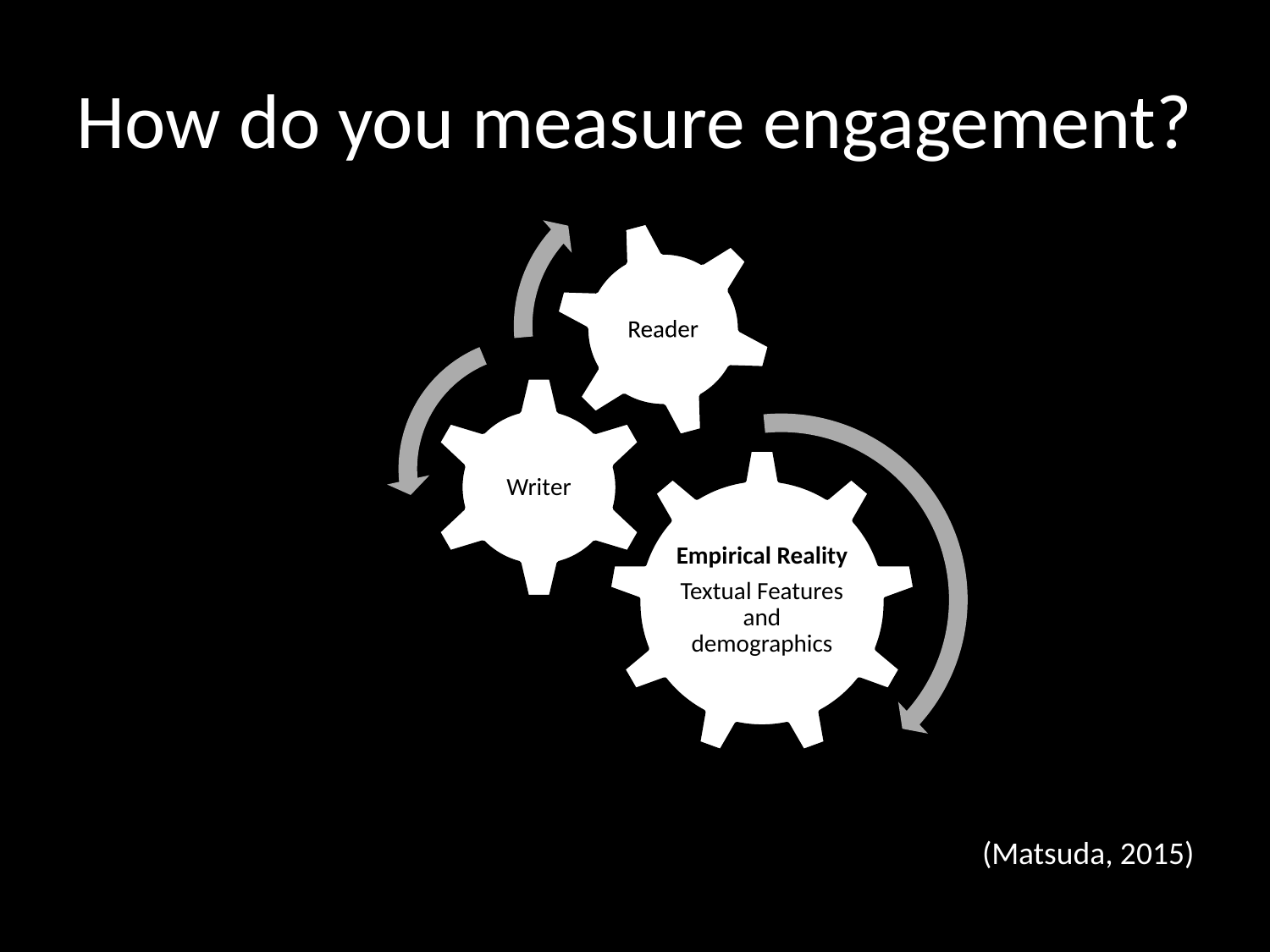

# How do you measure engagement?
(Matsuda, 2015)

## Slide 11
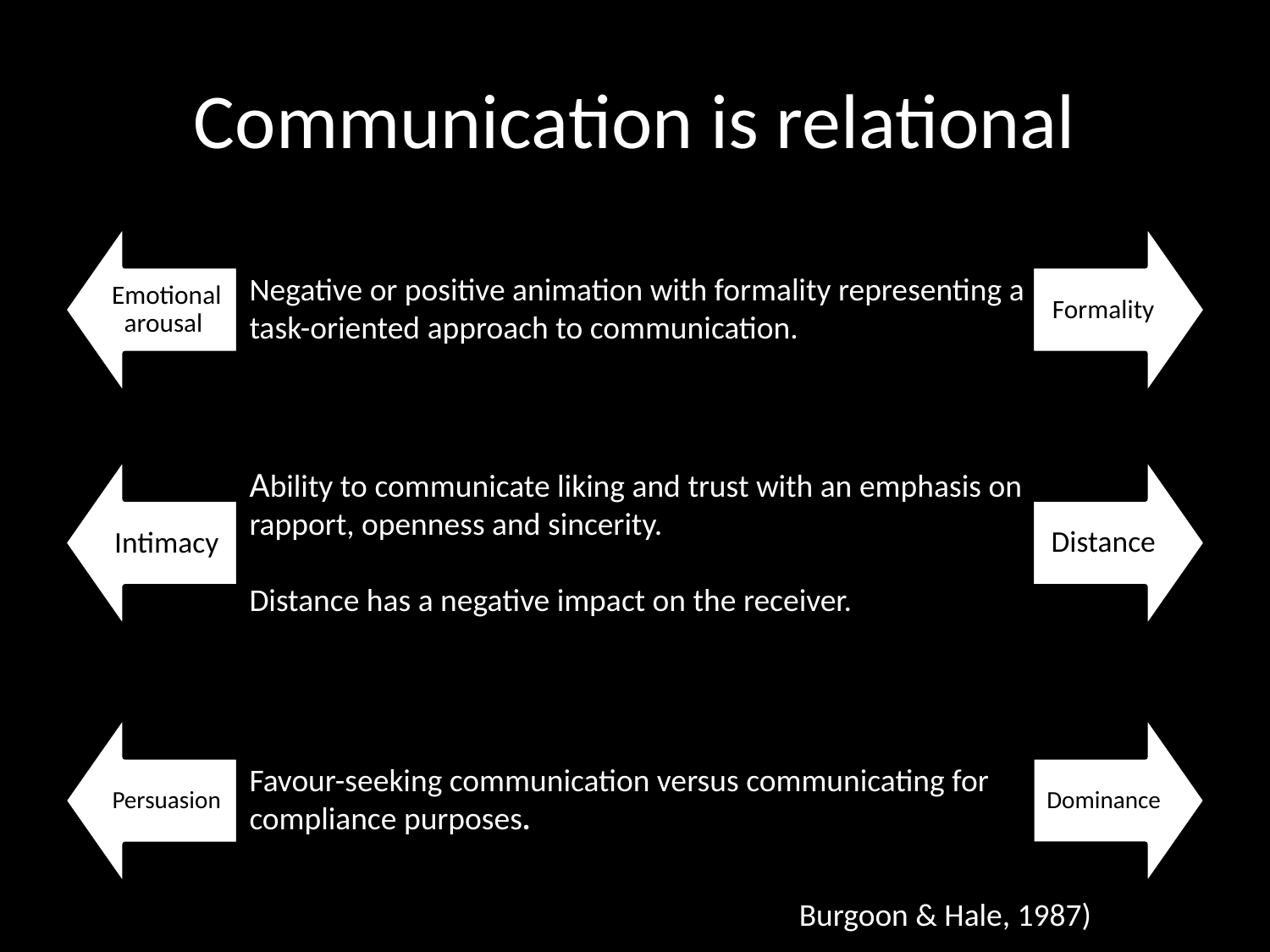

# Communication is relational
Negative or positive animation with formality representing a task-oriented approach to communication.
Ability to communicate liking and trust with an emphasis on rapport, openness and sincerity.
Distance has a negative impact on the receiver.
Favour-seeking communication versus communicating for compliance purposes.
Burgoon & Hale, 1987)

## Slide 12
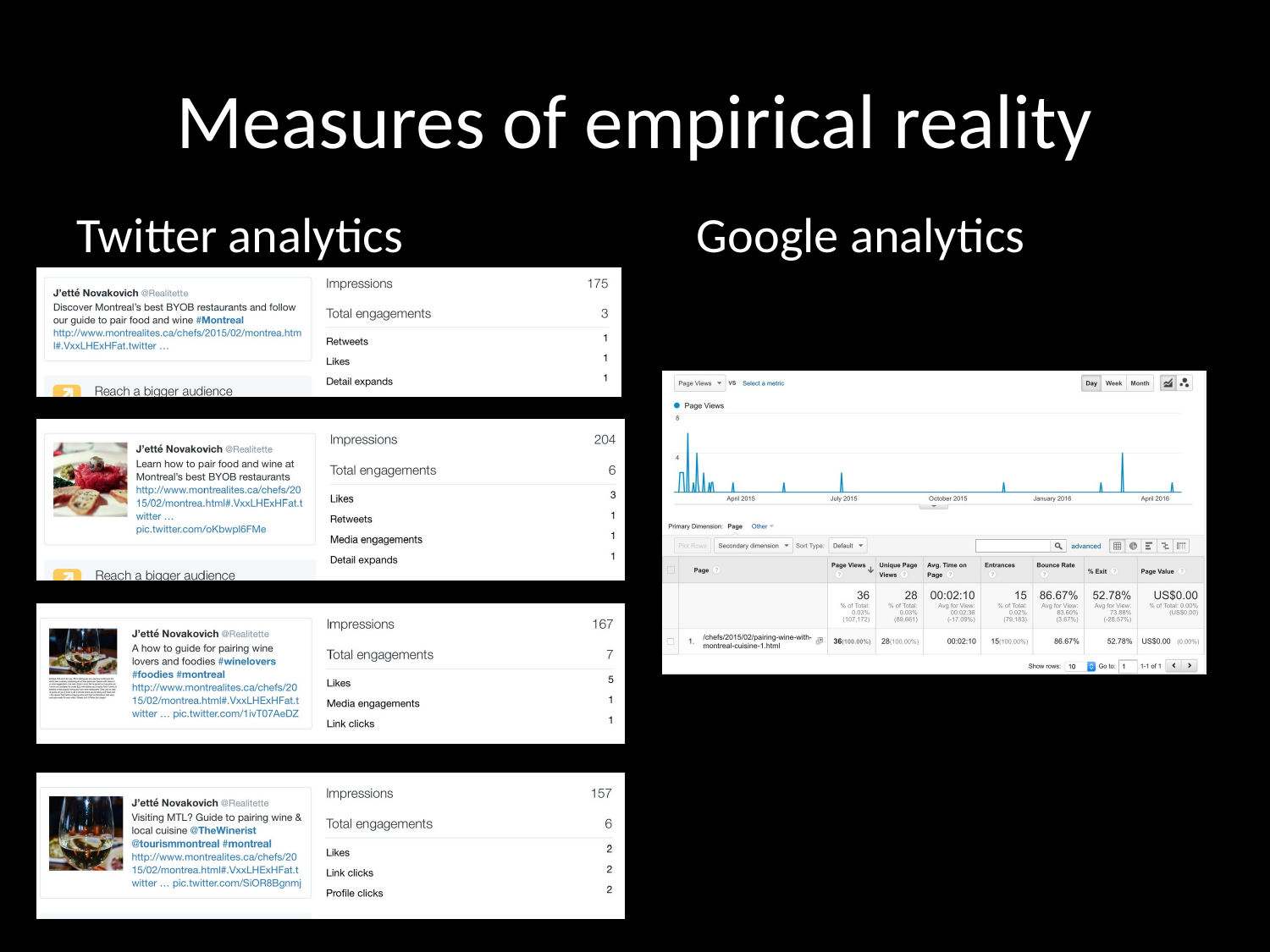

# Measures of empirical reality
Twitter analytics
Google analytics

## Slide 13
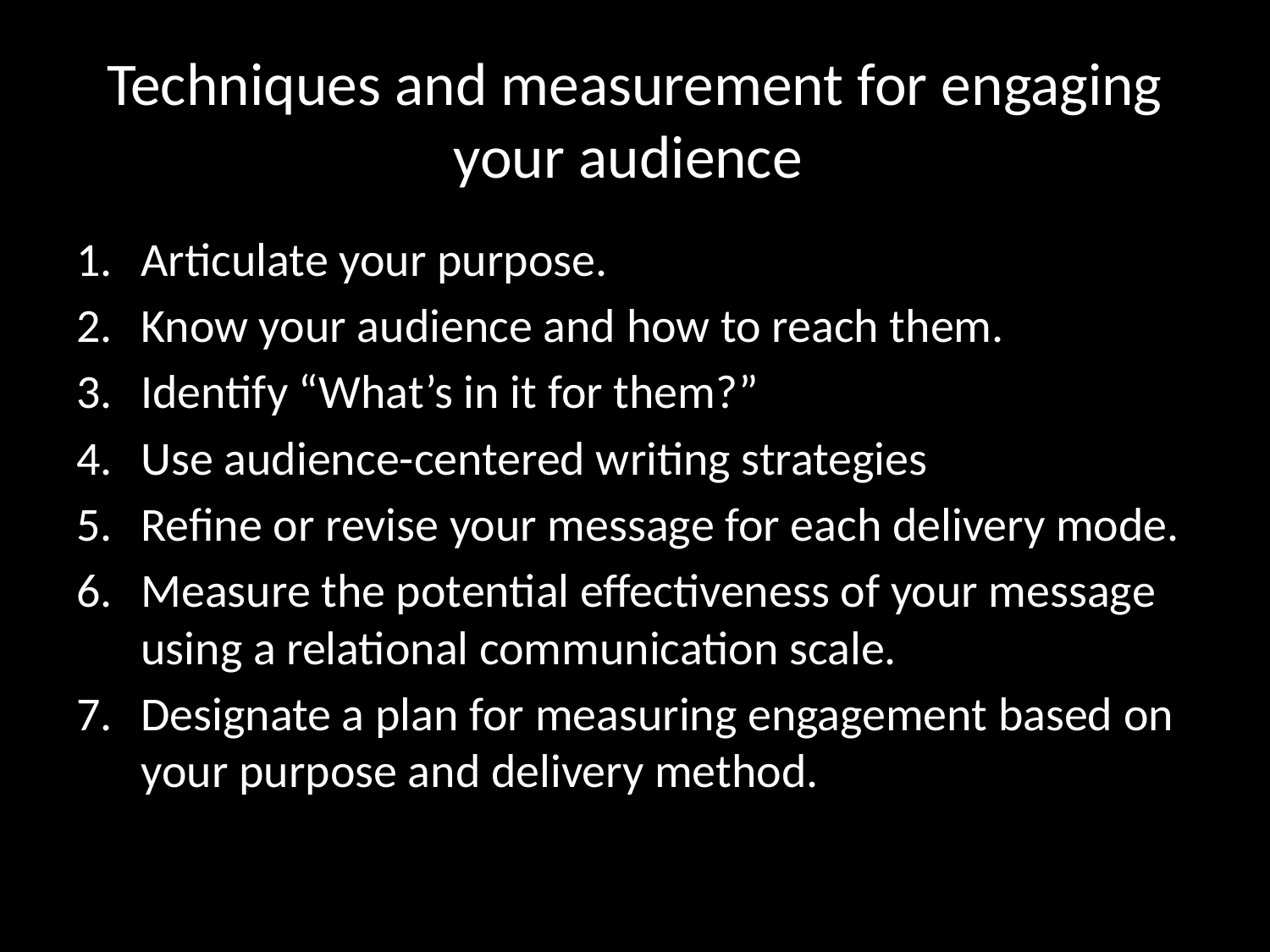

# Techniques and measurement for engaging your audience
Articulate your purpose.
Know your audience and how to reach them.
Identify “What’s in it for them?”
Use audience-centered writing strategies
Refine or revise your message for each delivery mode.
Measure the potential effectiveness of your message using a relational communication scale.
Designate a plan for measuring engagement based on your purpose and delivery method.

## Slide 14
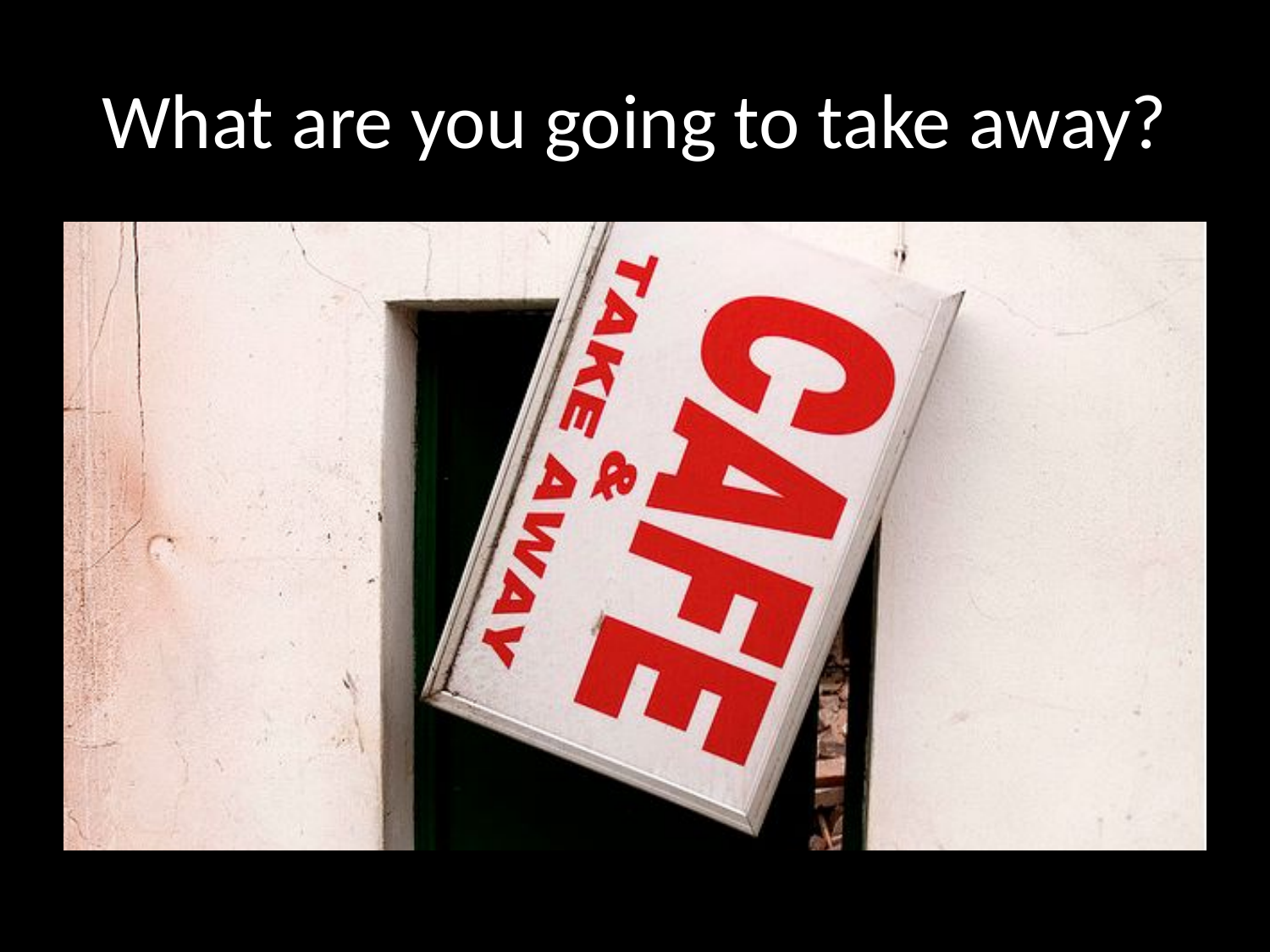

# What are you going to take away?

## Slide 15
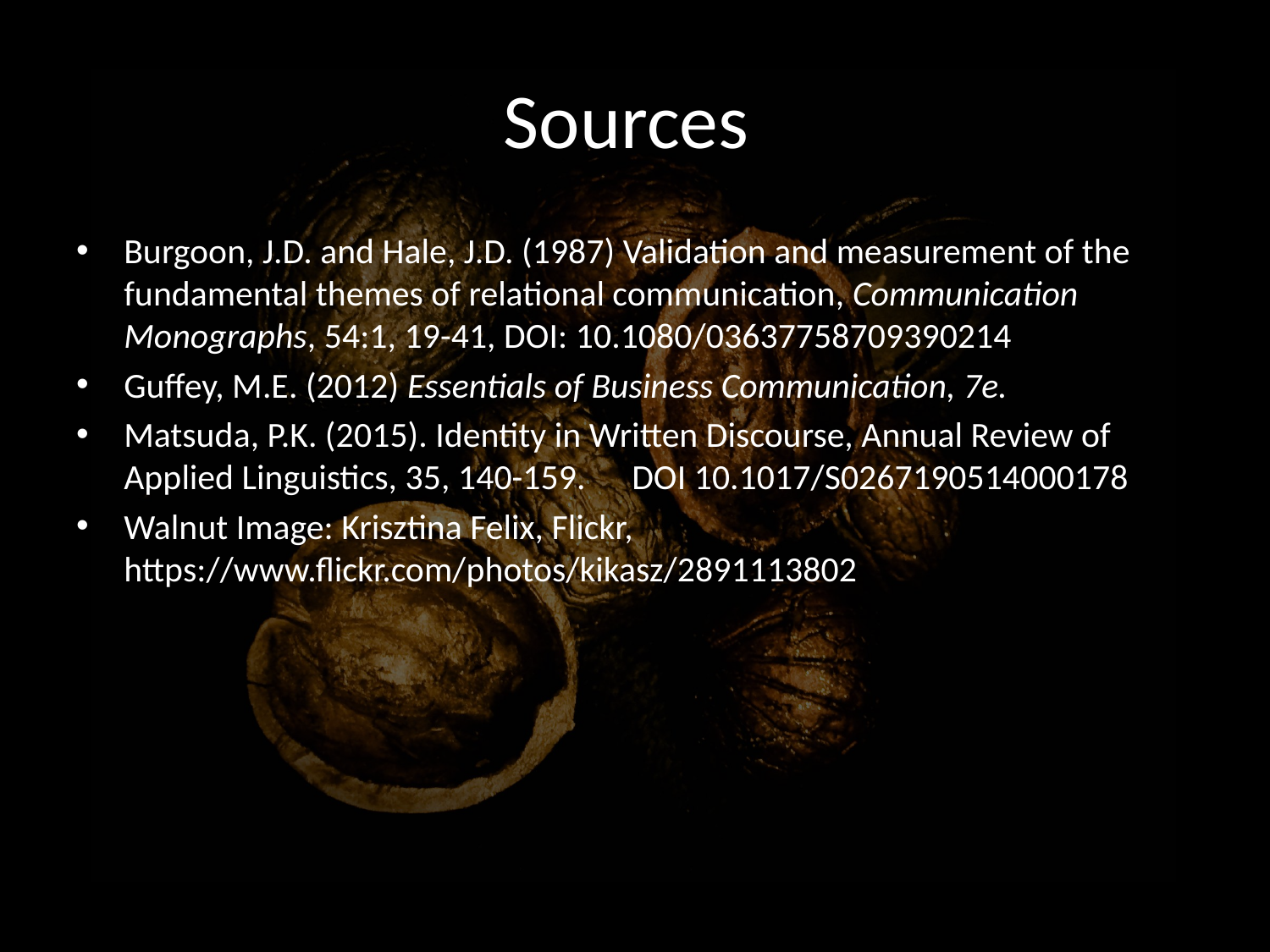

# Sources
Burgoon, J.D. and Hale, J.D. (1987) Validation and measurement of the fundamental themes of relational communication, Communication Monographs, 54:1, 19-41, DOI: 10.1080/03637758709390214
Guffey, M.E. (2012) Essentials of Business Communication, 7e.
Matsuda, P.K. (2015). Identity in Written Discourse, Annual Review of Applied Linguistics, 35, 140-159.	DOI 10.1017/S0267190514000178
Walnut Image: Krisztina Felix, Flickr, https://www.flickr.com/photos/kikasz/2891113802
